# Equity-promoting integer programming approaches for medical resident rotation scheduling

**DOI:** 10.1007/s10729-025-09736-4

**Published:** 2025-11-22

**Authors:** Shutian Li, Karmel S. Shehadeh, Frank E. Curtis, Beth R. Hochman

**Affiliations:** 1 Department of Industrial and Systems Engineering, Lehigh University, Bethlehem, PA USA; 2 Daniel J. Epstein Department of Industrial and Systems Engineering, University of Southern California, Los Angeles, CA USA; 3 Department of Surgery, Grossman School of Medicine, New York University, New York City, NY USA

**Keywords:** Resident scheduling, Fairness, Optimization, Integer programming, Operations research, Operations management

## Abstract

Motivated by our collaboration with a residency program at an academic health system, we propose new integer programming (IP) approaches for the *r*esident-to-*r*otation *a*ssignment *p*roblem (RRAP). Given sets of residents, resident classes, and departments, as well as a block structure for each class, staffing needs, rotation requirements for each class, program rules, and resident vacation requests, the RRAP involves finding a feasible year-long rotation schedule that specifies resident assignments to rotations and vacation times. We first present an IP formulation for the RRAP, which mimics the manual method for generating rotation schedules in practice and can be easily implemented and efficiently solved using off-the-shelf optimization software. However, it can lead to disparities in satisfying vacation requests among residents. To mitigate such disparities, we derive an equity-promoting counterpart that finds an optimal rotation schedule, maximizing the number of satisfied vacation requests while minimizing a measure of disparity in satisfying these requests. Then, we propose a computationally efficient Pareto Search Algorithm capable of finding the complete set of Pareto optimal solutions to the equity-promoting IP within a time that is suitable for practical implementation. Additionally, we present a user-friendly tool that implements the proposed models to automate the generation of the rotation schedule. Finally, we construct diverse RRAP instances based on data from our collaborator and conduct extensive experiments to illustrate the potential practical benefits of our proposed approaches. Our results demonstrate the computational efficiency and implementability of our approaches and underscore their potential to enhance fairness in resident rotation scheduling.

## Introduction

Upon graduating from medical school, new doctors join a certified residency program to fulfill specialty board certification requirements as part of the pathway to independent practice. Residency program lengths and structures vary by specialty, but most programs in the United States (U.S.) require around three to five years, commonly known as *post-graduate years* (PGY). For example, general surgery programs (including the one for Columbia University Irving Medical Center (CUIMC)) require five years. During each of these years, residents rotate through various hospital departments (services or divisions) to gain the required clinical training and specialization under the supervision of more senior physicians, while providing care to patients in these departments.

Medical schools administer residency programs in conjunction with their affiliated hospitals. The Accreditation Council for Graduate Medical Education (ACGME) sets the guidelines and expectations for residency programs in the U.S. Some of these guidelines apply to all specialties. Others are specialty-specific, such as the rotation period in each clinical setting. Residency programs use these guidelines to determine the list of rotations for each class of residents (PGY1–PGY5), then construct a yearlong *rotation schedule* that specifies the assignment of residents to *rotation* periods in different departments during the academic year. The schedule also specifies vacation time for each resident.

The exact approach for constructing the annual rotation schedule may vary between residency programs. However, most programs, including CUIMC, divide the academic year (e.g., 52–53 weeks at CUIMC) into time *blocks*, each consisting of several consecutive weeks within the academic year. The length of each block (i.e., the number of weeks) can be different. Assigning a resident to a block is equivalent to assigning this resident to a rotation period in a specific department. The length and structure of each rotation depend on the resident class, ACGME guidelines, residency program requirements, and staffing needs. Moreover, each resident must take a mandatory number of vacation weeks during the academic year that adhere to ACGME rules on rest periods. Residents often submit their requests for vacation time before the annual rotation schedule is constructed.

In most hospitals, the program director or chosen resident (e.g., the chief resident at CUIMC) manually constructs the annual rotation schedule for each class before the academic year starts. Constructing the annual rotation schedule is challenging, lengthy, and laborious for various reasons [1–4]. First, the schedule must adhere to accreditation standards and satisfy different class-specific rotation and educational requirements. Second, it must fulfill multiple staffing needs of different departments and affiliated hospitals. Third, it should accommodate residents’ vacation preferences. Finally, the rotation schedule should ensure equity among residents (e.g., no resident receives a more desirable vacation assignment than any other resident for the academic year) while also considering all other requirements and logistics preferences of the residency program. Indeed, the chief resident spends several weeks manually designing a rotation schedule that often fails to fulfill these constraints simultaneously. Additionally, manual methods often yield inequitable rotation schedules that are also difficult to adjust during the academic year. Mathematical formulations of the rotation scheduling problem are also challenging to solve.

These challenges underscore the need for computationally efficient and implementable optimization models that facilitate the automation of rotation scheduling. The ACGME has also called for methods to generate better and equitable rotation schedules to help improve residents’ satisfaction and retention [5–7]. However, as discussed in Section 2, rotation scheduling has received less attention than the shift scheduling problem. Moreover, existing formulations of the problem are challenging to solve, and few studies have attempted to address the issue of equity (fairness) among residents.

Motivated by these critical issues and our collaboration with CUIMC, this study develops, analyzes, and deploys new integer programming approaches for the *r*esident-to-rotation assignment problem (RRAP). Given sets of residents, resident classes, and departments, as well as a block structure for each class, rotation requirements for each class, program rules and requirements, and resident vacation requests, the RRAP is a feasibility problem that consists of finding a feasible rotation schedule specifying resident assignments to rotations and vacations. We first derive an IP formulation for the RRAP, which finds a feasible rotation schedule. As we later demonstrate, such a blind, feasible assignment to rotations yields inequitable rotation schedules, specifically disparities in satisfying vacation requests among residents, with some being given vacations according to their preferences and others being given vacations in weeks different from their preferred weeks. To address this issue, we derive an *equity-promoting* counterpart that ensures equity in the number of vacation requests granted based on residents’ preferences. Satisfying vacation preferences has been considered a criterion for other scheduling endeavors (see, e.g., [8–12]), and its absence has been noted as a drawback that impacts resident satisfaction, among others. As highlighted by [13], the challenge of accommodating vacation requests and equity in satisfying these requests has prompted the exploration of alternative, non-automated methods that lead to unfair schedules. This underscores the significance of improving equity in fulfilling vacation requests as an important indicator of schedule quality and residents’ satisfaction. Finally, we note that residents of the same class will be assigned the same workload in terms of required rotations throughout the academic year. Therefore, there is no disparity in this aspect.

### Contributions

Our main contributions, both methodological and practical, can be summarized as follows.

1. **New IP Approaches for the RRAP**.
  - We propose new IP models for the RRAP. These models adhere to ACGME guidelines and incorporate decisions and constraints related to class-specific rotation and program requirements, department staffing needs, vacation requirements, and other practical considerations. We first derive an IP formulation that finds a feasible rotation schedule that satisfies these constraints. This formulation mimics the manual method for generating rotation schedules in practice and provides the foundation for the equity-neutral and equity-promoting models. The equity-neutral model seeks to find an optimal rotation schedule that maximizes the total number of satisfied vacation requests. The equity-promoting counterpart additionally incorporates an inequity measure in the objective to minimize disparities in satisfying vacation requests among residents.
  - The feasibility problem and equity-neutral model can be easily implemented and efficiently solved using off-the-shelf optimization software, enabling the implementation of the model in practice. In contrast, the proposed equity-promoting RRAP model has two conflicting objectives: to maximize the total number of satisfied vacation requests and to minimize a measure of inequity in the distribution of satisfied requests among residents. Identifying the entire set of Pareto optimal (non-dominated) rotation schedules to the equity-promoting RRAP problem via traditional methods, such as the $\varepsilon$-constraint method, is computationally challenging. To address this challenge, we propose a computationally efficient *Pareto Search Algorithm* that can find the complete set of Pareto optimal solutions for large instances of the problem within a reasonable time suitable for practical implementation.
2. **User-Friendly Tool**. To facilitate adaptation in practice and automation of annual rotation schedule generation, we have developed a web-based, user-friendly tool that implements the proposed models using the Python language. The tool has two modules: input and output interfaces. The input interface allows users to download an Excel-based info template, fill in the required input parameters for the IP model, and upload it back into the tool. After uploading the template, users click the Generate Schedule function, which processes the data, solves the IP model using Gurobi, and generates the schedules. The output interface enables users to download the optimal solution using two Excel workbooks, one for the rotation schedule and the other for the associated vacation schedule.
3. **Computational Results and Insights**. We construct diverse RRAP instances based on data from CUIMC and conduct extensive experiments to illustrate the potential practical benefits of our proposed approaches. Our results (a) demonstrate the computational efficiency and implementability of our approaches and underscore their potential to enhance fairness in resident rotation scheduling; (b) show how equity-neutral models lead to inequitable rotation schedules and disparities in satisfying vacation requests; (c) illustrate how different choices of the inequity measure in the equity-promoting model result in different sets of Pareto-optimal rotation schedules; and (d) emphasize the importance of integrating rotation and vacation scheduling decisions to ensure equity among residents and show the negative consequences on fairness when adopting a sequential approach that separates the rotation and vacation scheduling decisions. Although inspired by our collaborating residency program, our approaches are generic and can be adapted by other programs to automate rotation scheduling.

### Structure of the paper

The remainder of the paper is organized as follows. In Section 2, we review the relevant literature. In Section 3, we provide details about CUIMC’s general surgery residency program. In Section 4, we present our proposed models. We present our Pareto Search Algorithm in Section 5. In Section 6, we present the RRAP tool. We present our numerical experiments and corresponding insights in Section 7. Finally, we draw conclusions in Section 8.

## Relevant literature

Personnel scheduling problems, including those related to medical professionals (e.g., physicians, nurses, etc.), have attracted much attention from the operations research community. These problems are challenging from a computational perspective, and each presents specific modeling challenges. The recent survey by Erhard et al. provides a comprehensive review of the literature on physician scheduling, including resident scheduling [1]. Two primary scheduling problems arise in resident training: rotation scheduling and shift scheduling. Typically, the former problem must be solved first before the academic year begins to construct the rotation schedule, which specifies residents’ assignments to specific departments within specific time blocks and vacation periods. The shift scheduling problem is then solved for each department to plan the daily and shift schedules for residents rotating in that department. As noted in [1], the rotation scheduling problem received significantly less attention than the shift scheduling problem. Additionally, limited research has been conducted on improving equity among residents. Our paper advances the literature on resident rotation scheduling by introducing new equity-promoting approaches. Next, we review relevant studies on rotation scheduling.

We emphasize upfront that there is no universally accepted approach to rotation scheduling. Moreover, the educational and training requirements of residency programs vary across institutions, hospital systems, and countries [14–16]. Different studies have focused on various aspects of the problem, resulting in distinct formulations. Most studies focused on constructing a feasible rotation schedule, considering hospital staffing needs, residency program educational requirements, and residents’ availability. Franz and Miller [17] is one of the earliest works on resident rotation scheduling. They considered resident preferences for each rotation in each month and proposed an IP model that assigns residents to rotation periods with the objective of maximizing the total weighted resident-to-rotation preferences. They first solved the LP relaxation of the IP model and then designed a rounding heuristic to find a feasible schedule. Guo et al. [2] provided a formal definition of the basic rotation scheduling problem, considering the assignment of residents to rotations and periods (13 periods, each lasting 4 weeks) while incorporating the basic constraints common to most residency programs. They showed that this problem is NP-hard and identified special cases solvable in polynomial time. Additionally, they proposed a greedy heuristic to find a feasible resident rotation schedule. Recently, Guo et al. [18] proposed a two-stage partial fixing approach to generate feasible annual block (rotation) schedules. The first stage assigns residents to a small set of predetermined services by solving a simplified problem relaxation. The second stage then finalizes the schedule by completing the remaining assignments based on the first stage’s solution.

As mentioned earlier, simply assigning residents to rotations and vacations can lead to inequitable schedules and, consequently, resident dissatisfaction [6]. However, most studies, including those discussed above, formulate the rotation scheduling problem as a feasibility problem or do not incorporate inequity measures into their models. Ensuring fairness among residents and accounting for their vacation preferences—the focus of this paper—have the potential to enhance resident satisfaction, educational experience, and performance, ultimately improving the quality of care and program’s reputation [5, 11, 19]. Next, we review the limited literature on equity considerations in resident rotation scheduling.

Recent studies in the resident scheduling literature have explored issues related to inequity from various angles, particularly in the context of assignment preferences (e.g., [13, 20, 21]) and workload distribution (e.g., [22–24]). However, many of these works do not explicitly define or quantify equity as a formal objective or constraint in their model formulations. Instead, they often focus on maximizing aggregate preference satisfaction or minimizing undesirable operational outcomes, which can indirectly influence fairness but do not directly address equity among individuals. For example, Cire et al. [25] studied the problem of assigning medical students to a series of rotations of different lengths at hospitals in various geographic locations. Their formulations—both a mixed-integer program (MIP) and a computationally efficient network-based model—focus on incorporating student preferences for rotation locations but do not explicitly address equity among them. Akbarzadeh and Maenhout [20] studied the problem of assigning medical students to specific disciplines and hospitals. They considered a residency program where students could customize their training by selecting a preferred subset of the disciplines from an elective list. They formulated the problem as an MIP that finds an optimal student assignment to disciplines and hospitals that simultaneously maximizes the total preference value over all students and minimizes the worst desire score among students, while also minimizing the usage of emergency capacity, departmental understaffing, and underutilization of the medical school capacity. Ultimately, they developed a heuristic to solve large-scale instances of the problem. In another study, Akbarzadeh and Maenhout [21] considered a similar problem and proposed an MIP to maximize the total weighted sum of the student desire scores and the minimum preference score across students. They solved the model using a customized branch-and-price algorithm.

Studies that explicitly incorporate fairness metrics or constraints include the following. Smalley and Keskinocak [4] discussed the issue of ensuring an equitable educational experience among residents during their rotation. They proposed an IP formulation that ensures that residents of the same class are assigned to rotations in the same set of departments. Bard et al. [23] proposed a MIP model to determine the annual block schedule of internal medicine residents that hierarchically minimizes the maximum deviation between the number of clinic sessions each resident must attend from the average; restricts the number of times residents are assigned to a night float block immediately before or after an intensive care unit block; and minimizes the maximum deviation between patients seen in clinic during any month over the year from the average. Bard et al. [23] solved their MIP using a heuristic that yielded alternative solutions. Castaño and Velasco [26] focused on fairness from the perspective of hospitals and proposed a MIP formulation to find resident rotation schedules that minimizes the variance in the number of residents assigned to each department. Castaño and Velasco developed a heuristic based on variable neighborhood searches to obtain near-optimal solutions.

Few studies have considered fairness in satisfying resident vacation requests. For example, Proano and Agarwal [7] focused on a rotation scheduling problem for internal medicine residents at Rochester General Hospital. They proposed a multi-stage, multi-objective optimization approach to generate yearlong weekly resident rotation schedules and vacation assignments. One of the key objectives is to maximize residents’ preferences for vacation time. Proano and Agarwal [7] employed the Analytical Hierarchy Process to evaluate and compare schedules across multiple criteria and identify those that are more equitable. Shahraki et al. [10] proposed a MIP model that finds optimal daily assignments and monthly rotations for residents. The objective is to maximize residents’ preferences on specific rotations and vacation times. The objective is to maximize residents’ preferences on specific rotations and vacation times. They also developed a decision-support tool that implements the model. Howard et al. [13] developed a rotation scheduling tool, the Automated Internal Medicine Scheduler. They discussed schedule quality, resident satisfaction, and perceptions of fairness after making the schedule by the tool.

Our paper advances the related literature in several ways. First, from a modeling perspective, we introduce and analyze new IP formulations for the RRAP: (i) a feasibility model that finds a feasible rotation schedule, (ii) an equity-neutral model that seeks an optimal rotation schedule that maximizes the total number of satisfied vacation requests, and (iii) an equity-promoting counterpart of (ii) that additionally incorporates an inequity measure in the objective to minimize disparities in satisfying vacation requests among residents. These models adhere to ACGME guidelines and incorporate constraints common to most residency programs in the U.S. Notably, unlike existing models that are computationally demanding and often require specially-developed algorithms or heuristics (e.g., [2, 17, 18]), our proposed feasibility and equity-neutral models can be easily implemented using standard optimization software and efficiently solve large problem instances; see Section 7.5. Moreover, most studies assume that the academic year is divided into multiple periods or blocks of the same length, typically a month or a week (e.g., [4, 18, 22, 23]). Such models can only be used for constructing rotation schedules with equal-length or similar rotations. In many residency programs, however, each class of residents may require a different training period in each department; that is, the set of blocks and their lengths (and hence the number of departments and the period of rotation in each) differ for each class. Our proposed models generalize existing models by accommodating block structure and length variations within resident classes.

Second, although some studies have attempted to incorporate inequity measures in their models, each study adopted a specific measure. For example, [22–24] used the maximum deviation from the mean as the inequity measure in their models, while [4] and [26] considered minimizing the difference between maximum and minimum outcomes. In contrast, our model allows for incorporating different inequity measures, and we compare rotation schedules constructed using a set of well-known measures.

Third, from an algorithmic standpoint, we introduce an innovative and computationally efficient Pareto Search Algorithm capable of finding the complete set of Pareto optimal rotation schedules to the equity-promoting IP within a time frame suitable for practical implementation. Most studies on multi-objective rotation scheduling (e.g., [4, 7, 20]) often employed the weighting method with fixed weights or $\varepsilon$-constraint methods. Our Pareto Search Algorithm offers several advantages over these traditional approaches. For example, the weighting method often fails to find the complete set of Pareto optimal solutions to bi-objective IP problems, such as our equity-promoting problem [27–29]. In contrast, our algorithm finds the entire Pareto optimal rotation schedules, which is particularly valuable in the rotation scheduling context as it provides decision-makers with a deeper insight into the trade-offs between the number of satisfied vacation requests and equity, enabling more informed schedule selection. Additionally, while the $\varepsilon$-constraint method typically demands significant computational effort to generate the entire set of Pareto optimal rotation schedules, our algorithm efficiently finds the entire set for large RRAP instances within a reasonable time (see Section 7.5).

Fourth, similar to [10, 13], we developed a new user-friendly tool implementing the proposed approach to automate the annual rotation schedule generation in practice. In contrast, [2, 7, 18, 20, 21, 25] did not develop decision-support tools, which limits the implementation of their proposed approaches in healthcare systems that lack ongoing access to support staff with optimization expertise. Finally, we illustrate the potential practical benefits of our proposed approaches using diverse instances and data from CUIMC. Our results demonstrate the computational efficiency and implementability of our approaches, underscoring their potential to enhance fairness in resident rotation scheduling.

## The general surgery residency program at CUIMC

Our proposed models are partly based on the general surgery residency program at CUIMC. In this section, we provide details about this program to lay the foundation for the subsequent discussions. This program is a five-year clinical training program. Year 1 (PGY1) residents are recent medical graduates joining the program, while year 5 (PGY5) residents are the most senior. The program offers categorical and preliminary positions. A categorical position is a five-year-long training required for board certification. In contrast, a preliminary position is one to two years of training generally before entry into advanced specialty programs. Residents from other surgical specialties (e.g., cardiac surgery) also do rotations in this program; hence, their partial rotations within the general surgery departments must be considered. Clinical training occurs at four sites: Milstein Hospital, Allen Hospital, University Hospital (Newark), and Overlook Hospital.

**Table 1:** An example of mandatory departments for each class of residents

Department	PGY1	PGY2	PGY3	PGY4	PGY5
Hepatopancreaticobiliary/Endocrine Surgery (HPB)	$\checkmark$		$\checkmark$		$\checkmark$
Colorectal Surgery (CR)	$\checkmark$		$\checkmark$		$\checkmark$
Breast/Surgical Oncology (Breast)	$\checkmark$		$\checkmark$		
Vascular Surgery (Vascular)	$\checkmark$		$\checkmark$	$\checkmark$	
Advanced Laparoscopic Surgery/Complex Hernia (Lap)	$\checkmark$		$\checkmark$		$\checkmark$
Pediatric Surgery (Peds)	$\checkmark$	$\checkmark$			
Overlook Hospital (Overlook)	$\checkmark$	$\checkmark$	$\checkmark$	$\checkmark$	
Surgical Intensive Care Unit (SICU)	$\checkmark$				
Thoracic Surgery (Thoracic)	$\checkmark$		$\checkmark$		
Night Float (Nights)	$\checkmark$			$\checkmark$	$\checkmark$
Acute Care Surgery Consult (ACS-Consults)		$\checkmark$			
Acute Care Surgery Operative (ACS-OR)		$\checkmark$			
Allen Hospital Consult (Consults)		$\checkmark$			
Cardiothoracic Intensive Care Unit (CTICU)		$\checkmark$			
Renal Transplant (Renal)			$\checkmark$		
Trauma–University Hospital (Trauma)			$\checkmark$		
Acute Care Surgery (ACS)				$\checkmark$	
Allen Hospital (Allen)				$\checkmark$	
Elective (Elective)				$\checkmark$	$\checkmark$

Before the academic year starts, the chief resident collects staffing requirements from each department (e.g., the minimum and maximum number of residents required to serve in each department), residents’ information, and vacation requests. Then, s/he divides the academic year of each class into several blocks based on the number of residents, educational requirements, ACGME rules, departments’ needs, and other considerations. Since each class of residents may require a different length of training period in each department, the set of blocks and their length are different for each class. For example, in the 2022–2023 academic year (PGY1, PGY2, PGY3, PGY4, PGY5) rotations were divided into (4, 6, 6 to 7, 7 to 8, 8 to 9) weeks long blocks.

Each class has different requirements regarding the department they must rotate in (mandatory departments). Table 1 provides an example of mandatory departments for each class. Categorical residents must attend the program during the academic year and rotate through all the mandatory departments for their classes. Generally, most residents rotate in each required department for their class once during the academic year. However, some classes may rotate more than once in some departments to satisfy their educational requirements or work time restrictions. For example, PGY2 needs to do service in the Acute Care Surgery (ACS) department twice (see Table 1). The first rotation is ACS-Consults, where residents learn clinical algorithms for common consults, including biliary disease, acute abdomen, bowel obstruction, and peripheral vascular disease. The second rotation is ACS-OR, which is more demanding. Specifically, in this rotation, residents perform all daytime operations on consults and elective patients in the Acute Care Service, manage the non-operative consult list, and rounds in the Surgical ICU on ACS inpatients.

**Fig. 1:**
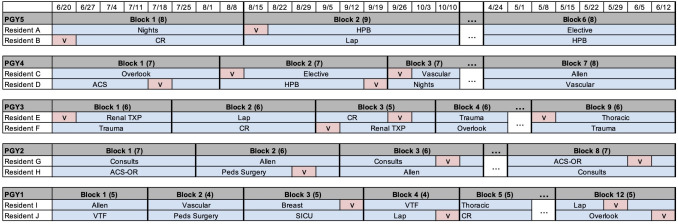
An example of rotation schedule of PGY1–PGY5 residents. There are ten residents in this example (two from each class). The number inside parentheses is the number of weeks in each block. Pink boxes marked with “v” indicate that a resident is on vacation that week

The typical number of residents in this program often ranges from 50-65. In Fig. 1, we provide a small illustrative example of block structure and rotation schedule. In this example, there are ten residents. For (PGY1, PGY2, PGY3, PGY4, PGY5), the number of blocks is (6, 7, 9, 8, 12) and each block contains (8 to 9, 7 to 8, 5 to 6, 6 to 7, 4 to 5) weeks. Each resident is assigned to one department in each block. For example, resident A (a PGY5 resident) is assigned to Nights in Block 1 and HPB in Block 2. Each resident has a vacation week (highlighted in pink and marked with “v").

## The resident-to-rotation assignment problem (RRAP)

In this section, we formally define the RRAP (Section 4.1). In Section 4.2, we present an IP formulation of the RRAP. Then, we present the equity-promoting counterpart in Section 4.3.

### Problem setting

Consider a residency program that requires residents to complete different services during the academic year in different departments (hospital units). We define *D* as the set of departments, *R* as the set of residents, and *E* as the set of resident classes (e.g., PGY1, PGY2, $\ldots$, PGY5). Since each class may require a different training period in each department, the program usually divides the academic year of each class (typically 52–53 weeks at CUIMC) into several blocks. Each block consists of several consecutive weeks. The length of each block (i.e., the number of weeks) can be different. Moreover, some blocks may have more weeks than others. Assigning a resident to a *block* is equivalent to assigning this resident to a *rotation* period in a given department. For each class $e\in E$, we define $B_{e}$ as the set of blocks for this class *e*, $R_{e}$ as the set of class *e* residents, and $W_{b,e}$ as the set of weeks in each block $b\in B_{e}$. Residents of the same class have the same block arrangement, but the block arrangements could vary between classes. We define the following sets that could be customized depending on residency program requirements, ACGME regulations, and resident’s class and availability:

- $D^{\text {req}}_{r}$ is the set of *required departments* that resident $r \in R$ must do, i.e., mandatory rotations.
- $D^{\text {imp}}_{r}$ is the set of *impossible departments* that residents $r \in R$ cannot work in, i.e., resident $r \in R$ cannot be assigned to any $d \in D^{\text {imp}}_{r}$.
- $D^{\text {busy}}$ is the set of *busy departments*. Such departments often have a heavy workload. Thus, if resident $r \in R$ is assigned to serve in a department $d \in D^{\text {busy}}$ in a week $w \in W$, then s/he cannot take a vacation during that week.
- $B^{\text {imp}}_{r}$ is the set of *impossible blocks* for resident $r\in R$, i.e., resident $r \in R$ cannot be assigned to any block $b \in B^{\text {imp}}_{r}$.

Residents of the same and different classes could have different required and impossible departments. Moreover, program requirements and regulations often limit the number of weeks each resident can serve in each department. Accordingly, we define parameter $T^{{\tiny min}}_{r,d}$ ($T^{{\tiny max}}_{r,d}$) as the minimum (maximum) number of blocks each resident $r \in R$ could work in department $d \in D$. On the other hand, each department may require a specific number of residents from each class. Accordingly, we define parameter $R^{{\tiny min}}_{e,d,b}$ ($R^{{\tiny max}}_{e,d,b}$) as the minimum (maximum) number of class *e*’s residents required to serve in department $d \in D$ in block $b \in B_{e}$.

Each resident should have a mandatory number of vacation weeks during the academic year (e.g., 2 or 4 weeks) that depends on ACGME rules and whether the resident is spending the entire academic year (e.g., categorical) or only a particular period (e.g., six months) in the program. Moreover, before the schedules are constructed, each resident submits their request for preferred vacation weeks. For each $r \in R$, we define $T^{\text {vac}}_{r}$ as the number of mandatory vacation weeks and $W^{\text {vac}}_{r}$ as the set of preferred vacation weeks. Moreover, we define $D^{\text {vac}}_{d,w}$ as the maximum number of residents on vacation in department *d* and week *w*. We define $R^{\text {vac}}_{r,b}$ as the maximum number of vacation weeks that resident *r* is allowed to take in block *b*. A complete list of our notation can be found in Table 2.

Given sets of residents, resident classes, departments, and blocks, as well as department types and staffing needs, rotation requirements for each class, and vacation requests, the RRAP consists of finding a feasible rotation schedule specifying each resident’s assignments to rotations and vacations. More intuitively, completing this scheduling problem can be visualized as filling the department name in each block as illustrated in Fig. 1 and specifying vacation weeks for each resident.

### The RRAP formulation

**Table 2:** Notation (RRAP)

Index sets	
*E*	the set of residents’ classes
$R_{e}$	the set of residents in class $e\in E$
*R*	the set of all residents, i.e., $R=\bigcup _{e\in E}R_{e}$
$B_{e}$	the set of blocks for residents in class *e*
$W_{b,e}$	the set of weeks in each block *b* of residents in class *e*
*W*	the set of weeks in the planning horizon, i.e., $W=\bigcup _{b\in B_{e}}W_{b,e},\forall e\in E$
*D*	the set of departments
$D^{\text {imp}}_{r}$	the set of resident *r*’s impossible working department
$D^{\text {req}}_{r}$	the set of resident *r*’s required working department
$D^{\text {busy}}$	the set of busy departments
$B^{\text {imp}}_{r}$	the set resident *r*’s impossible working blocks
$W^{\text {vac}}_{r}$	the set of weeks that resident *r* requests for vacations
Parameters	
$T^{{\tiny min}}_{r,d}$	resident *r*’s minimum required working time (in blocks) in department *d*
$T^{{\tiny max}}_{r,d}$	resident *r*’s maximum required working time (in blocks) in department *d*
$R^{{\tiny min}}_{e,d,b}$	minimum number of year *e*’s residents required in department *d* in block $b\in B_{e}$
$R^{{\tiny max}}_{e,d,b}$	maximum number of year *e*’s residents required in department *d* in block $b\in B_{e}$
$T^{\text {vac}}_{r}$	mandatory number of vacation weeks that resident *r* should take
$D^{\text {vac}}_{d,w}$	maximum number of residents in vacation in department *d* in week *W*.
$R^{\text {vac}}_{r,b}$	maximum number of vacation weeks that a resident *r* is allowed to take in block *b*.
Decision Variables	
$z_{r,d,b}$	equals 1 if resident *r* is assigned to department *d* in block *b*
$x_{r,d,w}$	equals 1 if resident *r* works in department *d* in week *w*
$v_{r,d,w}$	equals 1 if resident *r* has a vacation in week *w* in department *d*

In this section, we present our proposed IP formulation for the RRAP, which mimics the current manual method for generating the rotation schedule and provides the foundation for equity-promoting formulation presented in Section 4.3. We first introduce the sets, variables, and parameters defining this model. For each $e\in E$, $r\in R_{e}$, $b\in B_{e}$, and $d\in D$, we define a binary decision variable $z_{r,d,b}$, which equals 1 if resident *r* is assigned to department *d* in block *b*, and is zero otherwise. For each $r \in R$, $w\in W$, and $d \in D$, we define a binary decision variable $x_{r,d,w}$, which equals 1 if resident *r* works in department *d* in week *w*, and is zero otherwise. Finally, we define a binary decision variable $v_{r,d,w}$, which equals 1 if resident *r* has a vacation in a week *w*, and is zero otherwise, for all $r \in R$, $w\in W$, and $d \in D$. The RRAP formulation can be stated as the following feasibility problem.

1a $$\underset{\pmb {x},\pmb {z},\pmb {v}}{\text {maximize}/\text {minimize}} \ \ 0 \\$$

1b $$\begin{aligned}\text {s.t.}&\quad \underset{d\in D}{\sum }z_{r,d,b}\le 1,\quad \forall e\in E, r\in R_{e},b\in B_{e},\end{aligned}$$

1c $$\begin{aligned}&\quad \underset{b\in B_{e}}{\sum }z_{r,d,b}= 1,\quad \forall e\in E, r\in R_{e}, d\in D^{\text {req}}_{r},\end{aligned}$$

1d $$\begin{aligned}&\quad z_{r,d,b}=0,\quad \forall e\in E, r\in R_{e},b\in B_{e},d\in D^\text {imp}_{r},\end{aligned}$$

1e $$\begin{aligned}&\quad z_{r,d,b}=0,\quad \forall r\in R, b\in B^{\text {imp}}_{r},d\in D,\end{aligned}$$

1f $$\begin{aligned}&\quad \underset{{w\in W}}{\sum } \underset{{d\in D}}{\sum } v_{r,d,w}= T^{\text {vac}}_{r},\quad \forall r\in R,\end{aligned}$$

1g $$\begin{aligned}&\quad T^{\text {min}}_{r,d}\le \underset{b\in B_{e}}{\sum }z_{r,d,b}\le T^{\text {max}}_{r,d} \quad \forall e\in E, r\in R, d\in D^\text {req}_{r},\end{aligned}$$

1h $$\begin{aligned}&\quad R^{{\tiny min}}_{e,d,b}\le \underset{r\in R_{e}}{\sum }z_{r,d,b}\le R^{{\tiny max}}_{e,d,b},\quad \forall e\in E, d\in D,b\in B_{e}, \end{aligned}$$

1i $$\begin{aligned}&\quad v_{r,d,w}\le 1-z_{r,d,b},\quad \forall e\in E, r\in R_{e}, b\in B_{e}, w\in W_{b,e}, d\in D^{\text {busy}}, \end{aligned}$$

1j $$\begin{aligned}&\quad \underset{r\in R}{\sum }v_{r,d,w}\le D^{\text {vac}}_{d,w},\quad \forall d\in D, w\in W, \end{aligned}$$

1k $$\begin{aligned}&\quad \underset{w\in W_{b,e}}{\sum }\underset{d\in D}{\sum }v_{r,d,w}\le R^{\text {vac}}_{r,b},\quad \forall e\in E, r\in R_{e},b\in B_{e},\end{aligned}$$

1l $$\begin{aligned}&\quad z_{r,d,b}= v_{r,d,w}+x_{r,d,w},\quad \forall e\in E,r\in R_{e},b\in B_{e}, d\in D,w\in W_{b,e},\end{aligned}$$

1m $$\begin{aligned}&\quad z_{r,d,b},x_{r,d,w},v_{r,d,w}\in \{0,1\}, \\&\quad \forall e\in E,r\in R_{e}, d\in D, b\in B_{e},w\in W_{b,e}. \end{aligned}$$

Formulation 1a finds a feasible rotation schedule that satisfies all the residency program, hospital, and ACGME requirements. Constraints Eq. 1b ensure that each resident is assigned to at most one department in each block. Constraints Eq. 1c ensure that each resident is assigned to each required department exactly once. When a resident must serve in department $d \in D$ more than one time, one can easily replace constraints Eq. 1c by $\sum _{b\in B_{e}}z_{r,d,b}=\text {Req}$, where Req is the required number of rotations. Constraints Eq. 1d ensure that residents are not assigned to rotations in impossible departments. Similarly, constraints Eq. 1e ensure that residents are not assigned to impossible blocks. Constraints Eq. 1f ensure that each resident has the required vacation weeks. This constraint can be relaxed to $\sum _{w\in W}\sum _{d\in D}v_{r,d,w} \ge T^{\text {vac}}_{r}$ if the program permits residents to take more than the mandatory number of vacation weeks. Constraints Eq. 1g ensure that the length of resident *r*’s rotation in department *d* satisfies the required minimum $T^{{\tiny min}}_{r,d}$ and maximum $T^{{\tiny max}}_{r,d}$ rotation length. Constraints Eq. 1h ensure that the number of residents of class *e* working in department *d* satisfies $R^{{\tiny min}}_{e,d,b}$ and $R^{{\tiny max}}_{e,d,b}$ of that department. Recall that residents assigned to busy departments cannot take vacations during their rotation in these departments. Constraints Eq. 1i ensure this condition. Constraints Eq. 1j ensure that the total number of residents on vacation in department *d* and week *w* should be less than or equal to $D^{\text {vac}}_{d,w}$. Constraints Eq. 1k ensure that each resident $r \in R$ can take at most $R^{\text {vac}}_{r,b}$ weeks of vacations in each block. Finally, constraints Eq.1m ensure that if resident *r* is assigned to department *d* in block *b*, s/he either works or has a vacation for each week within block *b*. We highlight that, due to constraints Eq. 1m, we can relax the binary restriction on $\pmb {v}$.

We close this section by observing the following about formulation Eq. 1a. First, we model rotation and vacation requirements as hard constraints that must be respected to adhere to CUIMC residency program requirements and ACGME rules. Relaxing these constraints may result in failure to meet rotation and educational standards and potentially cause the program to lose its accreditation. Second, formulation Eq. 1a provides a blind feasible assignment to rotations and vacations, potentially leading to disparities in satisfying vacation requests among residents, with some residents assigned vacations in their preferred weeks and others assigned vacations in weeks different than their preferred weeks (see Section 7). Indeed, there is room to maximize the number of satisfied requests by considering the following formulation:

2 $$\begin{aligned} \underset{\pmb {v,x,z}}{\text {maximize}}&\Big \{f_{1}(\pmb {v}):=\underset{r\in R}{\sum }\ \underset{d\in D}{\sum }\ \underset{w\in W^{\text {vac}}_{r}}{\sum }v_{r,d,w} \Big | (\pmb {v}, \pmb {x}, \pmb {z}) \in \{ (1b)-(1m)\} \Big \}. \end{aligned}$$

However, as we later show in Section 7, similar to formulation Eq. 1a, formulation Eq. 2 may result in a disparity in satisfying vacation requests because it has no measure to ensure a fair vacation schedule. In what follows, we call formulation Eq. 2 an equity-neutral formulation. To mitigate this, in the next section, we derive an equity-promoting counterpart that maximizes the number of satisfied requests and minimizes a measure of inequity in the number of vacation requests granted based on residents’ preferences. Finally, we note that residents of the same class will have the same workload in terms of the assignment to required rotations during the academic year; thus, there is no unfairness in this aspect.

### The equity-promoting formulation

**Table 3:** Inequity measures

Index	Name of the measure	Mathematical expression
(1)	Range	$\max \limits _{r\in R}u_{r}-\min \limits _{r\in R}u_{r}$
(2)	Gini deviation (Gini)	$\sum \limits _{r\in R}\sum \limits _{r'\in R}|u_{r}-u_{r'}|$
(3)	Maximum pairwise deviation (MaxPair)	$\max \limits _{r\in R}\max \limits _{r'\in R}|u_{r}-u_{r'}|$
(4)	Absolute deviation from mean (MeanDev)	$\sum \limits _{r\in R}|u_{r}-\bar{u}|$
(5)	Maximum absolute deviation from mean (MaxMeanDev)	$\max \limits _{r\in R}|u_{r}-\bar{u}|$
(6)	Maximum sum of pairwise deviation (MaxSumPair)	$\max \limits _{r\in R}\sum \limits _{r'\in R}|u_{r}-u_{r'}|$
(7)	Sum of Maximum pairwise deviation (SumMaxPair)	$\sum \limits _{r\in R}\max \limits _{r'\in R}|u_{r}-u_{r'}|$

In this section, we derive an equity-promoting IP model for the RRAP. First, we introduce additional notations that are needed to define our equity-promoting model. We define $$u_r$$ as the number of satisfied vacation requests for resident $$r \in R$$, i.e., $$u_r=\sum _{d \in D}\sum _{w \in W^{\text {vac}}_{r}} v_{r,d,w}$$, where $$W^{\text {vac}}_{r}$$ is the set of preferred vacation weeks for resident *r*. We let $$\bar{u}=\sum _{i=1}^{|R|} u_i/|R|$$ represent the mean of $$\pmb {u}:=[u_1, \ldots , u_{|R|}]^\top$$. Finally, we let $$\phi : \mathbb {R}^{|R|\times |D|\times |W|} \rightarrow \mathbb {R}$$ represent an inequity measure. There is a wide variety of notions and measures in the literature to gauge inequity (unfairness or inequality), each with distinct mathematical expressions and characteristics [30–33]. As noted in [30, 31], some of these measures pose computational challenges and are difficult to optimize. Given our focus on modeling and solving a real-world problem, we adopt a set of well-known and commonly used inequity measures (presented in Table 3) that lead to computationally tractable linear IP models. We employ these measures to gauge the level of inequity among residents in terms of the number of satisfied vacation requests. For example, given a vacation schedule $$\pmb {v}$$, we can compute the Gini deviation in the satisfied vacation requests as $$\phi (\pmb {v})=\sum _{r\in R}\sum _{r'\in R, r' \ne r}|\sum _{d\in D}\sum _{w\in W^{\text {vac}}_{r}}v_{r,d,w}$$$$-\sum _{d\in D}\sum _{w\in W^{\text {vac}}_{r'}}v_{r',d,w}|$$. Note that $$\phi (\pmb {v})=0$$ implies perfect equity, whereas a larger value $$\phi (\pmb {v})>0$$ indicates a higher degree of inequity. Hence, to promote equity, one could consider minimizing $$\phi (\pmb {v})$$, i.e.,

3 $$\begin{aligned} \mathop {\mathrm {\text {minimize}}}\limits \limits _{\pmb {z}, \pmb {v}, \pmb {x}} \big \{ \phi (\pmb {v})\big | (\pmb {z}, \pmb {v}, \pmb {x}) \in \{(1b)-(1m)\}\big \}. \end{aligned}$$

Formulation Eq. 3 finds an optimal rotation schedule that minimizes a measure of inequity in satisfied vacation requests. Our preliminary investigation suggests that such formulation leads to a rotation schedule with zero vacation requests satisfied. Intuitively, denying all vacation requests is the most equitable schedule, with $\phi (\pmb {v})=0$ for any $\phi$. While this is an equitable schedule, residents will be dissatisfied. Indeed, there is room to satisfy vacation requests and improve satisfaction while ensuring equity among residents. To do so, we incorporate both objectives: minimizing inequity in the number of satisfied vacation requests among residents and maximizing the number of satisfied requests. The resulting equity-promoting RRAP formulation is as follows.

4a $$\begin{aligned} \mathop {\mathrm {\text {maximize}}}\limits _{\pmb {v,x,z}}&\qquad f_{1}(\pmb {v}):=\sum _{r\in R}\sum _{d\in D}\sum _{w\in W^{\text {vac}}_{r}}v_{r,d,w} \end{aligned}$$

4b $$\begin{aligned} \mathop {\mathrm {\text {minimize}}}\limits _{\pmb {v,x,z}}&\qquad f_{2}(\pmb {v}):=\phi (\pmb {v}) \end{aligned}$$

4c $$\begin{aligned} \text {subject to:}&\qquad (\pmb {v}, \pmb {x}, \pmb {z}) \in \{(1b)-(1m)\}. \end{aligned}$$

Formulation Eq. 4a finds an optimal rotation schedule that simultaneously maximizes the total number of satisfied vacation requests (i.e., $f_{1}$) and minimizes a measure of inequity in satisfied requests among residents (i.e., $f_{2}$). For brevity, we relegate the formulation of the form Eq. 4a based on each measure in Table 3 to Appendix A. Note that one can employ formulation Eq. 4a to promote equity among residents irrespective of their classes or equity among residents of the same class (or group). In the former case, we evaluate inequity within the entire set of residents. In the latter case, we evaluate inequity within each group. For example, suppose that the program defines *G* groups of residents $\{R_g\}_{g \in G}$ based on some criterion (e.g., seniority), where $R_g$ is the set of residents belonging to group *g*. In this case, one can compute $\phi _g(\pmb {v})$ for each group of residents $R_g$ and use $f_{2}(\pmb {v})= \sum _{g \in G} \phi _g(\pmb {v})$ in Eq. 4b. We compare these approaches in Section 7.4.

We observe the following about formulation Eq. 4a. The two objectives $f_1$ and $f_2$ can be conflicting, i.e., improving one can entail deteriorating the other. Indeed, satisfying a larger number of vacation requests does not necessarily ensure equity in the number of satisfied vacation requests per resident. To see this, let us consider the following simple example. Suppose we have three residents (i.e., $R=\{1,2,3\}$), where each has one satisfied vacation request (i.e., $u_{r}=1,\forall r\in R$ and $f_1$=3). This is an equitable vacation assignment because all residents have the same number of satisfied requests. Indeed, the value of each inequity measure in Table 3 is zero under this vacation assignment, i.e., $f_2=0$. Suppose we can satisfy an additional vacation request for one of the residents and we choose to fulfill one more request of resident 1. In this case, $u_{1}=2$, $u_{r}=1$ for $r\in \{2,3\}$, and the total number of satisfied vacation requests increases to $f_1=4$. This is clearly inequitable as one resident has two requests granted while the other two have one request and the value of all inequity measures increases. For example, the Gini deviation (measure 2) increases from 0 (under the first assignment) to 4 (under the second assignment).

In the next section, we present methodologies for investigating the trade-off between $f_{1}$ (number of satisfied vacation requests) and $f_{2}$ (inequity) and accordingly obtain Pareto-optimal rotation schedules. Here, Pareto optimal (equivalently, efficient, non-dominated, or non-inferior) rotation schedules or solutions to Eq. 4a are solutions that cannot be improved in one objective function without deteriorating their performance in the other one. The weighting and $\varepsilon$-constraint methods are widely used iterative methods to identify Pareto optimal solutions for bi-objective IP problems, such as the equity-promoting RRAP. The $\varepsilon$-constraint method has several advantages over the weighting method. First, as pointed out by [28, 29] and recently shown in [27], the weighting method often fails to find the complete set of Pareto optimal solutions to multi-objective integer and mixed integer programming problems. In contrast, the $\varepsilon$-constraint method does not suffer from this pitfall. Note that identifying the entire set of non-dominated rotation schedules is desirable since it maximizes the decision-maker’s knowledge about the trade-offs between the two objectives. They can then choose the “*most preferred*” schedule. Second, in the weighting method, the scaling of the objective functions strongly influences the obtained results. Therefore, one must scale the objective functions to a common scale before forming the weighted sum. In the $\varepsilon$-constraint method, this is not necessary. Given these shortcomings and our collaborator’s interest in analyzing the entire non-dominated rotation schedules, we do not adopt the weighting method.

## Solution methodology

In this section, we present our proposed method that produces the entire set of non-dominated rotation schedules of the equity-promoting RRAP problem in Eq. 4a. In Section 5.1, we briefly discuss the traditional $\varepsilon$-constraint method and its challenges. Then, in Section 5.2, we present our proposed Pareto Search Algorithms.Finally, in Section 5.3, we present symmetry-breaking constraints to ensure that the block schedules are filled sequentially. We relegate all proofs to Appendix E-Companion Section B.

### $\varepsilon -$constraint method

In this section, we briefly discuss the classical $$\varepsilon$$-constraint method (see [29, 34] for detailed discussions). To facilitate the discussion, we first introduce some notation and define relevant terms. We define the feasible set of problem Eq. 4a as $$\mathcal {F}=\{\pmb {z}\in \mathbb {B}^{|R|\times |D|\times |B|}, \pmb {x}\in \mathbb {B}^{|R|\times |D|\times |W|},$$$$\pmb {v}\in \mathbb {B}^{|R|\times |D|\times |W|}\, |(1b)-(1m)\}$$. Definitions 5.1 and 5.2 introduce the notions of Pareto optimal solutions and non-dominated points, respectively.

#### Definition 5.1

(Pareto Optimal or Non-dominated Solutions) A feasible solution $$(\pmb {z},\pmb {x},\pmb {v})\in \mathcal {F}$$ is called a Pareto-optimal (or non-dominated) solution if there is no other feasible solution $$(\pmb {z}',\pmb {x}',\pmb {v}')\in \mathcal {F}$$ such that $$f_{1}(\pmb {v}')\ge f_{1}(\pmb {v})$$ and $$f_{2}(\pmb {v}')\le f_{2}(\pmb {v})$$. We define the set of Pareto-optimal solutions as $$\mathcal {F}_{p}=\{(\pmb {z},\pmb {x},\pmb {v})\in \mathcal {F}|f_{1}(\pmb {v})$$$$\ge f_{1}(\pmb {v}'),f_{2}(\pmb {v})\le f_{2}(\pmb {v}'),$$$$\forall (\pmb {z}',\pmb {x}',\pmb {v}')\in \mathcal {F}\}$$.

#### Definition 5.2

(Non-dominated Points) Given a Pareto-optimal solution $(\pmb {z},\pmb {x},\pmb {v})\in \mathcal {F}_{p}$, the corresponding objective value $(f_{1}(\pmb {v}),f_{2}(\pmb {v}))$ is called a non-dominated point. We call the set $\mathcal {P}=\{(f_{1}(\pmb {v}),f_{2}(\pmb {v}))^\top \in \mathbb {R}^2|(\pmb {z},\pmb {x},\pmb {v})\in \mathcal {F}_{p}\}$ the set of non-dominated points (Pareto front).

In the $\varepsilon$-constraint method, we optimize one objective function while constraining the value of the other objective function, requiring that the objective function value meets a threshold and iteratively adjusting that threshold. Specifically, the method produces the entire set of non-dominated solutions and points of problem Eq. 4a by solving a sequence of problems of the following form

5 $$\begin{aligned} \mathop {\mathrm {\text {minimize}}}\limits \limits _{\pmb {z}, \pmb {v}, \pmb {x}} \big \{ f_2(\pmb {v})\big | (\pmb {z}, \pmb {v}, \pmb {x}) \in \{(1b)-(1m)\}, f_{1}(\pmb {v})\ge V+\varepsilon \big \}, \end{aligned}$$

where $\varepsilon>0$ is a pre-defined small positive constant. Algorithm 1 summarizes the steps of the $\varepsilon$-constraint method. To initialize the algorithm, we solve the equity-neutral problem Eq. 2 and record the optimal value $\overline{V}$ (maximum number of vacation requests that can be satisfied). Then, the algorithm solves a sequence of problem Eq. 5 by successively adjusting (increasing) the parameter *V* in the RHS of the constrained objective $f_1$. Given that the minimum increase in the number of satisfied vacation requests in any feasible solution is one, we can set $\varepsilon =1$. In each iteration, Algorithm 1 seeks to identify a new feasible solution with one additional vacation request satisfied compared to the solution obtained from the previous iteration, where vacation requests are granted in a way that minimizes the inequity measure.

As is well-known, the traditional $\varepsilon$-constraint method may require substantial computational effort to generate the entire Pareto front [35, 36]. Indeed, our results in Section 7.5 suggest that implementing the $\varepsilon$-constraint method to solve Eq. 4a with some inequity measures such as Gini and MeanDev is computationally challenging. In particular, as the number of fulfilled vacation requests, denoted as *V*, increases, problem Eq. 5 becomes extremely challenging to solve. To address this challenge, in the next section, we present our proposed Pareto Search algorithm, which, as shown in Section 7 can find the entire Pareto front for large RRAP instances within a reasonable time.

**Algorithm 1:**
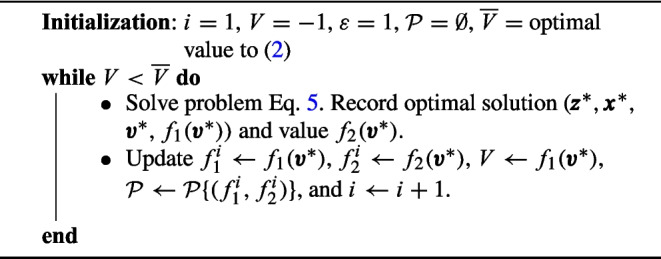
The $\varepsilon$-constraint method for the equity-promoting RRAP.

### Pareto search algorithm

In this section, we present our proposed Pareto Search Algorithm. We first identify several characteristics of the optimal solutions and Pareto front, which we exploit in our proposed algorithm.

Recall that the minimum value of any inequity measure in Table 3 is zero. In Proposition 1, we show that there exists a Pareto-optimal solution $(\pmb {z}^{0},\pmb {x}^{0},\pmb {v}^{0}) \in \mathcal {F}_{p}$ to Eq. 4b with $f_{2}(\pmb {v}^{0})=0$.

#### Proposition 1

An optimal solution $(\pmb {z}^{0},\pmb {x}^{0},\pmb {v}^{0})$ to the optimization problem

6 $$\begin{aligned} \mathop {\mathrm {\text {maximize}}}\limits _{\pmb {z},\pmb {v},\pmb {x}} \Big \{f_1(\pmb {v})\Big |(\pmb {z},\pmb {v},\pmb {x})\in (1b)-(1m),f_{2}(\pmb {v})=0 \Big \} \end{aligned}$$

is a Pareto-optimal solution to problem Eq. 4b, i.e., $(\pmb {z}^{0},\pmb {x}^{0},\pmb {v}^{0})\in \mathcal {F}_{p}$ and $(f_{1}(\pmb {v}^0),f_{2}(\pmb {v}^0))\in \mathcal {P}$.

Recall that the optimal value to the equity-neutral formulation Eq. 2, denoted as $\overline{V}$, represents the maximum number of vacation requests that can be satisfied. In Proposition 2, we show there exists a Pareto-optimal solution $(\pmb {z}^{l},\pmb {x}^{l},\pmb {v}^{l}) \in \mathcal {F}_{p}$ to Eq. 4b with $f_{1}(\pmb {v}^{l})=\overline{V}$.

#### Proposition 2

Let $\overline{V}$ be the optimal objective value of problem Eq. 2. An optimal solution $(\pmb {z}^{l},\pmb {x}^{l},\pmb {v}^{l})$ to the optimization problem

7 $$\begin{aligned} \underset{\pmb {z},\pmb {v},\pmb {x}}{\mathop {\mathrm {\text {minimize}}}\limits }\ \Big \{f_{2}(\pmb {v})\Big |(\pmb {z},\pmb {v},\pmb {x})\in (1b)-(1m),f_{1}(\pmb {v})=\overline{V}\Big \} , \end{aligned}$$

is a Pareto-optimal solution to problem Eq. 4a, i.e., $(\pmb {z}^{l},\pmb {x}^{l},\pmb {v}^{l})\in \mathcal {F}_{p}$ and $(f_{1}(\pmb {v}^l),f_{2}(\pmb {v}^l))\in \mathcal {P}$.

#### Remark 1

Propositions 1 and 2 indicate that any non-dominated point $(f_{1}(\pmb {v}), f_{2}(\pmb {v})) \in \mathcal {P}$ associated with non-dominated solution $(\pmb {z},\pmb {x},\pmb {v})\in \mathcal {F}_{p}$ satisfies $f_{1}(\pmb {v})\in [f_{1}(\pmb {v}^{0}),f_{1}(\pmb {v}^{l})]_{\mathbb {Z}}$ and $f_{2}(\pmb {v})\in [0,f_{2}(\pmb {v}^{l})]$. Note also that the difference in the total number of satisfied requests between two non-dominated vacation schedules $\pmb {v}\in \mathcal {F}_p$ and $\pmb {v}'\in \mathcal {F}_p$ is at least one, i.e., $|f_1(\pmb {v})-f_1(\pmb {v}')| \ge 1$. Hence, given a non-dominated rotation schedule with a vacation schedule $\pmb {v}$ and *V* satisfied requests, the subsequent non-dominated rotation schedule might retain the same satisfied requests as in $\pmb {v}$ while fulfilling an additional request (totaling $V+1$ satisfied requests).

The results in Prepositions 1–2 and related observations in Remark 1 motivate our Pareto Search Algorithm. Algorithm 2 summarizes the steps of this algorithm. We initialize the algorithm with empty sets $\mathcal {P}$ and $\mathcal {F}_p$. In Step 1, we identify a Pareto-optimal solution and the corresponding non-dominated point with $f_2=0$. Specifically, we solve problem Eq. 6 and record optimal value $f_{1}(\pmb {v}^{0})$ and solution $S^0=\{ (\pmb {z}^{0},\pmb {x}^{0},\pmb {v}^{0})\}$ with $f_{2}(\pmb {v}^{0})=0$. It follows from Proposition 1 that $(\pmb {z}^{0},\pmb {x}^{0},\pmb {v}^{0})\in \mathcal {F}_{p}$ and $(f_{1}(\pmb {v}^0),f_{2}(\pmb {v}^0))\in \mathcal {P}$. Accordingly, we enlarge the sets $\mathcal {F}_p \leftarrow \mathcal {F}_p \cup S^0$ and $\mathcal {P}\leftarrow \mathcal {P}\cup \{(f_{1}(\pmb {v}^{0}), 0)\}$. Then, we update $V=f_{1}(\pmb {v}^{0})+1$ and extract the following categories of residents from $\pmb {v}^0$: the set of residents with one or more fulfilled requests denoted as $\mathcal {V}^0$ and the set of residents with *i*$\mathcal {U}_i^0$. For any vacation schedule $\pmb {v}^j$, where $j \ge 0$, the sets $\mathcal {V}^j$ and $\mathcal {U}_i^j$ are defined as follows.

8 $$\begin{aligned} \mathcal {V}^j&=\bigg \{r\in R,d\in D, w\in W^{\text {vac}}_{r}: v_{r,d,w}^j=1\bigg \}.\end{aligned}$$

9 $$\begin{aligned} \mathcal {U}_{i}^j&=\bigg \{r\in R:\sum _{d\in D}\sum _{w\in W^{\text {vac}}_{r}}v_{r,d,w}^j=i\bigg \}, \quad \forall i\in \big [0,\bar{w}\big ]. \end{aligned}$$

**Algorithm 2:**
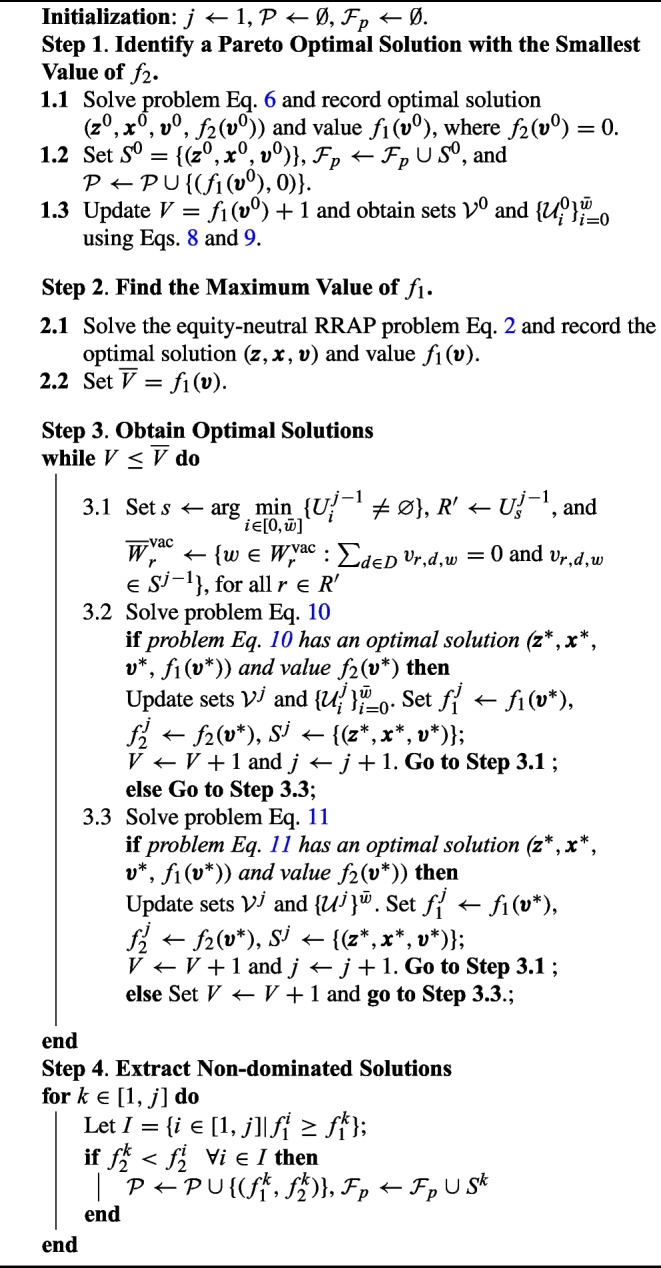
The Pareto Search Algorithm

Parameter $\bar{w}$ in Eq. 9 is the maximum number of requested weeks for vacation among all residents, i.e., $\bar{w}=\max _{r\in R}|W^{\text {vac}}_{r}|$. We use the sets $S^0$, $\mathcal {V}^0$, and $\{\mathcal {U}_{i}^{0}\}_{i=0}^{\bar{w}}$ as initial inputs to Step 3 (more on this below). In Step 2, we solve the equity-neutral model Eq. 2, record an optimal solution ($\pmb {z}, \pmb {x}, \pmb {v}$) and value $f_1(\pmb {v})$ (i.e., the maximum number of vacation requests that can be satisfied), and set $\overline{V}=f_1(\pmb {v})$. Recall that the value of $f_1(\pmb {v})$ for any non-dominated solution $(\pmb {z},\pmb {x},\pmb {v})\in \mathcal {F}_{p}$ satisfies $f_{1}(\pmb {v})\in [f_{1}(\pmb {v}^{0}),\overline{V}]_{\mathbb {Z}}$; see Remark 1. The goal of Step 3 is to identify all non-dominated rotation and vacation schedules with $f_1 \in [f_{1}(\pmb {v}^{0})+1, \overline{V}]_{\mathbb {Z}}$. Specifically, for each $V \in [f_1(\pmb {v}^{0})+1, \overline{V}]_{\mathbb {Z}}$, Step 3 (with sets $\mathcal {V}^0$, $\{\mathcal {U}_{i}^{0}\}_{i=0}^{\bar{w}}$, and $S^0=\{ (\pmb {z}^{0},\pmb {x}^{0},\pmb {v}^{0})\}$ as initial inputs) proceeds as follows. At each iteration *j*, we have $S^{j-1}$, $\mathcal {V}^{j-1}$ and $\{\mathcal {U}_{i}^{j-1}\}_{i=0}^{\bar{w}}$. In Step 3.1, we identify the set of residents with the lowest number of satisfied vacation requests from $\pmb {v}\in S^{j-1}$, denoted as $R'$, and the set of unsatisfied vacation requests $\overline{W}^{\text {vac}}_{r}$ for each $r \in R'$. Mathematically, we set $R'\leftarrow U_{s}^{j-1}$, where $s \leftarrow \arg \min \limits _{i\in [0,\bar{w}]}\{U_{i}^{j-1}\ne \varnothing \}$, and for each $r \in R'$, we set $\overline{W}^{\text {vac}}_{r}\leftarrow \{w\in W^{\text {vac}}_{r}: \sum _{d\in D}v_{r,d,w}=0 \text { and }v_{r,d,w}\in \mathcal {S}^{j-1}\}$. In Step 3.2, we attempt to find an optimal schedule with one more satisfied vacation request fulfilled for one of the residents $r \in R'$ than the vacation schedule obtained from the previous iteration, $\pmb {v}\in S^{j-1}$, while keeping the fulfilled requests for residents $r \in \mathcal {V}^{j-1}$ the same as in $\pmb {v}\in S^{j-1}$. Specifically, we solve the following problem.

10a $$\begin{aligned} \mathop {\mathrm {\text {minimize}}}\limits _{\pmb {z},\pmb {v},\pmb {x}}&\quad f_2(\pmb {v})\end{aligned}$$

10b $$\begin{aligned} \text {subject to:}&\quad (1b)-(1m), \end{aligned}$$

10c $$\begin{aligned}&\quad f_1(\pmb {v})=V ,\end{aligned}$$

10d $$\begin{aligned}&\quad v_{r,d,w}=1,\quad \forall (r,d,w)\in \mathcal {V}^{j-1},\end{aligned}$$

10e $$\begin{aligned}&\quad \sum _{r\in R'}\sum _{d\in D}\sum _{w\in \overline{W}^{\text {vac}}_{r}}v_{r,d,w}=1. \end{aligned}$$

**Fig. 2:**
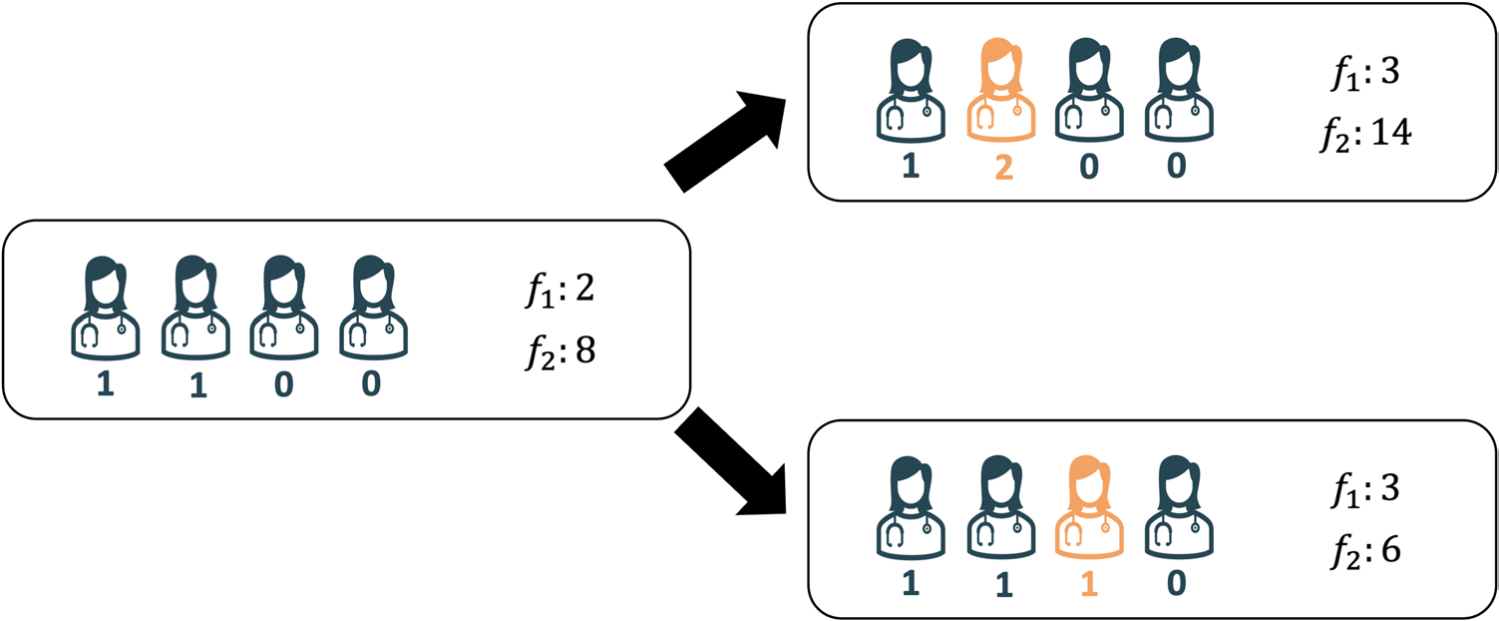
An illustration of Proposition 3 using an instance of four residents and the Gini deviation as the inequity measure $f_2$. The left panel shows a vacation schedule in which two residents have one fulfilled request while the other two have no fulfilled requests, so $f_{1}=2$ and $f_{2}=8$ in this schedule. Suppose we can satisfy one more request. The right panel shows two options. The first option is to fulfill a vacation request of one of the two residents who already have one satisfied request. In this case, $f_{1}=3$ and $f_{2}=14$. The second option is to fulfill a vacation request for one of the residents with zero satisfied requests (i.e., with the fewest satisfied requests). In this case, $f_{1}=3$ and $f_{2}=6$. Thus, the second option results in the lowest value of the inequity measure $f_2$

Problem Eq. 10a aims to find an optimal rotation schedule with $f_1=V$ satisfied vacation requests that minimize a measure of inequity in the satisfied vacation requests ($f_2=\phi (\pmb {v})$). The associated vacation schedule has the same satisfied vacation requests as the one obtained from ($j-1$), with one additional vacation request being satisfied. This feature, which is desirable since, for the purpose of comparison, it is preferred to have solutions throughout the Pareto front that involve common assignments rather than very distinct ones, is ensured via constraints Eqs. 10c–10e. Specifically, constraints Eq. 10c ensure the number of satisfied vacation requests is *V*. Constraints Eq. 10d ensure that satisfied vacation requests for residents $r \in \mathcal {V}^{j-1}$ in the schedule obtained in iteration $(j-1)$ remain satisfied in the new schedule. Constraints Eq. 10e guarantee that we prioritize satisfying an additional vacation request of one of the residents $r \in R'$ with the fewest granted vacation requests in the previous schedule obtained in iteration $j-1$ (identified in Step 3.1). Proposition 3 establishes that fulfilling a vacation request for one of the residents with the fewest satisfied requests is always optimal; see Appendix E-Companion Section B.3 for a proof and Fig. 2 for an illustrative example.

#### Proposition 3

Consider a vacation schedule $\pmb {v}$ with $V-1$ fulfilled vacation requests. Let $\mathcal {V}$ be the set of residents with at least one fulfilled request in $\pmb {v}$, as defined by Eq. 8. Let $R'$ be the set of residents with the fewest number of fulfilled requests in $\pmb {v}$ and $\overline{W}^{\text {vac}}_{r}= \{w \in W^{\text {vac}}_{r}: \sum _{d\in D}v_{r,d,w}=0\}$ be the set of unsatisfied vacation requests for each $r \in R$. Suppose we can fulfill one additional vacation request. Consider a new vacation schedule $\pmb {v}'$ with *V* satisfied requests, where we grant a vacation request for one of the residents $r \in R'$ while keeping the satisfied vacation requests for residents $r \in \mathcal {V}$ the same as in $\pmb {v}$, i.e., $v'_{r,d,w}=v_{r,d,w}=1$ for all $(r,d,w) \in \mathcal {V}$, $\sum _{r\in R'}\sum _{d\in D}\sum _{w\in \overline{W}^{\text {vac}}_{r}}v'_{r,d,w}=1$, and $f_1(\pmb {v}')=V$. Consider another vacation schedule $\pmb {v}''$ with *V* satisfied requests, where $v''_{r,d,w}=v_{r,d,w}=1$ for all $(r,d,w) \in \mathcal {V}$ and $f_1(\pmb {v}'')=V$. The following assertion holds: $f_{2}(\pmb {v}')\le f_{2}(\pmb {v}'')$.

If problem Eq. 10a has an optimal solution, we store the optimal solution and value, update sets $\mathcal {V}^j$ and $\mathcal {U}^{j}$ using Eqs. 8 and 9, set $V \leftarrow V+1$ and $j \leftarrow j+1$, and return to Step 3.1. If problem Eq. 10a does not have a feasible solution, this indicates that we cannot satisfy an additional vacation request based on the current vacation schedule $\{v_{r,d,w}=1, \ \forall (r,d,w) \in \mathcal {V}^{j-1}\}$. In this case, we proceed to Step 3.3, where we attempt to solve the following problem

11 $$\begin{aligned} \underset{\pmb {z},\pmb {v},\pmb {x}}{\mathop {\mathrm {\text {minimize}}}\limits }\ \Big \{f_{2}(\pmb {v})\Big |(\pmb {z},\pmb {v},\pmb {x})\in \{(1b)-(1m)\},f_{1}(\pmb {v})=V\Big \}. \end{aligned}$$

If problem Eq. 11 has an optimal solution, we record the optimal solution and value, update sets $\mathcal {V}^j$ and $\mathcal {U}^{j}$, set $V \leftarrow V+1$ and $j \leftarrow j+1$, and return to Step 3.1. On the other hand, if problem Eq. 11 does not have a feasible solution, this indicates that there is no optimal rotation schedule with exactly *V* satisfied vacation requests. In this case, we increase *V* by 1 and try to solve problem Eq. 11 again. Step 3 terminates when $V=\overline{V}$. Note that Step 3 generates all non-dominated solutions and some dominated solutions. In Step 4, we extract non-dominated solutions. Given that *V* is finite, then Step 3 and hence Algorithm 2 terminates in a finite number of iterations.

Solving problem Eq. 10a in Step 3.2 of Algorithm 2 is much easier than solving problem Eq. 11 in Step 3.3. Both problems aim to find a new rotation schedule with one more satisfied vacation request than the one found in the previous iteration while minimizing the value of the inequity measure, i.e., $f_2=\phi$. However, the search space in Eq. 10a is smaller because we fix $\{v_{r,d,w}=1, \ \forall (r,d,w) \in \mathcal {V}^{j-1}\}$ via constraint Eq. 10d and approve a request of one of the residents with the fewest approved requests via constraint Eq. 10e. Note also if we solve problem Eqs. 10a and 11 with the same value $f_1(\pmb {v})=V$, the resulting optimal solutions will yield the same value of the inequity measure Mathematically, consider any iteration $j \ge 1$ of Step 3 with inputs $V=f_{1}(\pmb {v}^{j-1})+1$,$\mathcal {V}^{j-1}$ and $\mathcal {U}^{j-1}$. Suppose that problem Eq. 10a with $f_1(\pmb {v})=V$ and $\mathcal {V}^{j-1}$ has an optimal solution $\pmb {v}'$ with an optimal value $f_2(\pmb {v}')$. Suppose that problem Eq. 11 with $f_1(\pmb {v})=V$ has an optimal solution $\pmb {v}^*$ with an optimal value $f_2(\pmb {v}^*)$. We have $f_2(\pmb {v}^*)=f_2(\pmb {v}')$; See Proposition 4 in Appendix E-Companion Section B.4 for a proof. Similarly, solving problem Eq. 10a is also much easier than solving problem Eq. 5 in each iteration of Algorithm 1.

### Symmetry breaking constraints

Recall that variable $z_{r,d,b}$ equals one if a resident is assigned to department *d* in block *b*. Consider residents staying in the program for the whole academic year, i.e., $\{r\in R|B^{\text {imp}}_{r}=\varnothing \}$. Filling the block schedule of these residents in arbitrary order (e.g., assigning a department to block $b+1$ before block *b*) or sequentially (e.g., assigning a department to block *b* before block $b+1$) produces equivalent solutions. The existence of such equivalent solutions leads to wasteful duplication of computational efforts in algorithms such as branch-and-bound and branch-and-cut. To avoid exploring equivalent solutions, in our implementation, we add the following symmetry-breaking constraints Eq. 12 to ensure that, for residents staying in the program for the whole academic year (i.e., $\{r\in R|B^{\text {imp}}_{r}=\varnothing \}$), their block schedules are filled sequentially (i.e., a department should be assigned to block *b* before block $b+1$):

12 $$\begin{aligned} \sum _{d\in D}z_{r,d,b}\ge \sum _{d\in D}z_{r,d,b+1},\quad \forall r\in R:B^{\text {imp}}_{r}=\varnothing , b\in B. \end{aligned}$$

## The RRAP tool

**Fig. 3:**
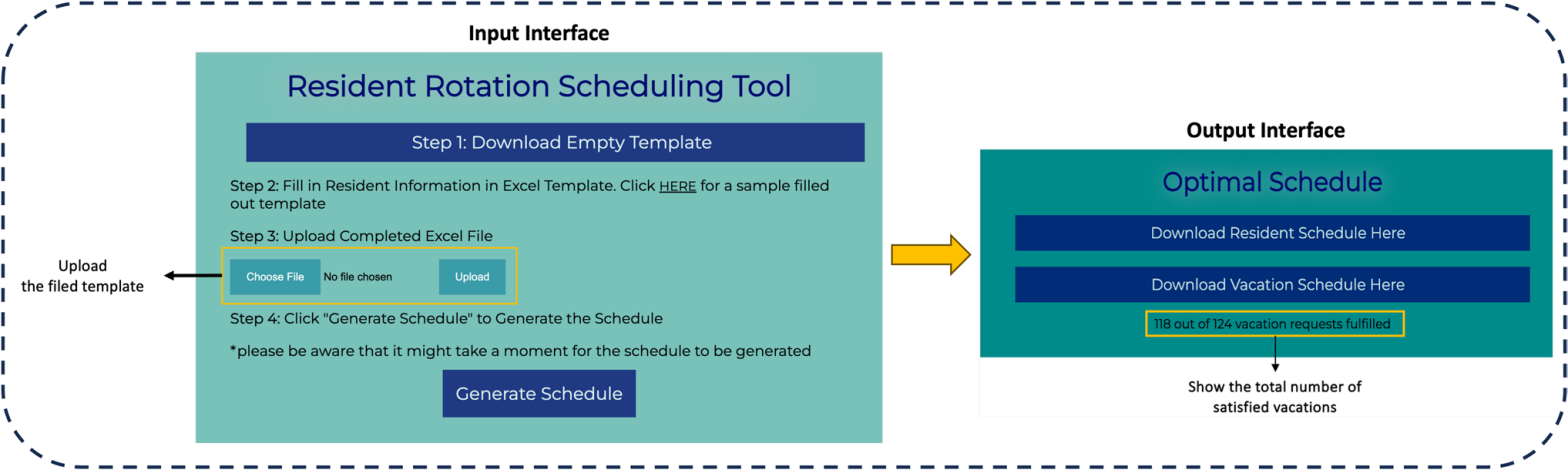
User Interface of the RRAP Tool

We developed a web-based tool implementing the proposed models using the Python language to automate the generation of the annual rotation schedule. Figure 3 presents a screenshot of the user interface, which has two modules: the input interface and the output interface. Details of these components are provided next.

The input interface is used to input data (i.e., details of an RRAP instance) required for solving the IP model. Specifically, the user first downloads the info template (Step 1), an Excel workbook that has several sheets, each designated for a specific set of input parameters to the IP model (e.g., program information, block information, list of residents, hospital departments, mandatory and impossible departments, etc.); see Appendix E-Companion Section C for an example. Once the user fills out the info template, they upload it using the upload function (Step 3). Then, they click on the “Generate Schedule” function to generate the rotation schedule. Specifically, in the back end, this function reads the data from the filled input template, uses it as input to the IP model, and then calls Gurobi to solve the model. The output interface enables users to download the optimal solution using two Excel workbooks. The first provides the rotation schedule, and the second provides the associated vacation schedule.

We worked closely with the collaborating residency program to ensure the tool is user-friendly and implementable in practice. This included multiple feedback sessions and iterative design improvements based on user input. We also conducted hands-on training sessions to facilitate adoption and ensure ease of use. These efforts enhanced the end-users’ engagement and reinforced trust in the tool’s reliability. The tool is available upon request from the authors.

## Numerical results

In this section, we construct various RRAP instances and conduct extensive experiments to illustrate the computational efficiency of the proposed model; compare optimal solutions to the proposed models and their impact on equity; investigate the trade-off between equity in satisfying vacation requests and the number of satisfied requests; and derive insights relevant to practice. In Section 7.1, we discuss experimental setups. In Section 7.2, we analyze the trade-off between inequity and the number of satisfied requests under different inequity measures. In Section 7.3, we compare our proposed approach with the manual approach. In Section 7.4, we compare the residents-based and class-based approaches. Finally, in Section 7.5, we analyze the computational performance of our approaches and the $\varepsilon$-constraint method.

### Test instances and experimental setup

To show our proposed approach’s broad applicability and benefits, we constructed various RRAP instances based on the data provided by CUIMC’s general surgery residency program. As detailed in Section 3, this program is a five-year-long program, where each academic year consists of $|W|=53$ weeks. We constructed three sets of RRAP instances based on the data from six academic years (2018–2023) by varying the number of blocks and departments for each resident class.

Table 4 summarizes the number of blocks and departments for each class in each instance. The number of weeks in each block in each instance is as follows. For Inst1, the number of weeks in each block of (PGY1, PGY2, PGY3, PGY4, PGY5) is (4 to 5, 6 to 7, 6 to 7, 7 to 9, 9 to 10). For Inst2, the number of weeks in each block of PGY1–PGY5 is 4 to 5 weeks. For Inst3, the number of weeks in each block of (PGY1, PGY2, PGY3, PGY4, PGY5) is (4 to 5, 4 to 5, 5 to 6, 5 to 6, 5 to 6). Table 5 summarizes the sets of mandatory/required (i.e., $D^{\text {req}}_{}$) and busy departments (i.e., $D^{\text {busy}}{}$), where busy departments are highlighted in bold text. The set of impossible departments includes those that are not mandatory, i.e., $D^{\text {imp}}_{r}=D\setminus D^{\text {req}}_{r},\forall r\in R$. Note that Inst2 represents a residency program where all resident classes have the same sets of blocks and mandatory departments. In contrast, Inst1 and Inst3 represent programs where resident classes have different rotation requirements. We provide details of departmental staffing needs ($R^{{\tiny min}}_{}$ and $R^{{\tiny max}}$) and the required rotation length ($T^{{\tiny min}}_{}$ and $T^{{\tiny max}}_{}$) in Appendix E-Companion Section D.

**Table 4:** Number of blocks and possible departments for each class in each RRAP Instances

Inst	Blocks						Departments				
	PGY1	PGY2	PGY3	PGY4	PGY5		PGY1	PGY2	PGY3	PGY4	PGY5
Inst1	12	8	9	7	6		12	6	8	7	6
Inst2	12	12	12	12	12		12	12	12	12	12
Inst3	12	12	10	10	9		12	10	10	10	9

**Table 5:** Mandatory and busy departments for each class in each RRAP Instance. Busy departments are highlighted in bold text

Inst	Class	Departments
Inst1	PGY1	Allen, Vascular, Breast, Thoracic, CR, SICU, HPB, Peds, Overlook, Lap, **Rainbow**, **VTF**
	PGY2	Overlook, ACS-OR, Consults, CTICU, Peds, Allen
	PGY3	Renal, Lap, CR, Trauma, Overlook, Vascular, Breast, Thoracic
	PGY4	Overlook, Elective, Vascular, HPB, ACS, Allen, **Nights**
	PGY5	HPB-Chabot, CR, HPB, Lap, Elective,**Nights**
Inst2	PGY1	Allen, Vascular, Breast, Thoracic, CR, SICU, HPB, Peds, Overlook, Lap, **Rainbow**, **VTF**
	PGY2	Allen, Vascular, Breast, Thoracic, CR, SICU, HPB, Peds, Overlook, Lap, **Rainbow**, **VTF**
	PGY3	Allen, Vascular, Breast, Thoracic, CR, SICU, HPB, Peds, Overlook, Lap, **Rainbow**, **VTF**
	PGY4	Allen, Vascular, Breast, Thoracic, CR, SICU, HPB, Peds, Overlook, Lap, **Rainbow**, **VTF**
	PGY5	Allen, Vascular, Breast, Thoracic, CR, SICU, HPB, Peds, Overlook, Lap, **Rainbow**, **VTF**
Inst3	PGY1	Allen, Vascular, Breast, Thoracic, CR, SICU, HPB, Peds, Overlook, Lap, **VTF**, **Rainbow**
	PGY2	Allen, Vascular, Breast, VTF, Thoracic, CR, HPB, Consults, **ACS**, **Overlook**
	PGY3	Allen, Vascular, Breast, VTF, Thoracic, CR, SICU, HPB, Trauma, **Overlook**
	PGY4	Allen, Vascular, Breast, Lap, Thoracic, CR, SICU, HPB, **Overlook**, **Nights**
	PGY5	Allen, Vascular, Breast, Lap, Thoracic, CR, SICU, ACS, **Nights**

Allen: Allen Hospital; CR: Colorectal Surgery; SICU: Surgical Intensive Care Unit; HPB: Hepatopancreaticobiliary; Peds: Pediatric Surgery; Rainbow: Night Float for PGY1; Lap: Advanced Laparoscopic Surgery; Overlook: Overlook Hospital; ACS-OR: Acute Care Surgery Operative; Consults: Acute Care Surgery Consult; CTICU: Cardiothoracic Intensive Care Unit; Renal: Renal Transplant; Nights: Night Float; VTF: Veterinary Treatment Facility

We generate vacation requests for each resident using two methods, denoted as A and B. (One can employ any other method to generate vacation requests.) Method A simulates real-world vacation request patterns, where each resident requests a set of non-overlapping weeks in the academic year. Specifically, in method A, we randomly select vacreq non-overlapping weeks from the 53 weeks for each resident. Method B, generates another possible vacation request distribution, where residents’ requests are proportionally distributed across the three parts of the academic year. Specifically, we first partition the academic year evenly into three parts, denoted as $W_{1}$, $W_{2}$, and $W_{3}$. For illustrative purposes, we randomly generate vacation requests as follows: 20% of residents’ vacation requests are drawn from $W_{1}$, 30% of requests are drawn from $W_{2}$, and the remaining 50% of vacation requests are from $W_{3}$. Based on these methods and Inst1–Inst3, we construct 18 RRAP instances with $|R| \in \{62,125,185\}$ residents, where instances with $|R|=125$ and 185 represent large instances as the typical number of general surgery residents at CUMIC often ranges from 50–65. We denote each instance as Inst#-R-Method. For example, Inst1-62-A is Inst1 with 62 residents and vacation requests generated using method A.

Residents are required to stay in the program for the entire academic year (i.e., $B^{\text {imp}}_{r}=\varnothing , \forall r \in R$). Moreover, ACGME requires each resident to have four vacation weeks during the academic year. Accordingly, we set $|W^{\text {vac}}_{r}|=4$ in all instances. Each resident could submit two vacation requests at the beginning of the year (i.e., $T^{\text {vac}}_{r}=2, \forall r \in R$), and each can have at most one vacation in each block/rotation (i.e., $R^{\text {vac}}_{r,b}=1, \forall r \in R, b \in B)$. Moreover, for instances with 62 and 125 residents, at most, one resident can be on vacation per week in each department, i.e., $D^{\text {vac}}_{d,w}=1$, for all $d \in D$ and $w \in W$. For instances with 185 residents, at most three residents can be on vacation per week in each department, i.e., $D^{\text {vac}}_{d,w}=3$, for all $d \in D$ and $w \in W$.

We use the constructed 18 RRAP instances (Inst1-62-A, Inst1-62-B,..., Inst3-185-B) to analyze the computational time of the proposed methodologies in Section 7.5. In addition, we use a case study based on the data related to the 2023–2024 academic year to derive the practical insights discussed in Sections 7.2–7.4. We call this instance RRAP-Case. Specifically, this instance consists of 54 residents, of which (22, 9, 9, 7, 7) are (PGY1, PGY2, PGY3, PGY4, and PGY5) residents. We consider five types of PGY1 residents in this instance^1^, namely PGY1-Categorical, PGY1-OMFS, PGY1-Prelim, PGY1-GU, and PGY1-Ortho. PGY1-Categorical and PGY1-Prelim are general surgery residents with categorical and preliminary positions, respectively (see Section 3 for details). On the other hand, PGY1-GU, PGY1-OMFS, and PGY1-Ortho are from the urology, oral and maxillofacial, and orthopedic surgical residency programs, respectively, rotating within the general surgery program. PGY2 and PGY3 residents are either Categorical or Cardiac. All residents, except PGY1-GU and PGY1-Ortho, rotate in the program for the entire academic year, while PGY1-GU and PGY1-Ortho spend 40 and 26 weeks, respectively. Moreover, each resident, except PGY1-Ortho, have four vacation weeks ($T^{\text {vac}}_{r}=4$), while PGY1-Ortho (because they are only present half of the year) have two vacation weeks ($T^{\text {vac}}_{r}=2$). Each resident can submit requests for $|W^{\text {vac}}_{r}|=T^{\text {vac}}_{r}$ preferred vacation weeks. The sets of blocks (mandatory, possible, and busy) departments for each class and each type of resident are different. We refer to Appendix E-Companion Section G for details and parameter settings of this instance.

We implemented our proposed models and algorithm in Python 3.8.6 and used Gurobi 9.5.0 as the solver with default settings. We conducted all the experiments on a MacBook Pro with an M1 chip and 32 gigabytes (GB) RAM. This represents the computing capability available to a chief resident who would be tasked with producing a schedule in a real-world setting.

### Analysis of the trade-off between inequity and number of satisfied requests

**Fig. 4:**
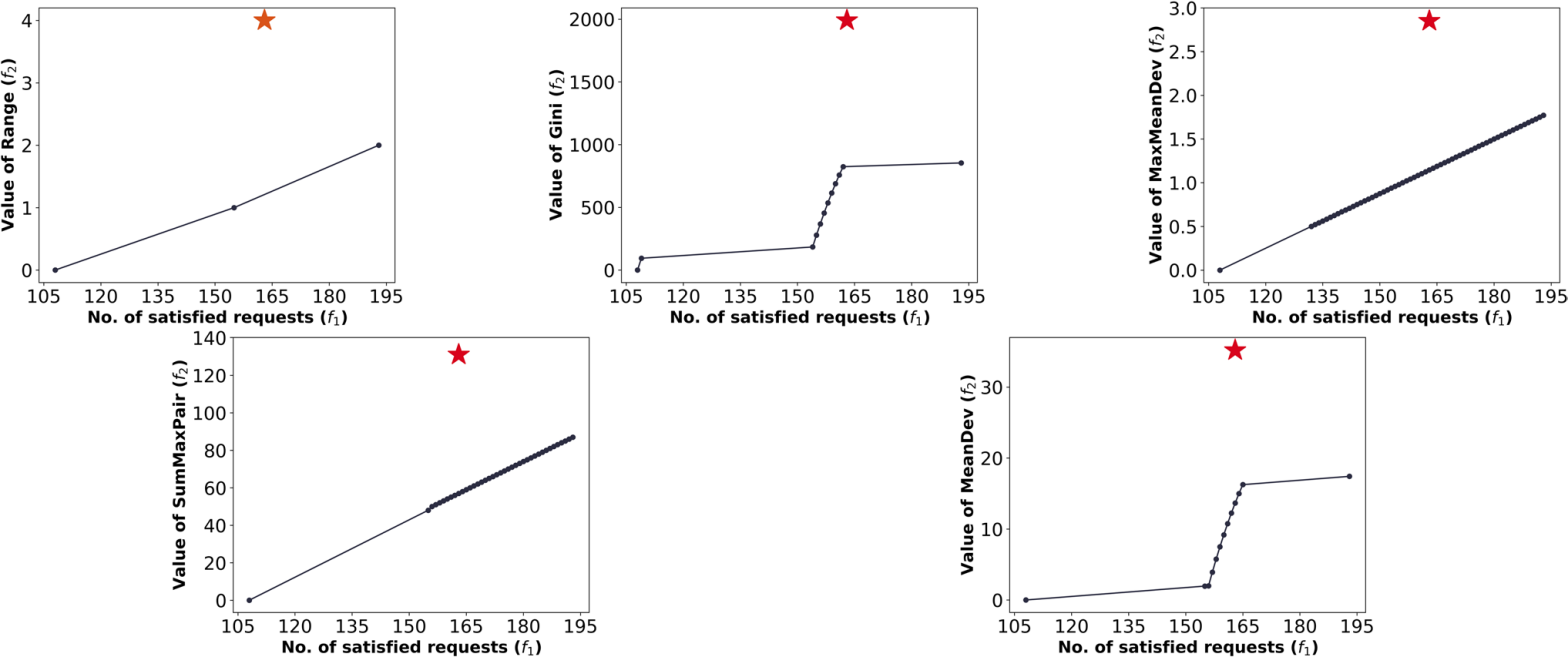
Pareto fronts illustrating the trade-off between the number of satisfied vacation requests ($f_1$) and inequity ($f_2$). The red star represents a feasible rotation schedule

In this section, we analyze the trade-off between inequity ($f_2$) and the number of satisfied vacation requests ($f_1$) using the RRAP-Case instance. Figure 4 presents the Pareto fronts (trade-off curves) obtained by solving this instance using the Pareto Search Algorithm with different inequity measures. The (*x*, *y*) values of each point on the curve are the value of ($f_1$, $f_2$)=(total number of satisfied requests, value of the inequity measure) associated with a Pareto optimal rotation schedule. The red star represents the values of ($f_1$, $f_2$) associated with a feasible rotation schedule obtained by solving the feasibility problem Eq. 1a.

Figure 4 provides several important insights. First, all non-dominated points lie between [108, 0] and $[193, f_{2}]$, where 108 is the optimal value of problem Eq. 6, $f_{2}$ is the optimal value of problem Eq. 7, and $f_{1}$=193 is the optimal value of the equity-neutral problem Eq. 2. This is consistent with our theoretical results in Propositions 1–2. Second, the equity-neutral model produces rotation schedules that maximize both the number of satisfied requests and the value of each inequity measure. This shows that these schedules exhibit significant disparities in meeting residents’ vacation requests. Third, the optimal equity-promoting rotation schedules always dominate the one obtained by solving the feasibility problem Eq. 1a (represented by a star in Fig. 4). In particular, using a feasible solution, we can satisfy 163 vacation requests, and the corresponding values of (Range, Gini, MeanDev, MaxMeanDev, and SumMaxPair) are (4, 1990, 35.2, 2.9, 131). These points are notably above the Pareto fronts. Moreover, we can find non-dominated rotation schedules with more satisfied requests and lower values of each measure.

Fourth, it is clear that different choices of the inequity measure in the equity-promoting model produce different sets of Pareto optimal (non-dominated) rotation schedules with varying impacts on equity and the number of satisfied requests. Moreover, the number of non-dominated rotation schedules and points varies under different measures. Specifically, the number of non-dominated points under (Range, Gini, MeanDev, SumMaxPair, MaxMeanDev) = (3, 12, 13, 40, 63). Note also that the non-dominated points are different. For example, using the Gini deviation, we can find a rotation schedule with 109 satisfied requests and a Gini deviation of 94, i.e., $(f_{1},f_{2})=(109,94)$. But there is no such rotation schedule with 109 satisfied requests under other measures.

**Fig. 5:**
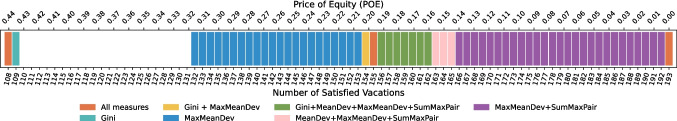
The price of equity and the number of satisfied vacations

Next, we analyze the *Price of Equity* (POE) associated with these non-dominated rotation schedules. The POE is the loss in efficiency incurred in the pursuit of equity, where efficiency in the context of the RRAP is measured by the total number of satisfied requests. We compute POE as follows. First, we obtain the maximum number of satisfied vacation requests that can be achieved by solving the equity-neutral model Eq. 2 denoted as $\overline{V}$. Then, we compute the POE associated with the non-dominated solution as

13 $$\begin{aligned} \text {POE}= \frac{f_{1}(\pmb {v})-\overline{V}}{\overline{V}}, \end{aligned}$$

where $f_{1}(\pmb {v})$ is the value of $f_1$ (i.e., number of satisfied requests) associated with each non-dominated vacation schedule $\pmb {v}$ presented in Fig. 4. The value of POE is often between 0 and 1, measuring how close the efficiency is of the non-dominated equity-promoting solutions with respect to the equity-neutral solutions. When POE is equal to 0, this indicates that the equity-promoting solutions are also efficient. In contrast, a POE $>0$ indicates that the equity-promoting solutions are less efficient than the equity-neutral solutions. Figure 5 presents the POE associated with the Pareto optimal solutions to the equity-promoting models. Points marked with the same color are those identified by the same subset of inequity measures.

We observe the following from Fig. 5. The value of POE ranges from 0 to 0.44. Schedules with a POE $\text =0.44$ (POE $\text =0$) are the most equitable (efficient). Using all measures, we can find non-dominated rotation schedules with ($f_1$, POE)=(108, 0.44), (155, 0.197), and ($\overline{V}=193$, 0). The three non-dominated rotation schedules obtained using the Range as the inequity measure have the same ($f_1$, POE) values as those schedules. Hence, this measure does not provide the decision-maker with various rotation schedules to explore or offer holistic insight into the trade-offs between the two objectives.

Notably, using the MaxMeanDev as the inequity measure, we obtain the largest set of non-dominated rotation schedules (63 schedules). Some of these have POE values (and hence the same impact on equity and efficiency) equal to those associated with non-dominated rotation schedules identified under other measures. Specifically, employing the MaxMeanDev as the inequity measure, we identify 61 non-dominated rotation schedules with $f_1 [132, 192]$ and POE $\in [0.01,0.32]$ in addition to the two extremes (i.e., those with $f_1=\{108, 193\}$ and POE $\in {0, 0.44}$). There are no rotation schedules with $f_1 \in [132,153]$ and POE $\in [0.21, 0.32]$ under the other measures. Using Gini, MeanDev, MaxMeanDev and SumMaxPair, we can find rotation schedules with $f_1 \in [156,162]$ and POE $\in [0.16, 0.19]$. Using SumMaxPair, MeanDev and MaxMeanDev, we can additionally identify rotation schedules with higher $f_1$ values (163, 164, 165) and lower POE values (0.155, 0.15, 0.145), indicating that these schedules are more efficient (less equitable). Furthermore, MaxMeanDev and SumMaxPair are the only two measures that can identify rotation schedules with $f_1 [166,192]$ and POE $\in {} [0.01, 0.14]$. Finally, we observe that using the Gini deviation, we can find a rotation schedule with $f_1=109$ and POE $\text {}=0.435$ (the second largest value of POE). However, there is no such rotation schedule with 109 satisfied requests under other measures. Moreover, the Gini deviation and MaxMeanDev are the only measures that generate rotation schedules with $f_1=154$.

We close this section by noting that while there is no clear winner among these inequity measures or criteria for selecting any of them, the program director and chief resident of the collaborating health system favor utilizing measures that identify a larger number of non-dominated solutions (e.g., MaxMeanDev and SumMaxPair). This choice allows for more rotation scheduling options and flexibility in selecting a preferred schedule while acknowledging the trade-offs between the two objectives.

### Comparison with the sequential approach

In practice, the chief resident or program director employs a sequential approach, separating rotation assignment decisions ($\pmb {z}$) from the remaining decisions ($\pmb {v}$ and $\pmb {x}$). Specifically, first, they assign residents to blocks and departments (i.e., find a feasible $\pmb {z}$). Then, they construct a vacation schedule, specifying vacation and working weeks for each resident (i.e., find feasible $\pmb {v}$ and $\pmb {x}$). In this section, we compare the performance of this sequential approach with our proposed integrated approach. In the sequential approach, we first solve a feasibility problem $\max \limits _{\pmb {z}} \{ 0| (1b)-(1e), (1g)-(1h), \pmb {z}\in \{0,1\}^{|R| \times |D| \times |B|}\}$ to obtain a feasible $\varvec{\bar{z}}$. Then, we solve the following equity-promoting IP with $\varvec{z}$ fixed to $\varvec{\bar{z}}$ to obtain corresponding ($\pmb {v}$, $\pmb {x}$) and ($f_1, f_2$).

14a $$\begin{aligned} \underset{\pmb {v,x}}{\text {maximize}}&\qquad f_{1}(\pmb {v}):=\sum _{r\in R}\sum _{d\in D}\sum _{w\in W^{\text {vac}}_{r}}v_{r,d,w} \end{aligned}$$

14b $$\begin{aligned} \underset{\pmb {v,x,z}}{\text {minimize}}&\qquad f_{2}(\pmb {v}):=\phi (\pmb {v}) \end{aligned}$$

14c $$\begin{aligned} \text {subject~to:}&\qquad (\pmb {v}, \pmb {x}) \in \{(1f),(1i)-(1m)\} \end{aligned}$$

The performance of the sequential approach depends on rotation assignment decisions ($\varvec{\bar{z}}$), i.e., input to problem Eq. 14. Hence, for a fair comparison, we implemented the sequential approach with three different rotation assignment decisions $\varvec{\bar{z}}_1$, $\varvec{\bar{z}}_2$, $\varvec{\bar{z}}_3$. These solutions were obtained by solving the feasibility problem five times. After each solve, we added constraints that exclude all previously identified feasible solutions. From the resulting set of solutions, we selected the three most distinct ones (i.e., $\varvec{\bar{z}}_1$, $\varvec{\bar{z}}_2$, and $\varvec{\bar{z}}_3$).

**Fig. 6:**
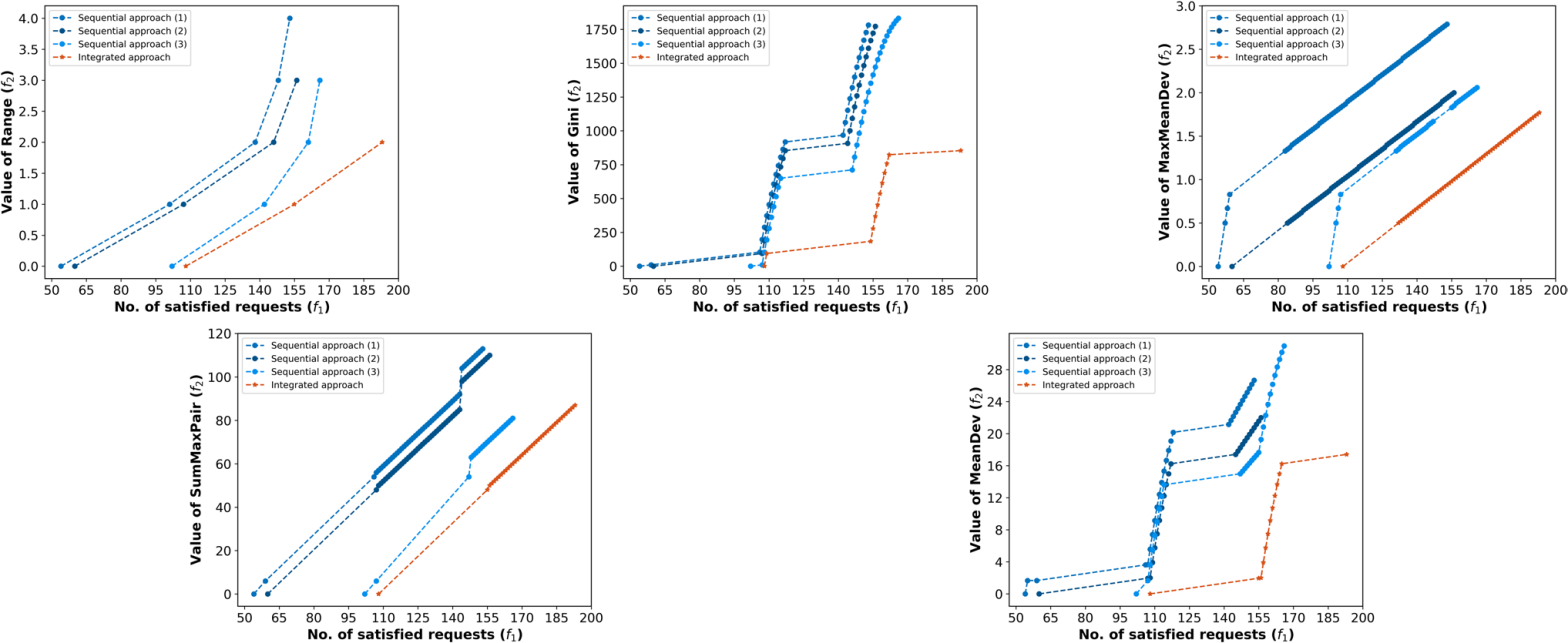
Pareto fronts resulting from the integrated and sequential approaches

Figure 6 presents the Pareto fronts obtained by solving the RRAP-Case instance using our integrated approach and the sequential approach. We denote Pareto fronts corresponding to $\varvec{\bar{z}}_1$, $\varvec{\bar{z}}_2$, $\varvec{\bar{z}}_3$ as sequential approach (1), (2) and (3), respectively. We observe that the sequential approach consistently results in inferior solutions, which are dominated by those obtained using our proposed approach. In particular, our approach allows us to find equitable rotation schedules with a larger number of satisfied vacation requests and a lower value of the inequity measure. These results demonstrate the importance of integrating rotation and vacation scheduling decisions and show how our integrated equity-promoting approach can yield more equitable rotation schedules than the equity-promoting sequential approach, which separates rotation and vacation scheduling decisions.

### Residents-based versus class-based equity

Recall from Section 4.3 that one can employ the equity-promoting model Eq. 4a to promote equity among all residents or within residents of the same class. In the former approach (denoted as the residents-based), we evaluate inequity across the entire set of residents, and in the latter (denoted as the class-based), we evaluate inequity within each class of residents. In this section, we compare solutions obtained from these approaches.

**Fig. 7:**
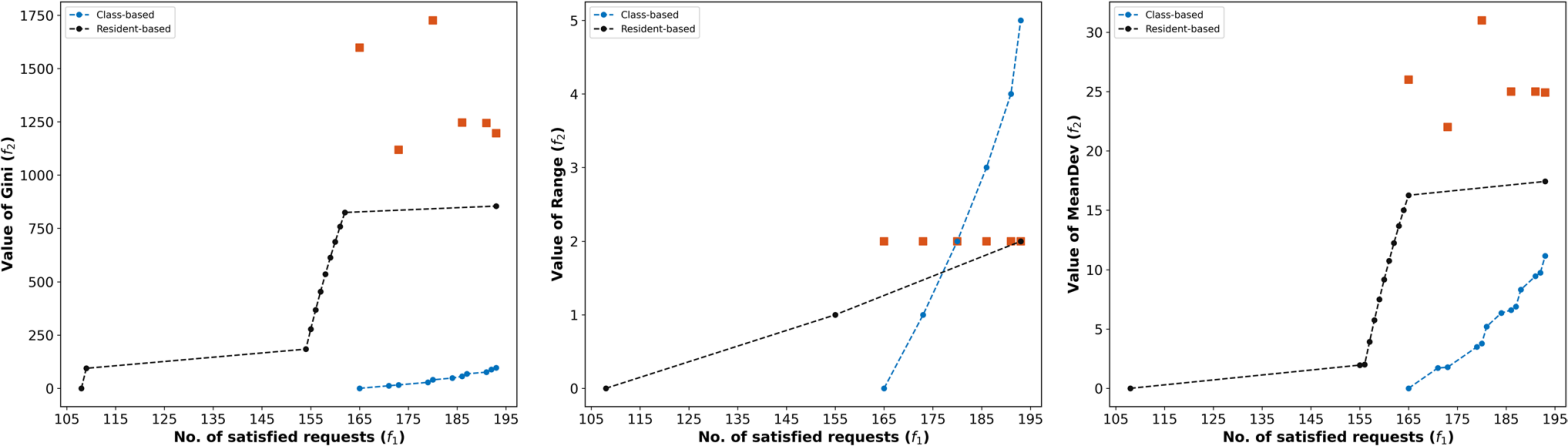
Pareto fronts resulting from residents-based and class-based approaches

For brevity and illustrative purposes, in Fig. 7, we present Pareto fronts obtained using the residents-based (black curve) and class-based (blue curve) approaches with Range, Gini, and MeanDev. The $f_2$ value of each point on the class-based curve is computed as $f_{2}(\pmb {v})= \sum _{c \in C} \phi _c(\pmb {v})$, where *C* is the set of resident classes, i.e., $C=\{\text {PGY1}, \text {PGY2}, \text {PGY3}, \text {PGY4}, \text {PGY5}\}$, and $\phi _c(\pmb {v})$ the value of the inequity measure for each class. We also evaluate the impact of employing class-based schedules on inequity among all residents by computing the value of the inequity measure considering the entire set of residents as in the residents-based approach. Red squares in Fig. 7 represent the resulting ($f_1$, $f_2$) values.

It is clear that the residents- and the class-based approaches result in distinct sets of rotation schedules. This makes sense because they consider different sets of residents when measuring inequity. Moreover, while the class-based approach improves equity within each class, it potentially results in significant disparities among residents of different classes compared with the residents-based approach. In particular, rotation schedules generated using the class-based approach result in high values of each inequity measure when computed considering all residents. These values are significantly higher than those associated with rotation schedules obtained using the residents-based approach (see red squares in Fig. 7).

It is worth noting that there is no universal agreement in the literature regarding the preference for either an individual-based approach (as in the residents-based) or a group-based approach (as in the class-based). The choice often depends on the context and is a matter of subjective evaluation. The results in this section indicate that, in terms of promoting equity among all residents, regardless of their class, the class-based approach may not be suitable.

### Computational performance

In this section, we analyze the solution time of the proposed approaches. We first generate five instances of each of the 18 RRAP instances (Inst1-62-A, Inst1-62-B,..., Inst3-185-B) described in Section 7.1 for a total of 90 instances and then solve each with the proposed models and the Pareto Search Algorithm.

Let us first analyze the computational performance of the Pareto Search Algorithm (Algorithm 2). Table 6 presents the total solution time (in seconds) required to generate the complete set of Pareto optimal rotation schedules to the equity-promoting model with measures Range, Gini, MeanDev, MaxMeanDev, and SumMaxPair. We do not present solution times with measures MaxPair and MaxSumPair since, as shown in the Proof of Proposition 3 in Appendix E-Companion Section B, the former measure is equivalent to Range, and the latter is equivalent to MaxMeanDev and thus have comparable solution times. In Appendix E-Companion Section E, we present the average time spent in each step of Algorithm 2.

We observe the following from Table 6. First, we can obtain the set of Pareto optimal solutions to the equity-promoting model using Algorithm 2 in less than two hours, irrespective of the inequity measure used in the model. Second, the algorithm takes a longer time as the instance size increases. Specifically, the ranges of the total time required to obtain the complete set for instances with 62, 125, and 185 residents are approximately 1.6–9 minutes, 12–62 minutes, and 15 minutes–2.2 hours. These results make sense because the size of the equity-promoting formulation and thus problems Eqs. 10a and 11 in Step 3 of the algorithm increase with |*R*|, potentially increasing the time required to solve each in each iteration of the algorithm. Moreover, instances with more residents have more vacation requests and, hence, have larger numbers of non-dominated solutions, potentially increasing the time required to identify these solutions. However, these solution times are suitable for practical implementation, considering that the rotation schedule is constructed once a year and CUIMC typically has a range of 50 to 65 general surgery residents.

**Table 6:** The total time (in seconds) required to obtain the set of non-dominated solutions using Algorithm 2

Instance	Range	Gini	MeanDev	MaxMeanDev	SumMaxPair
Inst1-62-A	140.1	239.5	235.4	174.9	218.1
Inst1-62-B	208.0	239.5	527.0	241.0	546.8
Inst2-62-A	118.6	301.2	157.2	95.5	200.2
Inst2-62-B	143.9	222.8	311.1	158.7	500.8
Inst3-62-A	110.0	239.5	183.9	139.5	177.1
Inst3-62-B	154.3	644.1	333.6	182.4	434.6
Inst1-125-A	557.8	1179.2	1188.7	853.1	1267.0
Inst1-125-B	920.1	1222.4	1265.0	1047.1	3778.2
Inst2-125-A	699.3	2874.3	1703.2	1360.6	1016.5
Inst2-125-B	1250.0	3528.7	2028.7	2586.9	1370.3
Inst3-125-A	496.4	1243.5	1020.4	877.0	953.0
Inst3-125-B	704.3	1834.5	1150.8	942.4	1589.7
Inst1-185-A	1209.5	4077.0	3344.0	2091.8	1267.0
Inst1-185-B	1637.6	4491.1	4342.1	2475.4	3825.4
Inst2-185-A	1350.3	6240.8	3252.8	7273.5	2324.0
Inst2-185-B	1772.1	5971.6	3737.1	2797.4	4005.5
Inst3-185-A	917.5	4091.1	2597.9	1882.4	2184.0
Inst3-185-B	2932.2	7972.1	7807.7	3315.1	6190.3

Third, the algorithm’s computational performance varies depending on the inequity measure employed in the equity-promoting model. In particular, the algorithm takes a shorter time to find the non-dominated set under the Range measure than under the other considered measures, and it takes the longest time under the Gini deviation measure. Intuitively, different measures require introducing different sets of additional variables and constraints into the model; see Appendix E-Companion Section A. Thus, the size and complexity of solving the resulting formulation differ under each measure. For example, we need two additional variables and $2|R|+1$ constraints to represent the Range. In contrast, we need $|R|^{2}$ variables and $2|R|^{2}$ constraints to represent the Gini deviation (see Table 7 in Appendix E-Companion Section A). Thus, the size of the equity-promoting formulation employing the Gini deviation is significantly larger. Such an increase in the size of the IP formulation often suggests an increase in solution time for solving it [37].

Fourth, we observe that Step 3 of the algorithm requires the longest time (see Appendix E-Companion Section E) as this step generates the entire set of Pareto optimal solutions by iteratively solving either problem Eq. 10a (in Step 3.2) or problem Eq. 11 (in Step 3.3). Consider Inst1-62-A, for example, the total solution time of this instance using the algorithm with the Range measure is 140 seconds, 133 of which are spent in Step 3. Finally, we observe that instances with vacation requests generated using method B often require a slightly longer time to solve than those with vacation requests generated using method A. This makes sense as method B may lead to more conflicting requests than method A and, thus, potentially harder-to-solve instances.

Note that using the traditional $\varepsilon$-constraint method (Algorithm 1), we could not solve any of the generated RRAP instances under the Gini, SumMaxPair, and MeanDev. Consider Inst1-125-A for example. Using the Pareto Search Algorithm (Algorithm 2), we can obtain all the Pareto optimal solutions (16, 15, 2) with average solution time (1179.2, 1188.7, 1267) seconds for the equity-promoting model with (Gini, MeanDev, SumMaxPair). In contrast, Algorithm 1 cannot find the entire set within a day. In fact, Algorithm 1 terminates at the third iteration with an average MIP gap of (63%, 44%, 79%) for (Gini, MeanDev, SumMaxPair).

Finally, we analyze solution time using formulation Eq. 1b and the equity-neutral model Eq. 2. In Appendix E-Companion Section F, Tables 13 and 14 respectively present solution time of formulations Eqs. 1a and 2 in Tables 13 and 14. Using the equity-neutral model Eq. 2, we can solve all instances with an average solution time ranging from 2.5 to 60 seconds. In fact, we can quickly solve even larger (though not realistic) instances of the problem using this model. For example, the average solution time of instances with 200, 400, and 600 residents ranges from 12 to 80 seconds. Similarly, using formulation Eq. 1, we can solve all the instances quickly with an average solution time ranging from 0.63 to 7.45 seconds.

The results in this section demonstrate the computational efficiency of our proposed approaches for the RRAP.

## Conclusion

Motivated by our collaboration with CUIMC, we propose and analyze new IP models and approaches for the resident-to-rotation assignment problem (RRAP). First, we derive an IP formulation that finds a feasible rotation and vacation schedule that satisfies all rotation requirements. We show that such a formulation and the corresponding equity-neutral formulation that maximizes the number of satisfied vacation requests lead to disparity in satisfying vacation requests among residents. To address this, we derive an equity-promoting counterpart, which finds optimal rotation schedules that maximize the total number of satisfied vacation requests and minimize a measure of inequity in satisfied requests among residents. Second, we propose a computationally efficient Pareto Search Algorithm that identifies the complete set of Pareto optimal (non-dominated) rotation schedules for the equity-promoting model within a time frame suitable for practical implementation.

Third, to bridge the gap between theory and practice, we developed a web-based, user-friendly tool that implements the proposed methodologies, enabling residency programs to automate their rotation schedules. Finlay, to illustrate the practical benefits of our proposed approach, we constructed various instances based on data from the CUIMC and conducted extensive experiments. Our results

- (a) demonstrate the computational efficiency and implementability of our approaches and underscores their potential to enhance fairness in resident rotation scheduling. Moreover, we show the superior computational performance of our Pareto Search Algorithm compared with the traditional $\varepsilon$-constraint method.
- (b) illustrate the trade-off between equity in satisfying vacation requests and the number of satisfied requests;
- (c) show how equity-neutral models and manual methods lead to unfair rotation schedules and disparities in satisfying vacation requests;
- (d) illustrate how different choices of the inequity measure in the equity-promoting model results in different sets of Pareto-optimal rotation schedules;
- (e) emphasize the importance of integrating rotation and vacation scheduling decisions to ensure equity among residents and show the negative consequences on fairness when adopting the existing sequential approach that separates the rotation and vacation scheduling decisions.

Ensuring fairness among residents and accounting for their vacation preferences can enhance resident satisfaction, educational experience, and performance—factors that ultimately contribute to improved quality of care and the residency program’s reputation. By streamlining a traditionally manual and error-prone task, our approach and tool promote more equitable and efficient schedule generation. Implementing our approach has strong potential for a positive societal impact in graduate medical education and healthcare workforce planning.

For future research, extending the proposed approaches by considering potential uncertain events that could affect residents’ training and rotations (e.g., residents’ absenteeism) would be valuable. It will also be interesting to consider residents’ preferences on possible shifts within each rotation and build on our proposed approach to promote equity in shift schedules. These extensions require new models and solution methodologies and thus will result in worthwhile contributions to the related literature.

## Figures and Tables

**Fig. 8:**
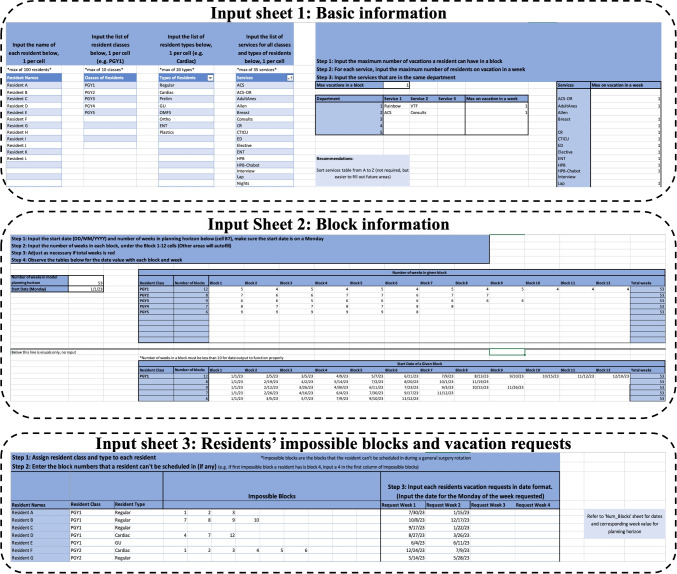
Screenshots of sheets 1–3 of the input info template of the RRAP tool

**Fig. 9:**
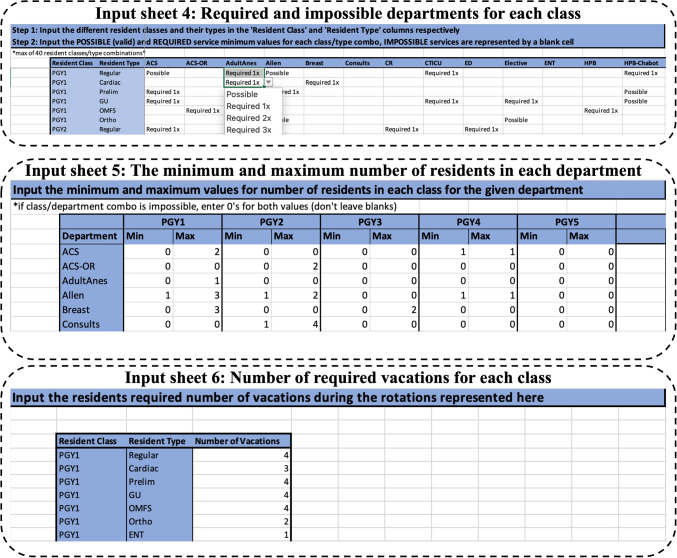
Screenshots of sheets 4–6 of the input info template of the RRAP tool

**Fig. 10:**
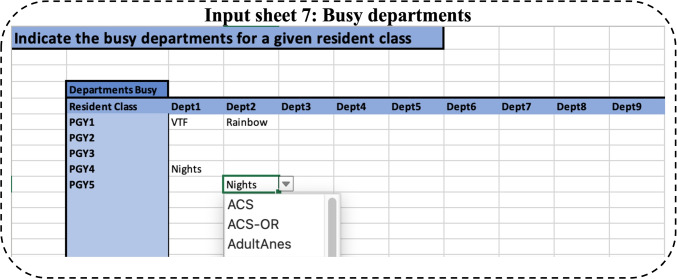
Screenshots of sheet 7 of the input info template of the RRAP tool

## Data Availability

The research did not involve human participants and was conducted using data that did not include the personal information of any resident. To demonstrate the broad applicability and benefits of our proposed approach, we constructed various RRAP instances based on the data provided by CUIMC’s general surgery residency program. Details related to these instances are provided in Section 7. The original data are not publicly available due to security and privacy restrictions.

## Equity-promoting integer programming approaches for medical resident rotation scheduling (E-Companion)

### E-Companion Section A. Equity-promoting RRAP formulation

In this appendix, we present the equity-promoting formulations for the RRAP with each measure listed in Table 3.

### E-Companion Section A.1. Equity-promoting RRAP with range

$$\begin{aligned}& \mathop {\mathrm {\text {maximize}}}\limits _{\pmb {v,x,z},u^{\min } \ge 0,u^{\max }}\quad \sum _{r\in R}\sum _{d\in D}\sum _{w\in W^{\text {vac}}_{r}}v_{r,d,w} \\& \mathop {\mathrm {\text {minimize}}}\limits _{\pmb {v,x,z},u^{\min },u^{\max }}\quad u^{\max }-u^{\min } \\& \text {subject to:}\quad (1b)-(1m), \\&\ u^{\max }\ge \sum _{w\in W^{\text {vac}}_{r}}\sum _{d\in D}v_{r,d,w}, \forall r\in R , \\& u^{\min }\le \sum _{w\in W^{\text {vac}}_{r}}\sum _{d\in D}v_{r,d,w}, \ \forall r\in R. \end{aligned}$$

### E-Companion Section A.2. Equity-promoting RRAP with Gini

$$\begin{aligned} & \mathop {\mathrm {\text {maximize}}}\limits _{\pmb {v,x,z,y}}\quad \sum _{r\in R}\sum _{d\in D}\sum _{w\in W^{\text {vac}}_{r}}v_{r,d,w} \\& \mathop {\mathrm {\text {minimize}}}\limits _{\pmb {v,x,z,y}}\quad \sum _{(r,r')\in R\times R\setminus \{r\}}y_{r,r'} \\& \text {subject to:}\quad (1b)-(1m), \\& y_{r,r'}\ge \sum _{w\in W^{\text {vac}}_{r}}\sum _{d\in D}v_{r,d,w}-\sum _{w\in W^{\text {vac}}_{r'}}\sum _{d\in D}v_{r',d,w},\quad \\& \forall (r,r')\in R\times R\setminus \{r\},\\&y_{r,r'}\ge \sum _{w\in W^{\text {vac}}_{r'}}\sum _{d\in D}v_{r',d,w}-\sum _{w\in W^{\text {vac}}_{r}}\sum _{d\in D}v_{r,d,w},\quad \\& \forall (r,r')\in R\times R\setminus \{r\}. \end{aligned}$$

### E-Companion Section A.3. Equity-promoting RRAP with MaxPair

$$\begin{aligned}& \mathop {\mathrm {\text {maximize}}}\limits _{\pmb {v,x,z},y}\quad \sum _{r\in R}\sum _{d\in D}\sum _{w\in W^{\text {vac}}_{r}}v_{r,d,w} \\& \mathop {\mathrm {\text {minimize}}}\limits _{\pmb {v,x,z},y}\quad y\\& \text {subject to:}\quad (1b)-(1m), \\& y\ge \sum _{w\in W^{\text {vac}}_{r}}\sum _{d\in D}v_{r,d,w} -\sum _{w\in W^{\text {vac}}_{r'}}\sum _{d\in D}v_{r',d,w},\quad \\& \forall (r,r')\in R\times R\setminus \{r\},\\&y\ge \sum _{w\in W^{\text {vac}}_{r'}}\sum _{d\in D}v_{r',d,w}-\sum _{w\in W^{\text {vac}}_{r}}\sum _{d\in D}v_{r,d,w},\quad \\& \forall (r,r')\in R\times R\setminus \{r\}. \end{aligned}$$

### E-Companion Section A.4. Equity-promoting RRAP with MeanDev

$$\begin{aligned} & \mathop {\mathrm {\text {maximize}}}\limits _{\pmb {v,x,z,y}}\quad \sum _{r\in R}\sum _{d\in D}\sum _{w\in W^{\text {vac}}_{r}}v_{r,d,w} \\& \mathop {\mathrm {\text {minimize}}}\limits _{\pmb {v,x,z,y}}\quad \sum _{r\in R}y_{r} \\&\text {subject to:}\quad (1b)-(1m), \\& y_{r}\ge \sum _{w\in W^{\text {vac}}_{r}}\sum \limits _{d\in D}v_{r,d,w}-\frac{\sum \limits _{r'\in R}\sum \limits _{w\in W^{\text {vac}}_{r'}}\sum \limits _{d\in D}v_{r',d,w}}{|R|},\quad \\& \forall r\in R,\\&y_{r}\ge \frac{\sum \limits _{r'\in R}\sum \limits _{w\in W^{\text {vac}}_{r'}}\sum \limits _{d\in D}v_{r',d,w}}{|R|}-\sum _{w\in W^{\text {vac}}_{r}}\sum _{d\in D}v_{r,d,w},\quad \\& \forall r\in R. \end{aligned}$$

### E-Companion Section A.5. Equity-promoting RRAP with MaxMeanDev

$$\begin{aligned}& \mathop {\mathrm {\text {maximize}}}\limits _{\pmb {v,x,z},y}\quad \sum _{r\in R}\sum _{d\in D}\sum _{w\in W^{\text {vac}}_{r}}v_{r,d,w} \\& \mathop {\mathrm {\text {minimize}}}\limits _{\pmb {v,x,z},y}\quad y\\& \text {subject to:}\quad (1b)-(1m), \\&\ \ y\ge \sum _{w\in W^{\text {vac}}_{r}}\sum _{d\in D}v_{r,d,w} -\frac{\sum _{r'\in R}\sum _{w\in W^{\text {vac}}_{r'}}\sum _{d\in D}v_{r',d,w}}{|R|},\quad \\& \forall r\in R,\\&y\ge \frac{\sum _{r'\in R}\sum _{w\in W^{\text {vac}}_{r'}}\sum _{d\in D}v_{r',d,w}}{|R|}-\sum _{w\in W^{\text {vac}}_{r}}\sum _{d\in D}v_{r,d,w},\quad \\& \forall r\in R. \end{aligned}$$

### E-Companion Section A.6. Equity-promoting RRAP with MaxSumPair

$$\begin{aligned}& \mathop {\mathrm {\text {maximize}}}\limits _{\pmb {v,x,z,y},\bar{y}}\quad \sum _{r\in R}\sum _{d\in D}\sum _{w\in W^{\text {vac}}_{r}}v_{r,d,w} \\& \mathop {\mathrm {\text {minimize}}}\limits _{\pmb {v,x,z,y},\bar{y}}\quad \bar{y}\\&\text {subject to:}\quad (1b)-(1m), \\& y_{r,r'}\ge \sum _{w\in W^{\text {vac}}_{r}}\sum _{d\in D}v_{r,d,w}-\sum _{w\in W^{\text {vac}}_{r'}}\sum _{d\in D}v_{r',d,w},\quad \\& \forall (r,r')\in R\times R\setminus \{r\},\\&y_{r,r'}\ge \sum _{w\in W^{\text {vac}}_{r'}}\sum _{d\in D}v_{r',d,w}-\sum _{w\in W^{\text {vac}}_{r}}\sum _{d\in D}v_{r,d,w},\quad \\& \forall (r,r')\in R\times R\setminus \{r\},\\&\bar{y}\ge \sum _{r'\in R\setminus \{r\}}y_{r,r'},\quad \forall r\in R. \end{aligned}$$

### E-Companion Section A.7. Equity-promoting RRAP with SumMaxPair

$$\begin{aligned} \mathop {\mathrm {\text {maximize}}}\limits _{\pmb {v,x,z,y}}&\quad \sum _{r\in R}\sum _{d\in D}\sum _{w\in W^{\text {vac}}_{r}}v_{r,d,w} \\ \mathop {\mathrm {\text {minimize}}}\limits _{\pmb {v,x,z,y}}&\quad \sum _{r\in R} y_{r}\\ \text {subject to:}&\quad (1b)-(1m), \\&y_{r}\ge \sum _{w\in W^{\text {vac}}_{r}}\sum _{d\in D}v_{r,d,w}-\sum _{w\in W^{\text {vac}}_{r'}}\sum _{d\in D}v_{r',d,w},\quad & \forall (r,r')\in R\times R\setminus \{r\},\\&y_{r}\ge \sum _{w\in W^{\text {vac}}_{r'}}\sum _{d\in D}v_{r',d,w}-\sum _{w\in W^{\text {vac}}_{r}}\sum _{d\in D}v_{r,d,w},\quad & \forall (r,r')\in R\times R\setminus \{r\}. \end{aligned}$$

Table 7 summarizes the additional number of constraints and variables required to represent each measure in each equity-promoting RRAP model.

**Table 7:** Number of additional variables and constraints required to represent each inequity measure

	Range	Gini	MaxPair	MeanDev	MaxMeanDev	MaxSumPair	SumMaxPair
No. variables	2	$|R|^{2}$	1	|*R*|	1	$|R|^{2}+1$	|*R*|
No. constraints	$2|R|+1$	$2|R|^{2}$	$|R|^{2}$	2|*R*|	2|*R*|	$2|R|^{2}+|R|$	$2|R|^{2}$

## E-Companion Section B. Mathematical proofs

### E-Companion Section B.1. Proof of proposition 1

#### Proof

Note that $(\pmb {z}^0,\pmb {x}^0,\pmb {v}^0)$ satisfies constraints Eqs. (1b)-(1m). Hence, it is a feasible solution to problem Eq. 4, i.e., $(\pmb {z}^0,\pmb {x}^0,\pmb {v}^0) \in \mathcal {F}$. Next, we show that $(\pmb {z}^0,\pmb {x}^0,\pmb {v}^0) \in \mathcal {F}_p$. To show this, we need to verify that $f_{1}(\pmb {v})\le f_{1}(\pmb {v}^0)$ and $f_{2}(\pmb {v}^0)\le f_{2}(\pmb {v})$ for any $(\pmb {z},\pmb {x},\pmb {v})\in \mathcal {F}$. Since $(\pmb {z}^0,\pmb {x}^0,\pmb {v}^0) \in \mathcal {F}$, $f_{2}(\pmb {v}^0)=0$, and $f_{1}(\pmb {v}) \ge 0$ for any $\pmb {v}\in \mathcal {F}$, we have $f_{2}(\pmb {v})\ge f_{2}(\pmb {v}^0)=0$ for all $(\pmb {z},\pmb {x},\pmb {v})\in \mathcal {F}$. Moreover, for any feasible solution $(\pmb {z},\pmb {x},\pmb {v})\in \mathcal {F}$ such that $f_{2}(\pmb {v})=0$, we have

$$\begin{aligned} f_{1}(\pmb {v}^0)=\sum _{r\in R}\sum _{b\in B}\sum _{w\in W^{\text {vac}}_{r}}v^{0}_{r,d,w}\ge \sum _{r\in R}\sum _{b\in B}\sum _{w\in W^{\text {vac}}_{r}}v_{r,d,w}=f_{1}(\pmb {v}), \end{aligned}$$

where the first inequality follows from the optimality of $\pmb {v}^0$. It follows that $(\pmb {z}^0,\pmb {x}^0,\pmb {v}^0) \in \mathcal {F}_{p}$ and $(f_{1}(\pmb {v}^0),f_{2}(\pmb {v}^0))\in \mathcal {P}$. This completes the proof. $\square$

### E-Companion Section B.2. Proof of proposition 2

#### Proof

Note that $(\pmb {z}^l,\pmb {x}^l,\pmb {v}^l)$ satisfies constraints Eqs. (1b)-(1m). Hence it is a feasible solution to problem Eq. 4, i.e., $(\pmb {z}^l,\pmb {x}^l,\pmb {v}^l)\in \mathcal {F}$. Next, we show that $(\pmb {z}^l,\pmb {x}^l,\pmb {v}^l)\in \mathcal {F}_{p}$. To show this, we need to verify that $f_{1}(\pmb {v})\le f_{1}(\pmb {v}^l)$ and $f_{2}(\pmb {v}^l)\le f_{2}(\pmb {v})$, for any $(\pmb {z},\pmb {x},\pmb {v})\in \mathcal {F}$. Since $(\pmb {z}^l,\pmb {x}^l,\pmb {v}^l)\in \mathcal {F}$, $f_{1}(\pmb {v}^l)=\bar{V}$ and $f_{1}(\pmb {v})\le \bar{V}$ for any $\pmb {v}\in \mathcal {F}$, we have $f_{1}(\pmb {v})\le f_{1}(\pmb {v}^l)=\bar{V}$. Moreover, for any feasible solution $(\pmb {z},\pmb {x},\pmb {v})\in \mathcal {F}$ such that $f_{1}(\pmb {v})=\bar{V}$, we have

$$\begin{aligned} f_{2}(\pmb {v}^l)\le f_{2}(\pmb {v}), \end{aligned}$$

where the inequality follows from optimality of $\pmb {v}^l$. Thus, $(\pmb {z}^l,\pmb {x}^l,\pmb {v}^l) \in \mathcal {F}_{p}$ and $(f_{1}(\pmb {v}^l),f_{2}(\pmb {v}^l))\in \mathcal {P}$. $\square$

### E-Companion Section B.3. Proof of proposition 3

#### Proof

We define $\pmb {k}\in \mathbb {R}^{\bar{w}+1}$ as the vacation requests distribution vector associated with vacation schedule $\pmb {v}$, where the *i*th entry of $\pmb {k}=[k_{0},k_{1},k_{2},\dots ,k_{\bar{w}}]$ represents the number of residents with *i* satisfied requests and $\bar{w}$ the is the maximum number of requested weeks for vacation among all residents (i.e., $\bar{w}=\max _{r\in R}|W^{\text {vac}}_{r}|$). Note that for each resident $r\in R$, $u_{r}$ is the number of satisfied vacation requests for resident *r* ($u_{r}=\sum _{d\in D}\sum _{w\in W^{\text {vac}}_{r}}v_{r,d,w}$), and $\bar{u}$ is the average number of satisfied requests. We let $\psi (\pmb {k})$ represent the value of a given inequity measure for a given $\pmb {k}$. We assume that there are at least two residents, i.e., $|R|\ge 2$. Next, we show that fulfilling a request for one of the residents with the fewest satisfied requests while keeping the satisfied vacation requests for residents $r \in \mathcal {V}$ in the new vacation schedule the same as in $\pmb {v}$ yields the lowest value of each measure.

#### Range

Let *l* and *s* represent the largest and smallest non-zero entries of $\pmb {k}=[k_{0},k_{1},k_{2},\dots ,k_{\bar{w}}]$ (i.e., $s\le u_{r}\le l,\forall r\in R$). By the definition of the range measure, we have

$$\begin{aligned} \psi (\pmb {k})=\max _{r\in R}u_{r}-\min _{r\in R}u_{r}=l-s. \end{aligned}$$

Suppose we want to fulfill one additional vacation request and let $\pmb {k}'\in \mathbb {R}^{\bar{w}+1}$ represent the resulting vacation request distribution. Consider the following options for fulfilling the additional request.

- a) Fulfill a request of one of the residents with *i* granted requests, where $i=s$. In this case, if $k_{s}\le 1$, we find that $\psi (\pmb {k}')=l-(s+1)=l-s-1$. Otherwise, if $k_{s}\ge 2$, $\psi (\pmb {k}')=l-s$.
- b) Fulfill a request of one of the residents with *i* granted requests, where $i\in (s,l)$. In this case, we observe that $\pmb {k}'$’s largest and smallest non-zero entries *l* and *s* do not change the inequity value using range does not change (i.e., $\psi (\pmb {k}')=l-s$).
- c) Fulfill a request of one resident with *l* granted requests, where $i=l$. In this case, since we have one resident with $l+1$ satisfied requests, the largest non-zero entry of $\pmb {k}'$ becomes $l+1$. Thus, the inequity value using range is $\psi (\pmb {k}')=l+1-s$.

From (a)–(c), we conclude that fulfilling an additional request of a resident with the fewest satisfied vacation requests will either decrease the value of the range or keep it unchanged.

#### Gini

Given $\pmb {v}$ and its corresponding vacation requests distribution $\pmb {k}\in \mathbb {R}^{\bar{w}+1}$, we compute Gini as

15 $$\begin{aligned}& \psi (\pmb {v})=\sum _{r\in R}\sum _{r'\in R}|u_{r}-u_{r'}|\nonumber \\&=k_{0}\big (k_{1}+\dots +\bar{w}k_{\bar{w}}\big )+ \\& k_{2}\big (2k_{0}+k_{1}+\dots +(\bar{w}-1)k_{\bar{w}}\big )\dots \\& +k_{\bar{w}}\big (\bar{w}k_{0}+(\bar{w}-1)k_{1}+\dots + k_{\bar{w}-1}\big ) \nonumber \\&= \sum _{i=0}^{\bar{w}}k_{i}\big ( \sum _{j=0}^{\bar{w}}|j-i|k_{j} \big ), \end{aligned}$$

Let $p\in [1,\bar{w}-1]$ and consider the following two options for fulfilling one additional request.

- (a) Fulfill a request of one of the residents with $p-1$ granted requests. The new vacation request distribution is $\pmb {k}'= [k_{0},k_{p-1}-1,k_{p}+1,\dots ,k_{\bar{w}}]$ and the Gini deviation $\psi (k')$ is as follows.
  $$\begin{aligned}& \psi (\pmb {k}')=\underset{ i\ne p-1,p}{\underset{i\in [0,\bar{w}]:}{\sum }}k_{i}\\&\bigg [ \underset{j\ne [p-1,p]}{\underset{j\in [0,\bar{w}]:}{\sum }} |j-i|k_{j}+|p-1-i| (k_{p-1}-1) \\& +|p-i|(k_{p}+1) \bigg ]+(k_{p-1}-1)\\&\bigg [ \underset{j\ne p}{\underset{j\in [0,\bar{w}]:}{\sum }}|j-(p-1)|k_{j}+k_{p}+1 \bigg ]+(k_{p}+1) \\& \bigg [ \underset{j\ne p-1}{\underset{j\in [0,\bar{w}]:}{\sum }} |j-p|k_{j}+k_{p-1}-1 \bigg ] \end{aligned}$$
  The difference between $\psi (\pmb {k})$ and $\psi (\pmb {k}')$ equals to
  16 $$\begin{aligned}& \psi (\pmb {k}')-\psi (\pmb {k})=\underset{ i\ne p-1,p}{\underset{i\in [0,\bar{w}]:}{\sum }} k_{i}\bigg [ \underset{j\ne [p-1,p]}{\underset{j\in [0,\bar{w}]:}{\sum }} |j-i|k_{j} +|p-1-i|(k_{p-1}-1)\\& +|p-i|(k_{p}+1) \nonumber -\underset{j\ne [p-1,p]}{\underset{j\in [0,\bar{w}]:}{\sum }}|j-i|k_{j}-|p-1-i|k_{p-1}\\& -|p-i|k_{p}\bigg ]\nonumber +k_{p-1}\bigg [ \underset{j\ne p}{\underset{j\in [0,\bar{w}]:} {\sum }}|j-(p-1)|k_{j}+k_{p} \bigg ] -(k_{p-1}-1) \\& \bigg [\underset{j\ne p}{\underset{j\in [0,\bar{w}]:}{\sum }}|j-(p-1)|k_{j}+k_{p}+1 \bigg ] \nonumber \\&+k_{p}\bigg [ \underset{j\ne p-1}{\underset{j\in [0,\bar{w}]:}{\sum }} |j-p|k_{j}+k_{p-1} \bigg ] \\& -(k_{p}+1)\bigg [ \underset{j\ne p-1}{\underset{j\in [0,\bar{w}]:}{\sum }} |j-p|k_{j}+k_{p-1}-1 \bigg ] \nonumber \\&=\underset{i\ne p-1,p}{\underset{i\in [0,\bar{w}]:}{\sum }} k_{i}\bigg [ |p-i|-|p-1-i|\bigg ]-k_{p-1} \\& +\bigg [ \underset{j\ne p}{\underset{j\in [0,\bar{w}]:}{\sum }} |j-(p-1)|k_{j}+k_{p}+1 \bigg ] \\& +k_{p}\nonumber -\bigg [ \underset{j\ne p-1}{\underset{j\in [0,\bar{w}]:}{\sum }} |j-p|k_{j} +k_{p-1}-1 \bigg ] \nonumber \\&= \underset{i\ne p-1,p}{\underset{i\in [0,\bar{w}]:}{\sum }} k_{i}+ \underset{j\ne p-1,p}{\underset{j\in [0,\bar{w}]:}{\sum }} \big (|j-(p-1)|-|j-p|\big )k_{j}+2k_{p}-2k_{p-1}+2 \nonumber \\&=\underset{i\ne p-1,p}{\underset{i\in [0,\bar{w}]:}{\sum }} k_{i}- \underset{j\ne p-1,p}{\underset{j\in [0,\bar{w}]:}{\sum }} k_{j}+2(k_{p}-k_{p-1}+1) =2(k_{p}-k_{p-1}+1) . \end{aligned}$$
- (b) Fulfill a request of one of the residents with *p* granted requests. Let $\pmb {k}''=[k_{0},\dots ,k_{p}-1,k_{p+1}+1,\dots , k_{\bar{w}}]$ represent the resulting vacation request distribution vector. It is easy to verify following the same steps as in (a) that $\psi (\pmb {k}'')-\psi (\pmb {k})=2(k_{p+1}-k_{p}+1)$.

Computing the difference between $\psi (\pmb {k}')-\psi (\pmb {k})$ and $\psi (\pmb {k}'')-\psi (\pmb {k})$

17 $$\begin{aligned} & \big [\psi (\pmb {k}'')-\psi (\pmb {k})\big ]-\big [\psi (\pmb {k}')-\psi (\pmb {k})\big ] \\&=2\big ( k_{p+1}-k_{p}+1-k_{p}+k_{p-1}-1\big )\\& \nonumber=2\big (k_{p+1}+k_{p-1}-2k_{p}\big )\\& \nonumber\ge 2\big [k_{p+1}+k_{p-1}-2(k_{p+1}+k_{p-1}-|R|)\big ]\\& \nonumber\ge 2\big [ 2|R|-(k_{p+1}+k_{p-1}) \big ]>0. \end{aligned}$$

The last inequality holds because $k_{p+1}+k_{p}+k_{p-1}\le \sum \limits _{i=0}^{n}k_{i}=|R|$. Consequently, we conclude that fulfilling a vacation request for one of the residents with $p\in [1,\bar{w}-1]$ satisfied requests results in a lower increment in the value of the inequity measure (Gini deviation) than fulfilling a request of one of the residents with $p+1$ request. Since we show this for arbitrary *p*, this establishes that fulfilling a vacation request for one of the residents with the fewest satisfied requests always yields the lowest value of the Gini deviation.

#### Maximum pairwise deviation

We first show that the value of the maximum pairwise deviation equals that of the range for any vacation request distribution $\pmb {k}\in \mathbb {R}^{\bar{w}+1}$. Recall that *s* and *l* respectively represent the smallest and largest entries of $\pmb {k}$ such that $k_{i}> 0,\forall i\in [0,\bar{w}]$, i.e., $s\le u_{r}\le l,\forall r\in R$. It follows that we can compute the maximum pairwise deviation as

$$\begin{aligned} \max _{r\in R}\max _{r'\in R}|u_{r}-u_{r'}|=\max _{r\in R}|u_{r}-s|=l-s= \max _{r\in R}u_{r}-\min _{r\in R}u_{r}. \end{aligned}$$

This shows that the range and maximum pairwise deviation are equivalent. It follows from point (1) that the results hold for maximum pairwise deviation.

#### Absolute deviation from mean

By the definition of the absolute deviation from the mean, we have

18 $$\begin{aligned} \psi (\pmb {k})=\sum _{r\in R}|u_{r}-\bar{u}|&=k_{0}\bigg |\frac{\sum _{j=0}^{\bar{w}}jk_{j}}{\sum _{j=0}^{\bar{w}}k_{j}}\bigg |+\dots +k_{\bar{w}}\bigg |\bar{w}-\frac{\sum _{j=0}^{\bar{w}}jk_{j}}{\sum _{j=0}^{\bar{w}}k_{j}}\bigg | \nonumber \\&=\sum _{i=0}^{\bar{w}}k_{i}\bigg |i-\frac{\sum _{j=0}^{\bar{w}}jk_{j}}{|R|} \bigg |=\sum _{i=0}^{\bar{w}}k_{i}\bigg |i-\frac{S}{|R|} \bigg |, \end{aligned}$$

where $S=\sum _{j=0}^{\bar{w}}jk_{j}$ represents the total number of satisfied vacation requests and $|R|=\sum _{j=0}^{\bar{w}}k_{j}$.

Now suppose we want to fulfill one additional vacation request. Suppose we fulfill a request for one of the residents with currently $p-1$ granted requests, where $p\in [1,\bar{w}-1]$. The new vacation request distribution is $\pmb {k}'= [k_{0}, \dots , k_{p-1}-1,k_{p}+1,\dots ,k_{\bar{w}}]$. By Eq. 18, we have

$$\begin{aligned} & \psi (\pmb {k}')=\underset{i\ne p-1,p}{\underset{i\in [0,\bar{w}]:}{\sum }} k_{i}\bigg |i-\frac{S+1}{|R|}\bigg |+(k_{p-1}-1) \\& \bigg | p-1-\frac{S+1}{|R|}\bigg | +(k_{p}+1)\bigg | p-\frac{S+1}{|R|}\bigg | \end{aligned}$$

Computing the difference between $\psi (\pmb {k}')$ and $\psi (\pmb {k})$, we obtain

19 $$\begin{aligned}& \psi (\pmb {k}')-\psi (\pmb {k})= \underset{i\ne p-1,p}{\underset{i\in [0,\bar{w}]:}{\sum }} k_{i}\bigg |i-\frac{S+1}{|R|}\bigg |+(k_{p-1}-1) \\& \bigg |p-1-\frac{S+1}{|R|}\bigg | +(k_{p}+1) \bigg |p-\frac{S+1}{|R|}\bigg |\nonumber \\&-\bigg [ \underset{i\ne p-1,p}{\underset{i\in [0,\bar{w}]:}{\sum }} k_{i}\big |i-\frac{S}{|R|}\big |+(k_{p-1})\big |p-1-\frac{S}{|R|}\big | \\& +(k_{p})\big |p-\frac{S}{|R|}\big |\bigg ]\nonumber =\sum _{i=0}^{\bar{w}}k_{i}\bigg [\big | i-\frac{S+1}{|R|}\big |- \\& \big | i-\frac{S}{|R|}\big |\bigg ] + \bigg |p-\frac{S+1}{|R|}\bigg |-\bigg |p-1-\frac{S+1}{|R|}\bigg | . \end{aligned}$$

Now suppose that we instead fulfill an additional request of a resident with *p* granted request and obtain the vacation request distribution vector $\pmb {k}''=[k_{0},\dots ,k_{p}-1,k_{p+1}+1,\dots , k_{\bar{w}}]$. Using Eq. 18 and following the same techniques as in Eq. 19, we compute $\psi (\pmb {k}'')-\psi (\pmb {k})$ as

20 $$\begin{aligned}& \psi (\pmb {k}'')-\psi (\pmb {k})=\sum _{i=0}^{\bar{w}}k_{i}\bigg [\bigg | i-\frac{S+1}{|R|}\bigg |-\bigg | i-\frac{S}{|R|}\bigg |\bigg ] \\& + \bigg |p+1-\frac{S+1}{|R|}\bigg | -\bigg |p-\frac{S+1}{|R|}\bigg |. \end{aligned}$$

Subtracting Eq. 20 from Eq. 19, we have

21 $$\begin{aligned} & \big [\psi (\pmb {k}'')-\psi (\pmb {k})\big ]-\big [\psi (\pmb {k}')-\psi (\pmb {k})\big ]\\&= \bigg |p+1-\frac{S+1}{|R|}\bigg | -\bigg |p-\frac{S+1}{|R|}\bigg | \\& -\bigg |p-\frac{S+1}{|R|}\bigg | +\bigg |p-1-\frac{S+1}{|R|}\bigg | \nonumber \\&=\bigg |p+1-\frac{S+1}{|R|}\bigg |-2\bigg |p-\frac{S+1}{|R|}\bigg | \\& +\bigg |p-1-\frac{S+1}{|R|}\bigg | . \end{aligned}$$

Equation 21 shows, using the absolute deviation from the mean as the inequity measure, the gap in the inequity value between fulfilling the extra request from a resident with *p* granted requests and $p-1$ granted requests. Consider the following three cases:

- a) $p\le \frac{S+1}{|R|}-1$ or $p\ge \frac{S+1}{|R|}+1$. We first consider the case when $p\ge \frac{S+1}{|R|}+1$. The same proof techniques can be used for the case when $p\le \frac{S+1}{|R|}-1$. When $p+1>p\ge \frac{S+1}{|R|}+1$, we have
  $$\begin{aligned}& \big [\psi (\pmb {k}'')-\psi (\pmb {k})\big ]-\big [\psi (\pmb {k}')-\psi (\pmb {k})\big ]= \bigg (p+1-\frac{S+1}{|R|}\bigg ) \\&-2\bigg (p-\frac{S+1}{|R|}\bigg )+\bigg (p-1-\frac{S+1}{|R|}\bigg )=0. \end{aligned}$$
- b) $\frac{S+1}{|R|} \le p\le \frac{S+1}{|R|}+1$. In this case, $p+1\ge p\ge \frac{S+1}{|R|}$ but $p-1 \le \frac{S+1}{|R|}$. Then we have,
  $$\begin{aligned}& \big [\psi (\pmb {k}'')-\psi (\pmb {k})\big ]-\big [\psi (\pmb {k}')-\psi (\pmb {k})\big ]\\&=\bigg (p+1-\frac{S+1}{|R|}\bigg )-2\bigg (p-\frac{S+1}{|R|}\bigg ) \\& +\bigg (\frac{S+1}{|R|}-p+1\bigg )\\&=2\bigg [\frac{S+1}{|R|}-p+1\bigg ]\ge 0. \end{aligned}$$
- c) $\frac{S+1}{|R|}-1\le p\le \frac{S+1}{|R|}$. In this case, $p+1\ge \frac{S+1}{|R|}$ but $p-1\le p \le \frac{S+1}{|R|}$, then we have
  $$\begin{aligned} &\big [\psi (\pmb {k}'')-\psi (\pmb {k})\big ]-\big [\psi (\pmb {k}')-\psi (\pmb {k})\big ]\\&=\bigg (p+1-\frac{S+1}{|R|}\bigg )-2\bigg (\frac{S+1}{|R|}-p\bigg )\\&+\bigg (\frac{S+1}{|R|}-p+1\bigg )\\&=2\bigg [p-\frac{S+1}{|R|}+1\bigg ]\ge 0. \end{aligned}$$

From (a)–(c), we conclude that $\big [\psi (\pmb {k}'')-\psi (\pmb {k})\big ]\ge \big [\psi (\pmb {k}')-\psi (\pmb {k})\big ]$. It follows that fulfilling a request of a resident with *p* requests could result in a larger increment in the value of the inequity measure (absolute deviation from the mean) compared with fulfilling a request to a resident with $p-1$ requests. Thus, it is optimal to fulfill a request of a resident with the fewest satisfied requests.

#### Maximum absolute deviation from mean

Recall that *s* and *l* respectively represent the smallest and largest entries of $\pmb {k}$ such that $k_{i}> 0,\forall i\in [0,\bar{w}]$, i.e., $s\le u_{r}\le l,\forall r\in R$. By definition of maximum absolute deviation from the mean, we have

22 $$\begin{aligned} \psi (\pmb {k})=\max _{r\in R}|u_{r}-\bar{u}|&=\max \big \{|s-\bar{u}|,|s+1-\bar{u}|,\dots ,|l-\bar{u}|\big \}\nonumber \\&=\max \bigg \{\big |s-\frac{S}{|R|}\big |,\dots , \big |l-\frac{S}{|R|}\big |\bigg \}\nonumber \\&=\max \bigg \{ \big |s-\frac{S}{|R|}\big |,\big |l-\frac{S}{|R|}\big | \bigg \}, \end{aligned}$$

where *S* is the total number of satisfied requests, i.e., $S=\sum _{r\in R}u_{r}$. Since the total number of satisfied vacations $S\in [|R|s,|R|l]$, the average number of satisfied requests $\frac{S}{|R|}\in [s,l]$. Thus, Eq. a request of one of the residents with22 reduces to

23 $$\begin{aligned} \psi (\pmb {k})=\max \bigg \{ \big (\frac{S}{|R|}-s\big ),(l-\frac{S}{|R|}\big )\bigg \}. \end{aligned}$$

Suppose we want to fulfill one additional vacation request to a resident and let $\pmb {k}'$ represent the resulting new vacation request distribution. Then $\psi (\pmb {k}')$ equalsthe average number of satisfied requests

24 $$\begin{aligned} \psi (\pmb {k}')=\max \bigg \{ \big (\frac{S+1}{|R|}-s'\big ), \big (l'-\frac{S+1}{|R|}\big ) \bigg \}, \end{aligned}$$

where $s'$ and $l'$ represent the smallest and largest non-zero entries of $\pmb {k}'$, respectively. Now, consider the following cases.

- a) Fulfill a request of one of the residents with *s* granted requests. First, when $k_{s}=1$ (there is one resident with *s* granted requests), we have $s'=s+1$ and $l'=l$. By Eq. 24,
  25 $$\begin{aligned} \psi (\pmb {k}')=\max \bigg \{ \Big (\frac{S+1}{|R|}-(s+1)\Big ), \Big (l-\frac{S+1}{|R|}\Big ) \bigg \}, \end{aligned}$$
  Consider the following three sub-cases:
  - i)$\frac{l+s}{2}\le \frac{S+1}{|R|}-\frac{1}{2}$. In this case, we have $l-\frac{S+1}{|R|}\le$ $\frac{S+1}{|R|}-(s+1)$. Thus, $\psi (\pmb {k}')=\frac{S+1}{|R|}-(s+1)$. Note also that $\frac{l+s}{2}\le \frac{S}{|R|}+ \frac{1}{|R|}-\frac{1}{2} \le \frac{S}{|R|}$ (since $|R|\ge 2$). Hence, $l-\frac{S}{|R|}\le \frac{S}{|R|}-s$ and $\psi (\pmb {k})=\big (\frac{S}{|R|}-s\big )$. The difference between $\psi (\pmb {k}')$ and $\psi (\pmb {k})$ equals
    $$\begin{aligned} \psi (\pmb {k}')-\psi (\pmb {k})=\frac{S+1}{|R|}-s-1-\Big (\frac{S}{|R|}-s\Big )=\frac{1}{|R|}-1 < 0. \end{aligned}$$
  - ii)$\frac{l+s}{2}\in [\frac{S+1}{|R|}-\frac{1}{2},\frac{S}{|R|}]$. Since $\frac{l+s}{2}\ge \frac{S+1}{|R|}-\frac{1}{2}$, we have $l-\frac{S+1}{|R|}\ge \frac{S+1}{|R|}-(s+1)$. Moreover, since $\frac{l+s}{2}\le \frac{S}{|R|}$, we have $l-\frac{S}{|R|}\le \frac{S}{|R|}-s$. Thus, $\psi (\pmb {k})=\frac{S}{|R|}-s$ and $\psi (\pmb {k}')=l-\frac{S+1}{|R|}$, and
    $$\begin{aligned} \psi (\pmb {k}')-\psi (\pmb {k})=l-\frac{S+1}{|R|}-\Big (\frac{S}{|R|}-s\Big )=l+s-\frac{2S+1}{|R|} \le \frac{2S}{|R|}-\frac{2S+1}{|R|}<0. \end{aligned}$$
  - iii)$$\frac{l+s}{2}\ge \frac{S}{|R|}$$. In this case, we have $$l-\frac{S}{|R|} \ge s- \frac{S}{|R|}$$ and $$\psi (\pmb {k})=\big (l-\frac{S}{|R|}\big )$$. Note that $$l+s \ge \frac{2S}{|R|} \ge \frac{2S}{|R|}+(\frac{1}{|R|}-1)$$ (since $$|R|\ge 2$$). Thus, $$l-\frac{S+1}{|R|}\ge$$$$\frac{S+1}{|R|}-(s+1)$$ and so $$\psi (\pmb {k}')=$$$$\big (l-\frac{S+1}{|R|}\big )$$. The difference between $$\psi (\pmb {k}')$$ and $$\psi (\pmb {k})$$ equals
    $$\begin{aligned} \psi (\pmb {k}')-\psi (\pmb {k})=l-\frac{S+1}{|R|}-l+\frac{S}{|R|}=-\frac{1}{|R|}<0. \end{aligned}$$
  We conclude that when $k_{s}=1$, fulfilling a request of a resident with *s* granted request (i.e., the one with the fewest number of satisfied requests) will decrease the value of $\psi$. Now, consider the case when $k_{s}\ge 2$ (there are more than two residents with *s* granted requests). By Eq. 24, we have
  26 $$\begin{aligned} \psi (\pmb {k}')=\max \bigg \{ \big (\frac{S+1}{|R|}-s\big ), \big (l-\frac{S+1}{|R|}\big ) \bigg \}. \end{aligned}$$
  Then we consider the following three sub-cases.
  - i) $\frac{l+s}{2}\le \frac{S}{|R|}$. In this case, $l+s\le \frac{2S}{|R|}$ and $\frac{S}{|R|}-s\ge l-\frac{S}{|R|}$. Moreover, since $\frac{l+s}{2}\le \frac{S+1}{|R|}$, we have $\frac{S+1}{|R|}-s\ge \frac{S+1}{R}-l$. It follows that $\psi (\pmb {k})=\frac{S}{|R|}-s$, $\psi (\pmb {k}')=\frac{S+1}{|R|}-s$, and
    $$\begin{aligned} \psi (\pmb {k}')-\psi (\pmb {k})=\frac{S+1}{|R|}-s-\frac{S}{|R|}+s=\frac{1}{|R|}. \end{aligned}$$
  - ii) $\frac{S}{|R|} \le \frac{l+s}{2}\le \frac{S+1}{|R|}$. In this case, we have $l-\frac{S}{|R|}\ge \frac{S}{|R|}-s$. Moreover, since $\frac{l+s}{2}\le \frac{S+1}{|R|}$, by point (i) when $k_{s}\ge 2$, $\frac{S+1}{|R|}-s\ge \frac{S+1}{R}-l$. Hence, $\psi (\pmb {k})=l-\frac{S}{|R|}$, $\psi (\pmb {k}')=\frac{S+1}{|R|}-s$, and
    $$\begin{aligned} \psi (\pmb {k}')-\psi (\pmb {k})=\frac{S+1}{|R|}-s-l+\frac{S}{|R|}=\frac{2S+1}{|R|}-(l+s). \end{aligned}$$
    Since $$\frac{l+s}{2}\in [S/|R|,(S+1)/|R|]$$, $$\psi (\pmb {k}')-\psi (\pmb {k})\in [-1/|R|,1/|R|]$$.
  - iii) $\frac{l+s}{2}\ge \frac{S+1}{|R|}$. In this case, we have $l-\frac{S}{|R|}\ge \frac{S}{|R|}-s$ and $l-\frac{S+1}{|R|}\ge \frac{S+1}{|R|}-s$. Hence, $\psi (\pmb {k})=l-\frac{S}{|R|}$, $\psi (\pmb {k}')=l-\frac{S+1}{|R|}$, and
    $$\begin{aligned} \psi (\pmb {k}')-\psi (\pmb {k})=l-\frac{S+1}{|R|}-l+\frac{S}{|R|}=-\frac{1}{|R|}. \end{aligned}$$
  Consequently, we conclude that when $k_{s}\ge 2$, (i) fulfilling a request to a resident with the fewest satisfied requests will lead to either an increase or decrease in the inequity value; (ii) If the inequity value increases, the increase in the inequity value is at most $\frac{1}{|R|}$; (iii) If the inequity value decreases, the decrease in the inequity value is at most $\frac{1}{|R|}$.
- b) Fulfill a request to one of the residents with *i* requests, where $i\in [s+1,l-1]$. In this case, $\psi (\pmb {k})$ equals Eq. 25, and the proof is the same as point (a) when $k_{s}\ge 2$. Hence, fulfilling an additional request to a resident with $[s+1,l-1]$ satisfied requests will lead to either an increase or decrease in the inequity value. The change in the inequity value is at most $\frac{1}{|R|}$.
- c) Fulfill a request of one of the residents with *l* requests. Let us consider the first case when $k_{l}\ge 2$. In this case, $\psi (\pmb {k})$ equals Eq. 25. The proof is the same as point (a) when $k_{s}\ge 2$. Now we consider the second case when there is only one resident with *l* granted requests ($k_{l}=1$). By Eq. 25, we have
  $$\begin{aligned} \psi (\pmb {k}')=\max \bigg \{\Big (\frac{S+1}{|R|}-s\Big ),\Big (l+1-\frac{S+1}{|R|}\Big )\bigg \}. \end{aligned}$$
  Now consider the following three sub-cases.
  - i) $\frac{l+s}{2}\le \frac{S+1}{|R|}-\frac{1}{2}$. In this case, since $l+s\le \frac{2S+2}{|R|}-1$, we have $(l+1)-\frac{S+1}{|R|}\le \frac{S+1}{|R|}-s$. As in point (a)–(i), we have $l-\frac{S}{|R|}\le \frac{S}{|R|}-s$. Hence, $\psi (\pmb {k})=\frac{S}{|R|}-s$ and $\psi (\pmb {k}')=\frac{S+1}{|R|}-s$, Thus,
    $$\begin{aligned} \psi (\pmb {k}')-\psi (\pmb {k})=\frac{S+1}{|R|}-s-\frac{S}{|R|}+s=\frac{1}{|R|}>0. \end{aligned}$$
  - ii) $$\frac{S+1}{|R|}-\frac{1}{2}\le \frac{l+s}{2}\le \frac{S}{|R|}$$. In this case, as in point (a)–ii, we have $$l-\frac{S}{|R|}\le \frac{S}{|R|}-s$$ and $$\psi (\pmb {k})=\frac{S}{|R|}-s$$. Moreover, since $$l+s\ge \frac{2S+2}{|R|}-1$$, we have $$(l+1)-\frac{S+1}{|R|}$$$$\ge \frac{S+1}{|R|}-s$$ and $$\psi (\pmb {k}')=l$$$$+1-\frac{S+1}{|R|}$$. Hence,
    $$\begin{aligned}& \psi (\pmb {k}')-\psi (\pmb {k})=l+1-\frac{S+1}{|R|}-\frac{S}{|R|}+s\\&=(l+s+1)-\frac{2S+1}{|R|}\ge \frac{2S+2}{|R|}-\frac{2S+1}{|R|}>0. \end{aligned}$$
  - iii) $\frac{l+s}{2}\ge \frac{S}{|R|}$. In this case, as in point (a)–iii, we have $\psi (\pmb {k})=(l-\frac{S}{|R|})$. Moreover, $l+1-\frac{S+1}{|R|}\ge \frac{S+1}{|R|}-s$ and $\psi (\pmb {k}')=l+1-\frac{S+1}{|R|}$. Thus,
    $$\begin{aligned} \psi (\pmb {k}')-\psi (\pmb {k})=l+1-\frac{S+1}{|R|}-l+\frac{S}{|R|}=1-\frac{1}{|R|}\ge 0. \end{aligned}$$
  Consequently, we conclude that when $k_{l}=1$, fulfilling an additional request to a resident with the maximal number of satisfied requests will (i) lead to an increase in the inequity value; (ii) an increase in the inequality value is at least $\frac{1}{|R|}$. Furthermore, when $k_{l}\ge 2$, fulfilling an additional request to a resident with the maximal number of satisfied requests will lead to either an increase or decrease in the inequity value. The change in the inequity value is at most $\frac{1}{|R|}$.

The above analyses show that fulfilling additional request of a resident with the fewest granted requests is optimal because (i) it leads to the maximal decrease in the inequity value, i.e., the decrease in the inequity value is at least 1/|*R*| when $k_{s}=1$; (ii) it leads to a minimal change in the inequity value, i.e., the change in the inequity value is at most 1/|*R*| when $k_{s}\ge 2$.

#### Maximum sum of pairwise deviation

We first claim that, for any vacation request distribution, the maximum sum of pairwise deviation equals a constant multiplied by the maximum absolute deviation from the mean, i.e., $\pmb {k}\in \mathbb {R}^{\bar{w}+1}$, $\max _{r\in R}\sum _{r'\in R}|u_{r}-u_{r'}| = |R|\max _{r\in R}|u_{r}-\bar{u}|$. To prove this claim, we rewrite the maximum sum of pairwise deviation as

27 $$\begin{aligned}& \psi (\pmb {k})=\max _{r\in R}\sum _{r'\in R}|u_{r}-u_{r'}| \nonumber \\&=\max _{i\in [0,\bar{w}]}\bigg \{\sum _{j=0}^{\bar{w}}k_{j}|j-i|\bigg \}\\&=\max \bigg \{ \sum _{i=0}^{\bar{w}}ik_{i},\sum _{i=0}^{\bar{w}}(\bar{w}-i)k_{i} \bigg \}\\&=\max \{c_{1},c_{2}\}, \end{aligned}$$

where $c_{1}$ and $c_{2}$ represent the first and second expressions in the max operator, respectively. We first consider the case when $c_{1}\le c_{2}$. In this case, we have $\psi (\pmb {k})=\sum _{i=0}^{\bar{w}}(\bar{w}-i)k_{i}$ and

28 $$\begin{aligned} \sum _{i=0}^{\bar{w}}ik_{i}&\le \bar{w}\sum _{i=0}^{\bar{w}}k_{i}-\sum _{i=0}^{\bar{w}}ik_{i} \nonumber \\ \frac{\sum _{i=0}^{\bar{w}}ik_{i}}{\sum _{i=0}^{\bar{w}}k_{i}}&\le \frac{\bar{w}\sum _{i=0}^{\bar{w}}k_{i}-\sum _{i=0}^{\bar{w}}ik_{i}}{\sum _{i=0}^{\bar{w}}k_{i}} \nonumber \\ \bar{u}&\le \bar{w}-\bar{u}, \end{aligned}$$

where the last inequality follows from $\bar{u}=\sum _{i=0}^{\bar{w}}ik_{i}/\sum _{i=0}^{\bar{w}}k_{i}$. It follows that when $c_{1}\le c_{2}$, we have

29 $$\begin{aligned} &\psi (\pmb {k})=\sum _{i=0}^{\bar{w}}(\bar{w}-i)k_{i}\\&=\sum _{i=0}^{\bar{w}}\bar{w}k_{i}-\sum _{i=0}^{\bar{w}}ik_{i}=\sum _{i=0}^{\bar{w}}k_{i}(\bar{w}-\bar{u})\nonumber \\&=\sum _{i=0}^{\bar{w}}k_{i}\max \big \{\bar{u},|1-\bar{u}|.\dots ,|\bar{w}-\bar{u}|\big \} \\& (\text {since } \bar{w} - \bar{u} \ge \bar{u} \text { by } (28))\nonumber \\&=\sum _{i=0}^{\bar{w}}k_{i}\max _{i\in [0,\bar{w}]}|i-\bar{u}|=|R|\max _{r\in R}|u_{r}-\bar{u}|. \end{aligned}$$

From 28, we conclude that $$\max _{r\in R}\sum _{r'\in R}|u_{r}-u_{r'}|=|R|\max _{r\in R}|u_{r}-\bar{u}|$$. A similar argument holds when $$c_{1}\ge c_{2}$$. This shows that $$\max _{r\in R}\sum _{r'\in R}$$$$|u_{r}-u_{r'}| =$$$$|R|\max _{r\in R}|u_{r}-\bar{u}|$$. Note that the number of residents |*R*| is constant. Moreover, in point (5), we have shown that the proposition holds for the maximum absolute deviation from the mean $$\max _{r\in R}|u_{r}-\bar{u}|$$. It follows that the results also hold for $$\max _{r\in R}\sum _{r'\in R}|u_{r}-u_{r'}|$$.

#### Sum of maximum pairwise deviation

Recall that *s* and *l* represent the smallest and largest entries of $\pmb {k}$ such that $k_{i}>0,\forall i\in [0,\bar{w}]$ respectively, i.e., $s\le u_{r}\le l,\forall r\in R$. By definition of the sum of maximum pairwise deviation, we have

30 $$\begin{aligned}& \psi (\pmb {k})=\sum _{r\in R}\max _{r'\in R}|u_{r}-u_{r'}| \nonumber \\&=k_{s}(l-s)+k_{s+1}\max \{|(s+1)-s|, \\& |s+2-(s+1)|,\dots ,|l-(s+1)|\}+\dots +k_{l}(l-s)\nonumber \\&=k_{s}(l-s)+k_{s+1}\max \{(s),(l-s-1)\}+\dots +k_{l-1} \\& \max \{(l-1),1\}+k_{l}(l-s) \nonumber \\&=\sum _{i=s}^{l}\max \{(i-s),(l-i)\}k_{i}\nonumber =\\&{\left\{ \begin{array}{ll} \sum _{i=s}^{c_{1}}k_{i}(l-i)+\sum _{i=c_{2}}^{l}k_{i}(i-s) \quad & \text {If (l+s)/2 is fractional}\\ \sum _{i=s}^{c}k_{i}(l-i)+\sum _{i=c+1}^{l}k_{i}(i-s) \quad & \text {Otherwise} \end{array}\right. } \end{aligned}$$

where $$c=\frac{l+s}{2}$$, $$c_{1}=\lfloor \frac{l+s}{2}\rfloor$$ and $$c_{2}=\lceil \frac{l+s}{2}\rceil$$.

In the following, we consider the case when $(l+s)/2$ is fractional. A similar statement holds when $(l+s)/2$ is integer. Now suppose we want to fulfill one additional vacation request to a resident with $p-1$ granted requests and let $\pmb {k}'=[k_{0},\dots ,k_{p-1}-1,k_{p}+1,\dots ,k_{\bar{w}}]$ represent the new vacation request distribution. Consider the following three cases:

- a)$p\le c_{1}-1$. In this case, we have $p-1\le p\le p+1\le c_{1}=\lfloor \frac{l+s}{2}\rfloor$. Then we know
  $$\begin{aligned} &\psi (\pmb {k}')-\psi (\pmb {k})= \underset{i\ne p-1,p}{\underset{i\in [s,c_{1}]:}{\sum }} (l-i)k_{i}+(l-p+1) \\& (k_{p-1}-1)+(l-p)(k_{p}+1)+\sum _{i=c_{2}}^{l}(i-s)k_{i}\\&- \bigg [ \underset{i\ne p-1,p}{\underset{i\in [s,c_{1}]:}{\sum }} (l-i)k_{i}+(l-p+1)(k_{p-1}) \\& +(l-p)(k_{p})+\sum _{i=c_{2}}^{l}(i-s)k_{i} \bigg ]=(l-p+1)(-1)\\&+(l-p)=l-p-l+p-1=-1. \end{aligned}$$
  Now suppose we fulfill an extra request of a resident with *p* fulfilled requests and let $\pmb {k}''$ be the new vacation request distribution. Since $p\le p+1\le c_{1}$, it is easy to verify that $\psi (\pmb {k}'')-\psi (\pmb {k})=-1$. Finally, we conclude that $\psi (\pmb {k}'')-\psi (\pmb {k})-[\psi (\pmb {k}')-\psi (\pmb {k})]=0$.
- b) $p \ge c_{2}+1$. In this case, we have $p+1\ge p\ge p-1\ge c_{2}=\lceil \frac{l+s}{2}\rceil$. Then we know
  $$\begin{aligned}& \psi (\pmb {k}')-\psi (\pmb {k})=\sum _{i=s}^{c_{1}}(l-i)k_{i}+ \underset{p-1,p}{\underset{i\in [c_{2},l]:}{\sum }} (i-s)k_{i} \\&+ (p-1-s)(k_{p-1}-1)+(p-s)(k_{p}+1)\\&-\bigg [\sum _{i=s}^{c_{1}}(l-i)k_{i}+ \underset{p-1,p}{\underset{i\in [c_{2},l]:}{\sum }} (i-s)k_{i}\\&+(p-1-s) (k_{p-1})+(p-s)(k_{p}) \bigg ]\\&=(p-1-s)(-1)+(p-s)\\&=p-s-p+1+s=1 \end{aligned}$$
  Since $p\ge p-1\ge c_{2}$, it is easy to verify that $\psi (\pmb {k}'')-\psi (\pmb {k})=1$. Thus, we conclude that $\psi (\pmb {k}'')-\psi (\pmb {k})-[\psi (\pmb {k}')-\psi (\pmb {k})]=0$.
- c) $c_{1}\le p \le c_{2}$. In this case, since there is no integer between $c_{1}$ and $c_{2}$, we have two sub-cases $p=c_{1}$ or $p=c_{2}$. Let us consider the first sub-case when $p=c_{1}$. In this case, we have $p-1\le p\le c_{1}$. Then by conclusion in a), we have $\psi (\pmb {k}')-\psi (\pmb {k})=-1$. Meanwhile, since $p+1\ge c_{2}\ge p$, we have
  $$\begin{aligned} &\psi (\pmb {k}'')-\psi (\pmb {k})= \underset{i\ne p}{\underset{i\in [s,c_{1}]:}{\sum }} (l-i)k_{i}+(l-p)(k_{p}-1) \\& + \underset{i\ne p+1}{\underset{i\in [c_{2},l]:} {\sum }} (i-s)k_{i}+(p+1-s)(k_{p+1}+1)\\&-\bigg [\underset{i\ne p} {\underset{i\in [s,c_{1}]:}{\sum }} (l-i)k_{i}+(l-p)(k_{p})+ \\& \underset{i\ne p+1}{\underset{i\in [c_{2},l]:}{\sum }} (i-s)k_{i}+(p+1-s)(k_{p+1}) \bigg ]\\&=-(l-p)+p+1-s=-l+p+p-s+1 \\&=2p-(s+l)+1. \end{aligned}$$
  Since $p\ge (l+s)/2-1$, multiplying both sides by two, we have $2p\ge (l+s)-2$. Then we know $2p-(s+l)+1\ge -1$. Thus we conclude that $\psi (\pmb {k}'')-\psi (\pmb {k})\ge [\psi (\pmb {k}')-\psi (\pmb {k})]$. Now consider the second sub-case when $p=c_{2}$. In this case, we know $\psi (\pmb {k}'')-\psi (\pmb {k})=1$ by the conclusion in b). Since $p\ge c_{1}\ge p-1$, we have
  $$\begin{aligned} & \psi (\pmb {k}')-\psi (\pmb {k})= \underset{i\ne p-1}{\underset{i\in [s,c_{1}]:}{\sum }} (l-i)k_{i}+(l-p+1)\\&(k_{p-1}-1)+ \underset{i\ne p}{\underset{i\in [c_{2},l]:}{\sum }} (i-s)k_{i}+(p-s)(k_{p}+1)\\&-\bigg [\underset{i\ne p-1}{\underset{i\in [s,c_{1}]:}{\sum }} (l-i)k_{i}+(l-p+1)(k_{p-1}) \\& + \underset{i\ne p}{\underset{i\in [c_{2},l]:}{\sum }} (i-s)k_{i}+(p-s)(k_{p}) \bigg ]\\&=-(l-p+1)+p-s=-l+p-1\\&+p-s=2p-(s+l)-1. \end{aligned}$$
  Since $p\le (l+s)/2+1$, multiplying both sides by two, we have $2p\le (l+s)+2$, and then $2p-(l+s)-1\le 1$. Thus, we conclude that $\psi (\pmb {k}'')-\psi (\pmb {k})\ge [\psi (\pmb {k}')-\psi (\pmb {k})]$.

The above analyses show that fulfilling a request of a resident with *p* requests will result in a larger increment in the value of the inequity value (sum of maximum pairwise deviation) than fulfilling a request of a resident with $p-1$ requests. It follows that it is optimal to fulfill a request to of resident with the fewest satisfied requests. $\square$

### E-Companion Section B.4. Proof of proposition 4

#### Proposition 4

Consider iteration $j \ge 1$ of Step 3 of Algorithm 2 with inputs $V=f_1(\pmb {v}^{j-1})+1$, $\mathcal {V}^{j-1}$, and $\mathcal {U}^{j-1}$. Suppose that problem Eq. 10 with $f_1(\pmb {v})=V$ and $\mathcal {V}^{j-1}$ has an optimal solution $\pmb {v}'$ with an optimal value $f_2(\pmb {v}')$. Suppose that problem Eq. 11 with $f_1(\pmb {v})=V$ has an optimal solution $\pmb {v}^*$ with an optimal value $f_2(\pmb {v}^*)$. We have $f_2(\pmb {v}^*)=f_2(\pmb {v}')$.

#### Proof

Recall that in step 1, we solve problem Eq. 6 to obtain a Pareto optimal solution $S^0=\{(\pmb {z}^{0},\pmb {x}^{0},\pmb {v}^{0})\}$ with $f_{2}(\pmb {v}^{0})=0$ and optimal value $f_{1}(\pmb {v}^{0})$. Here, $V=f_{1}(\pmb {v}^{0})$ is the maximum number of vacation requests that can be satisfied such that the value of the inequity measure is zero, i.e., $f_{2}(\pmb {v}^{0})=0$. It follows from Proposition 1 that $(\pmb {z}^{0},\pmb {x}^{0},\pmb {v}^{0})\in \mathcal {F}_{p}$ and $(f_{1}(\pmb {v}^0),f_{2}(\pmb {v}^0))\in \mathcal {P}$. Accordingly, we enlarge the sets $\mathcal {F}_p \leftarrow \mathcal {F}_p \cup S^0$ and $\mathcal {P}\leftarrow \mathcal {P}\cup \{(f_{1}(\pmb {v}^{0}), 0)\}$. Then, we update $V=f_{1}(\pmb {v}^{0})+1$ and extract the following categories of residents from $\pmb {v}^0$: the set of residents with one or more fulfilled requests denoted as $\mathcal {V}^0$ and the set of residents with *i* satisfied vacation requests denoted as $\mathcal {U}_i^0$. In Step 2, we solve the equity-neutral model Eq. 2, record an optimal solution ($\pmb {z}, \pmb {x}, \pmb {v}$) and value $f_1(\pmb {v})$ (i.e., the maximum number of vacation requests that can be satisfied), and set $\overline{V}=f_1(\pmb {v})$. Recall that the value of $f_1(\pmb {v})$ for any non-dominated solution $(\pmb {z},\pmb {x},\pmb {v})\in \mathcal {F}_{p}$ satisfies $f_{1}(\pmb {v})\in [f_{1}(\pmb {v}^{0}),\overline{V}]_{\mathbb {Z}}$; see Remark 1. The goal of Step 3 is to identify all non-dominated rotation and vacation schedules with $f_1 \in [f_{1}(\pmb {v}^{0})+1, \overline{V}]_{\mathbb {Z}}$.

*Base Case*. Consider iteration $j=1$ of Step 3 with $V=f_{1}(\pmb {v}^{0})+1$ and sets $\mathcal {V}^0$, $\{\mathcal {U}_{i}^{0}\}_{i=0}^{\bar{w}}$, and $S^0=\{ (\pmb {z}^{0},\pmb {x}^{0},\pmb {v}^{0})\}$ as initial inputs. Suppose we solve problem Eq. 11 with $V=f_{1}(\pmb {v}^{0})+1$. The goal is to find an optimal solution $(\pmb {z}^{*}_{1},\pmb {x}^{*}_{1},\pmb {v}^{*}_{1})$ with *V* satisfied requests while minimizing the value of the inequity measure. This solution should satisfy one more vacation request than the current solution $\pmb {v}^0$, i.e.,

31 $$\begin{aligned}& f_2(\pmb {v}^*)= \underset{\pmb {z},\pmb {v},\pmb {x}}{\mathop {\mathrm {\text {minimize}}}\limits }\ \Big \{f_{2}(\pmb {v})\Big |(\pmb {z},\pmb {v},\pmb {x})\in (1b) \\& -(1m),f_{1}(\pmb {v})=V=f_{1}(\pmb {v}^{0})+1\Big \}, \nonumber \\&= \underset{\pmb {z},\pmb {v},\pmb {x}} {\mathop {\mathrm {\text {minimize}}}\limits } \ \Big \{f_2(\pmb {v}) +f_{2}(\pmb {v}^0)-f_{2}(\pmb {v}^0)|(\pmb {z},\pmb {v},\pmb {x})\in \\& \{(1b)-(1m)\}, f_{1}(\pmb {v})=V=f_{1}(\pmb {v}^{0})+1\Big \}, \nonumber \\&= f_{2}(\pmb {v}^0)+\underset{\pmb {z},\pmb {v},\pmb {x}}{\mathop {\mathrm {\text {minimize}}}\limits }\Big \{f_2(\pmb {v})-f_{2}(\pmb {v}^0)|(\pmb {z},\pmb {v},\pmb {x})\in \\& \{(1b)-(1m)\}, f_{1}(\pmb {v})=V=f_{1}(\pmb {v}^{0})+1\Big \}. \end{aligned}$$

Equality Eq. 31 holds because $f_{2}(\pmb {v}^0)$ is the minimum value of the inequity measure with $f_{1}(\pmb {v}^0)$ satisfied requests. Now, suppose we solve problem Eq. 10 with $V=f_{1}(\pmb {v}^{0})+1$, $R'$ (the set of residents with the lowest number of satisfied vacation requests from $\pmb {v}\in S^{0}$), and $\overline{W}^{\text {vac}}_{r}$ (set of unsatisfied vacation requests $\overline{W}^{\text {vac}}_{r}$ for each $r \in R'$). This problem aims to find an optimal schedule $(\pmb {z}'_{1},\pmb {x}'_{1},\pmb {v}'_{1})$ with $V= f_1(\pmb {v}^0)+1$ satisfied vacation requests that minimizes $f_2$ by approving a vacation request of one of the residents $r \in R'$, while keeping the fulfilled requests for residents $r \in \mathcal {V}^{0}$ the same as in $\pmb {v}^0 \in S^{0}$, i.e.,

32 $$\begin{aligned}& f_2(\pmb {v}'_1)= \underset{\pmb {z},\pmb {v},\pmb {x}}{\mathop {\mathrm {\text {minimize}}}\limits }\ \Big \{ f_2(\pmb {v}) \Big | (\pmb {z},\pmb {v},\pmb {x})\in \\& \{(1b)-(1m)\}, f_1(\pmb {v})=V=f_{1}(\pmb {v}^{0})+1 \nonumber \\& v_{r,d,w}=1, \forall (r,d,w)\in \mathcal {V}^{0}, \\& \sum _{r\in R'}\sum _{d\in D}\sum _{w\in \overline{W}^{\text {vac}}_{r}}v_{r,d,w}=1 \Big \}, \nonumber \\&= f_{2}(\pmb {v}^0)+ \underset{\pmb {z},\pmb {v},\pmb {x}}{\mathop {\mathrm {\text {minimize}}}\limits }\\& \ \Big \{f_{2}(\pmb {v})-f_{2}(\pmb {v}^0)\Big | (\pmb {z},\pmb {v},\pmb {x})\in \{(1b)-(1m)\},\\& f_1(\pmb {v})=V=f_{1}(\pmb {v}^{0})+1 \nonumber \hspace{45mm}\\&v_{r,d,w}=1, \forall (r,d,w)\in \mathcal {V}^{0}, \\& \sum _{r\in R'}\sum _{d\in D}\sum _{w\in \overline{W}^{\text {vac}}_{r}}v_{r,d,w}=1 \Big \} \end{aligned}$$

By Proposition 3, we know that approving a vacation request of one of the residents $r \in R'$ while keeping the fulfilled requests for residents $r \in \mathcal {V}^{0}$ the same as in $\pmb {v}^0 \in S^{0}$ leads to the smallest possible change in the value of the inequity measure. In other words, solving problem Eq. 32 (equivalently Eq. 10) leads to the minimum value of $f_{2}(\pmb {v})-f_{2}(\pmb {v}^0)$. That is to say:

33 $$\begin{aligned} f_2(\pmb {v}'_1)&=f_{2}(\pmb {v}^0)+ \underset{\pmb {z},\pmb {v},\pmb {x}}{\mathop {\mathrm {\text {minimize}}}\limits } \\& \ \Big \{f_{2}(\pmb {v})-f_{2}(\pmb {v}^0)\Big | (\pmb {z},\pmb {v},\pmb {x})\in \{(1b)-(1m)\}, \\& f_1(\pmb {v})=V=f_{1}(\pmb {v}^{0})+1 \Big \} \nonumber \\&=f_2(\pmb {v}^*_1), \end{aligned}$$

where Eq. 33 follows from Eq. 31.

Consider any iteration $j>1$ with $f_1(\pmb {v}_{j-1})$, $f_2(\pmb {v}_{j-1})$, $S^{j-1}=\{ (\pmb {z}_{j-1},\pmb {x}_{j-1},\pmb {v}_{j-1})\}$, $\mathcal {V}^{j-1}$ and $\{\mathcal {U}_{i}^{j-1}\}_{i=0}^{\bar{w}}$ as inputs. By the algorithm design, $S^{j-1}=\{ (\pmb {z}_{j-1},\pmb {x}_{j-1},\pmb {v}_{j-1})\}$ is an optimal solution to problem Eqs. be an optimal solution and value for problem

11 or 10 with $f_1(\pmb {v}_{j-1})$ satisfied vacation requests and $f_2(\pmb {v}_{j-1})$ is the minimum value of the inequity measure associated with $V=f_1(\pmb {v}_{j-1})$ satisfied requests. Let $R'$ be the set of residents with the lowest number of satisfied vacation requests from $\pmb {v}_{j-1}$ and $\overline{W}^{\text {vac}}_{r}$ be the set of unsatisfied vacation requests for each $r \in R'$.

Let $(\pmb {z}'_{j},\pmb {x}'_{j},\pmb {v}'_{j})$ and $f_2(\pmb {v}'_{j})$ be an optimal solution and value for problem Eq. 10 with $V=f_1(\pmb {v}_{j-1})+1$. Let $(\pmb {z}^{*}_{j},\pmb {x}^{*}_{j},\pmb {v}^{*}_{j})$ and $f_2(\pmb {v}^*_{j})$ be an optimal solution and value for problem Eq. 11 with $V=f_1(\pmb {v}_{j-1})+1$. We claim that

$$\begin{aligned} f_2(\pmb {v}'_j)&=f_{2}(\pmb {v}_{j-1})+ \underset{\pmb {z},\pmb {v},\pmb {x}}{\mathop {\mathrm {\text {minimize}}}\limits } \\& \ \Big \{f_{2}(\pmb {v})-f_{2}(\pmb {v}_{j-1})\Big | (\pmb {z},\pmb {v},\pmb {x})\in \{(1b)-(1m)\}, \\& f_1(\pmb {v})=f_{1}(\pmb {v}_{j-1})+1 \Big \} \nonumber \\&=f_2(\pmb {v}^*_j). \nonumber \end{aligned}$$

Since $\pmb {v}^{*}_{j}$ is an optimal solution to Eq. 11 with $V=f_1(\pmb {v}_{j-1})+1$ and $f_2(\pmb {v}_{j-1})$ is the minimum value of the inequity measure associated with $f_1(\pmb {v}_{j-1})$ satisfied vacation requests, it follows that

34 $$\begin{aligned}& f_2(\pmb {v}^*_j)= f_{2}(\pmb {v}_{j-1})+\underset{\pmb {z},\pmb {v},\pmb {x}}{\mathop {\mathrm {\text {minimize}}}\limits } \\& \Big \{f_2(\pmb {v})-f_{2}(\pmb {v}_{j-1})|(1b)-(1m),f_{1}(\pmb {v})=f_{1}(\pmb {v}_{j-1})+1\Big \}. \end{aligned}$$

Since $\pmb {v}'_{j}$ is an optimal solution to (10) with $V=f_1(\pmb {v}_{j-1})+1$, we have:

35a $$\begin{aligned}& f_2(\pmb {v}'_j)= \underset{\pmb {z},\pmb {v},\pmb {x}}{\mathop {\mathrm {\text {minimize}}}\limits }\ \Big \{ f_2(\pmb {v}) \Big | (1b)-(1m), \\& f_1(\pmb {v})=f_{1}(\pmb {v}_{j-1})+1\nonumber \hspace{30mm } \\& v_{r,d,w}=1, \forall (r,d,w)\in \mathcal {V}^{j-1}, \sum _{r\in R'}\sum _{d\in D}\sum _{w\in \overline{W}^{\text {vac}}_{r}}v_{r,d,w}=1 \Big \} \nonumber \\&= f_{2}(\pmb {v}_{j-1})+ \underset{\pmb {z},\pmb {v},\pmb {x}}{\mathop {\mathrm {\text {minimize}}}\limits }\ \Big \{f_{2}(\pmb {v})-f_{2}(\pmb {v}_{j-1})\Big | (1b)-(1m), \\& f_1(\pmb {v})=f_{1}(\pmb {v}_{j-1})+1 \nonumber \hspace{45mm}\\&v_{r,d,w}=1, \forall (r,d,w)\in \mathcal {V}^{j-1}, \sum _{r\in R'}\sum _{d\in D}\sum _{w\in \overline{W}^{\text {vac}}_{r}}v_{r,d,w}=1 \Big \} \end{aligned}$$

35b $$\begin{aligned}&=f_{2}(\pmb {v}_{j-1})+ \underset{\pmb {z},\pmb {v},\pmb {x}}{\mathop {\mathrm {\text {minimize}}}\limits }\ \Big \{f_{2}(\pmb {v})-f_{2}(\pmb {v}_{j-1}) \\& \Big | \{(1b)-(1m)\}, f_1(\pmb {v})=f_{1}(\pmb {v}_{j-1})+1 \Big \} \end{aligned}$$

35c $$\begin{aligned}&= f_2(\pmb {v}^*_j), \end{aligned}$$

where Eq. 35b follows from the fact that $f_2(\pmb {v}_{j-1})$ is the minimum value of the inequity measure associated with $f_1(\pmb {v}_{j-1})$ satisfied vacation requests and $f_{2}(\pmb {v})-f_{2}(\pmb {v}_{j-1})$ is the minimum change in inequity value by Proposition Eq. 3. Equation 35c follows from Eq. 34. This completes the proof. $\square$

## E-Companion Section C. The info template of the RRAP tool

In this appendix, we provide examples of the input info template of the RRAP tool that the user must fill out and then upload into the interface to generate a rotation schedule. This template is an Excel workbook with seven sheets, each designated for a specific set of input parameters to the IP model. Figures 8, 9 and 10 show screenshots of these sheets.

## E-Companion Section D. Additional details of RRAP instances

Tables 8, 9, and 10 respectively presents the values of paramaters $T^{{\tiny min}}_{}$ and $T^{{\tiny max}}_{}$ in Inst1, Inst2 and Inst3. Table 11 provides an example of the values of parameters $R^{{\tiny min}}_{}$ and $R^{{\tiny max}}_{}$ in Inst1 with 62.

**Table 8:** The value of $T^{{\tiny min}}_{}$ and $T^{{\tiny max}}_{}$ for each class of residents in Inst1

	PGY1		PGY2		PGY3		PGY4		PGY5	
Departments	Min	Max	Min	Max	Min	Max	Min	Max	Min	Max
Allen	1	1	1	2	0	3	1	4	0	5
Vascular	1	1	0	1	1	0	1	1	0	0
Breast	1	1	0	0	1	1	0	1	0	0
VTF	1	1	0	0	0	2	0	0	0	0
Thoracic	1	1	0	0	1	0	0	0	0	0
CR	1	1	0	0	1	1	0	0	1	0
SICU	1	1	0	0	0	2	0	0	0	1
HPB	1	1	0	0	0	0	1	0	1	0
Peds	1	1	1	0	0	0	0	1	0	1
Rainbow	1	1	0	1	0	0	0	0	0	0
Overlook	1	1	1	0	1	0	1	0	0	0
Lap	1	1	0	1	1	1	0	1	1	0
ACS-OR	0	1	1	0	0	1	0	0	0	1
Consults	0	0	3	1	0	0	0	0	0	0
CTICU	0	0	1	3	0	0	0	0	0	0
Renal	0	0	0	1	1	0	0	0	0	0
Trauma	0	0	0	0	1	1	0	0	0	0
Elective	0	0	0	0	0	2	1	0	1	0
ACS	0	0	0	0	0	0	1	1	0	1
Nights	0	0	0	0	0	0	1	1	1	0
HPB-Chabot	0	0	0	0	0	0	0	1	1	1

**Table 9:** The value of $T^{{\tiny min}}_{}$ and $T^{{\tiny max}}_{}$ for each class of residents in Inst2

	PGY1		PGY2		PGY3		PGY4		PGY5	
Departments	Min	Max	Min	Max	Min	Max	Min	Max	Min	Max
Allen	1	1	1	1	1	1	1	1	1	1
Vascular	1	1	1	1	1	1	1	1	1	1
Breast	1	1	1	1	1	2	1	1	1	1
VTF	1	1	1	1	1	1	1	1	1	1
Thoracic	1	1	1	1	1	1	1	1	1	1
CR	1	1	1	1	1	2	1	1	1	1
SICU	1	1	1	1	1	1	1	1	1	1
HPB	1	1	1	1	1	1	1	1	1	1
Peds	1	1	1	1	1	1	1	1	1	1
Rainbow	1	1	1	1	1	1	1	1	1	1
Overlook	1	1	1	1	1	1	1	1	1	1
Lap	1	1	1	1	1	1	1	1	1	1

**Table 10:** The value of $T^{{\tiny min}}_{}$ and $T^{{\tiny max}}_{}$ for each class of residents in Inst3

	PGY1		PGY2		PGY3		PGY4		PGY5	
Departments	Min	Max	Min	Max	Min	Max	Min	Max	Min	Max
Allen	1	1	1	1	1	1	1	1	1	1
Vascular	1	1	1	1	1	1	1	1	1	1
Breast	1	1	1	1	1	1	1	1	1	1
VTF	1	1	1	1	1	1	0	0	0	0
Thoracic	1	1	1	1	1	1	1	1	1	1
CR	1	1	1	1	1	1	1	1	1	1
SICU	1	1	0	0	1	1	1	1	1	1
HPB	1	1	1	1	1	1	1	1	0	0
Peds	1	1	0	0	0	0	0	0	0	0
Rainbow	1	1	0	0	0	0	0	0	0	0
Overlook	1	1	1	1	1	1	1	1	0	0
Lap	1	1	0	0	0	0	1	1	1	1
ACS	0	0	1	1	0	0	0	0	1	1
Consults	0	0	3	3	0	0	0	0	0	0
Trauma	0	0	0	0	1	1	0	0	0	0
Nights	0	0	0	0	0	0	1	1	1	1

**Table 11:** An example of the value of $R^{{\tiny min}}_{}$ and $R^{{\tiny max}}_{}$ for each class of residents

	PGY1		PGY2		PGY3		PGY4		PGY5	
Departments	Min	Max	Min	Max	Min	Max	Min	Max	Min	Max
Allen	1	3	1	2	0	0	1	1	0	0
Vascular	1	4	0	0	0	1	0	1	0	0
Renal	0	1	0	0	0	1	0	0	0	0
AdultAnes	0	1	0	0	0	0	0	0	0	0
Lap	1	3	0	0	0	1	0	0	0	1
Rainbow	1	3	0	0	0	0	0	0	0	0
ED	0	2	0	0	0	0	0	0	0	0
Breast	0	3	0	0	0	2	0	0	0	0
SICU	1	4	0	0	0	0	0	0	0	0
Plastics	0	2	0	0	0	0	0	0	0	0
Thoracic	1	4	0	0	0	2	0	0	0	0
OMFS	0	1	0	0	0	0	0	0	0	0
PMR	0	1	0	0	0	0	0	0	0	0
HPB	1	3	0	0	0	0	1	1	1	1
ENT	0	1	0	0	0	0	0	0	0	0
Overlook	1	3	0	3	0	2	1	1	0	0
CR	1	3	0	0	0	1	0	0	1	1
ACS	0	2	0	0	0	0	1	1	0	0
VTF	1	3	0	0	0	0	0	0	0	0
Peds	1	4	0	2	0	0	0	0	0	0
PedsAnes	0	1	0	0	0	0	0	0	0	0
ACS-OR	0	0	0	2	0	0	0	0	0	0
Consults	0	0	1	4	0	0	0	0	0	0
Trauma	0	0	0	0	0	1	0	0	0	0
Nights	0	0	0	0	0	0	1	1	1	1
Elective	0	4	0	0	0	0	0	1	0	1
HPB-Chabot	0	0	0	0	0	0	0	0	1	1
CTICU	0	0	1	3	0	0	0	0	0	0

## E-Companion Section E. Details of CPU time of equity-promoting model

Table 12 presents solution times in each step of Algorithm 2.

**Table 12:** Total time (in seconds) in each step of Algorithm 2

Instance	Range			Gini			MeanDev			MaxMeanDev			SumMaxPair		
	Step 1	Step 2	Step 3	Step 1	Step 2	Step 3	Step 1	Step 2	Step 3	Step 1	Step 2	Step 3	Step 1	Step 2	Step 3
Inst1-62-A	3.6	3.1	133.3	4.6	3.6	197.8	3.9	3.4	185.2	3.9	3.3	141.8	4.5	3.8	176.2
Inst1-62-B	7.7	6.0	194.3	8.4	6.0	337.7	7.0	5.3	431.9	6.9	5.4	209.5	8.0	5.9	291.5
Inst2-62-A	2.5	3.1	110.9	1.5	2.9	114.6	2.2	2.4	108.8	2.2	2.4	75.1	3.1	2.4	113.0
Inst2-62-B	3.5	4.8	135.6	5.4	3.0	122.4	4.1	4.5	108.4	4.1	4.5	76.8	6.4	4.8	93.4
Inst3-62-A	2.3	4.3	103.2	3.1	5.6	171.0	2.5	4.8	153.7	2.5	4.7	117.1	3.1	5.6	146.4
Inst3-62-B	2.2	2.5	149.6	5.0	4.3	636.0	1.9	2.6	66.5	1.9	2.6	61.1	2.4	2.9	182.3
Inst1-125-A	9.8	8.8	539.2	15.5	11.0	1072.4	13.2	9.1	942.9	13.9	9.6	775.4	15.4	11.0	967.8
Inst1-125-B	13.3	9.9	896.8	10.7	8.5	623.9	14.3	9.5	986.4	14.2	9.5	906.1	14.2	9.6	906.1
Inst2-125-A	19.2	22.1	658.0	21.1	21.7	2496.2	18.0	20.9	1438.7	17.9	21.2	1134.2	21.0	22.2	906.2
Inst2-125-B	44.3	28.8	1176.9	43.1	27.9	2931.7	40.2	27.0	1818.6	43.0	27.1	1241.2	43.3	28.2	1101.1
Inst3-125-A	7.9	6.9	510.8	12.0	9.0	1222.5	9.6	7.6	1003.2	9.4	7.5	860.2	11.8	9.0	932.2
Inst3-125-B	14.6	8.0	681.7	14.9	8.5	1201.5	12.8	7.3	956.3	13.7	7.9	818.0	16.8	8.8	855.1
Inst1-185-A	11.2	10.06	1188.3	26	15	3667.1	24.6	12.2	3061.4	23.3	12.3	1915.6	15.4	26.4	950.7
Inst1-185-B	22.6	15.8	1599.2	30.1	18.0	3950.6	30.5	15.7	3563.4	29.1	15.5	2199.8	33.6	21.4	3381.9
Inst2-185-A	20.4	13.1	1316.8	28.7	14.8	5613.6	23.4	12.9	3052.3	23.3	14.7	5248.1	29.2	15.3	2187.7
Inst2-185-B	17.2	8.8	1746.1	20.2	11.6	5668.5	18.2	10.0	3616.5	18.2	9.7	2071.6	21.3	11.9	2697.7
Inst3-185-A	8.5	6.0	903.0	18.5	10.7	3635.5	14.8	8.1	2335.5	14.8	8.1	1789.5	19.0	10.9	2022.0
Inst3-185-B	125.4	59.6	2747.2	115.8	64.8	9718.2	111.5	55.2	7138.0	103.8	36.8	2755.4	116.7	59.6	5423.4

## E-Companion Section F. Details of CPU time of formulations Eqs. 1b and 3

Tables 13 and 14 respectively present solution times of formulations Eqs.1a and 2.

**Table 13:** Solution time (in seconds) of formulation Eq. 1b

Instance	Min	Avg	Max
Inst1-62	0.68	0.69	0.70
Inst1-125	2.09	2.12	2.15
Inst1-185	3.38	3.41	3.44
Inst2-62	0.71	0.72	0.73
Inst2-125	2.81	2.87	3.09
Inst2-185	4.42	4.46	4.50
Inst3-62	0.63	0.65	0.66
Inst3-125	3.22	3.26	3.30
Inst3-185	7.37	7.41	7.45

**Table 14:** Solution time (in seconds) using the equity-neutral model

Instance	Time	Instance	Time	Instance	Time
Inst1-62-A	3.1	Inst1-125-A	8.8	Inst1-185-A	10.1
Inst1-62-B	6.0	Inst1-125-B	9.9	Inst1-185-B	15.8
Inst2-62-A	3.1	Inst2-125-A	22.1	Inst2-185-A	13.1
Inst2-62-B	4.8	Inst2-125-B	28.8	Inst2-185-B	8.8
Inst3-62-A	4.3	Inst3-125-A	6.9	Inst3-185-A	6.0
Inst3-62-B	2.5	Inst3-125-B	8.0	Inst3-185-B	59.6

## E-Companion Section G. Details of the RRAP-Case instance

We construct the RRAP-Case instance based on the data related to the CUIMC’s 2023–2024 academic year. As mentioned in Section 7.1, this instance consists of 54 residents, of which (22, 9, 9, 7, 7) are (PGY1, PGY2, PGY3, PGY4, PGY5) residents. There are five types of PGY1 residents, PGY1-Categorical, PGY1-OMFS, PGY1-Prelim, PGY1-GU and PGY1-Ortho. In addition, PGY2 and PGY3 are either Categorical or Cardiac. The number of blocks for (PGY1, PGY2, PGY3, PGY4, PGY5) is (12, 8, 9, 7, 7) and the number of weeks in each block is (4 to 6, 6 to 7, 5 to 6, 7 to 8, 7 to 9). Table 15 summarizes mandatory (required), possible, and busy departments for each type of resident. Mandatory departments are underlined, and busy departments are highlighted in bold text. Tables 16 and 17 present the value of parameters $T^{{\tiny min}}_{}$ and $T^{{\tiny max}}_{}$ for PGY1 and PGY2–PGY5 residents, respectively. Table 18 presents the values of parameters $R^{{\tiny min}}_{}$ and $R^{{\tiny max}}_{}$.

**Table 15:** Mandatory (underlined), possible, and busy departments (bold) for each type of resident

Resident Type	Departments
PGY1-Categorical	Allen, CR, HPB, Lap,Overlook, Peds, SICU,Thoracic, Vascular, **Rainbow**, **VTF**,
ACS, Breast	
PGY1-Prelim	ACS, Breast, Lap, Overlook, Thoracic, Vascular, Peds, HPB, Allen
PGY1-GU	ACS, ED,Vascular, CR, Transplant, SICU, **Rainbow**, HPB, Thoracic
PGY1-OMFS	ENT, SICU, AdultAnes, PedsAnes, Plastics, Peds, OMFS, Thoracic, Vascular, **Rainbow**, **VTF**
PGY1-Ortho	Vascular, Plastics, SICU, PMR, Breast, ED,
PGY2-Categorical	Consults, CTICU, Overlook, Peds, **ACS**, **Allen**
PGY2-Cardiac	Cardiac, CTICU, Overlook, Consults, **ACS**, **Allen**
PGY3	Breast, CR, Lap, Overlook, Transplant, **Thoracic**, **Trauma**, **Vascular**
PGY3-Cardiac	Cardiac, Overlook, Breast< **Thoracic**, **Vascular**
PGY4	ACS, Allen, Elective, HPB, Overlook, **Nights**, **Vascular**
PGY5	CR, Elective, HPB-Chabot, HPB, Lap, Floats, **Nights**

**Table 16:** The value of $T^{{\tiny min}}_{}$ and $T^{{\tiny max}}_{}$ for PGY1 residents

	PGY1-Categorical		PGY1-OMFS		PGY1-Prelim		PGY1-GU		PGY1-Ortho	
Departments	Min	Max	Min	Max	Min	Max	Min	Max	Min	Max
Allen	1	1	0	0	0	1	0	0	0	0
CR	1	1	0	0	0	0	1	1	0	0
HPB	1	1	0	0	0	1	0	1	0	0
Lap	1	1	0	0	1	2	0		0	0
Rainbow	1	1	1	1	0	0	1	1	0	0
VTF	1	1	1	1	0	0	0	0	0	0
Overlook	1	1	0	0	1	1	0	0	0	0
Peds	1	1	1	1	0	1	0	0	0	0
SICU	1	1	1	1		0	1	1	1	1
Thoracic	1	1	1	2	1	2	0	1	0	0
Vascular	1	1	1	1	1	2	1	1	1	1
ACS	0	1	0	0	1	2	1	1	0	0
Breast	0	1	0	0	1	1	0	0	0	1
ENT	0	0	1	1	0	0	0	0	0	0
AdultAnes	0	0	1	1	0	0	0	0	0	0
PedsAnes	0	0	1	1	0	0	0	0	0	0
Plastics	0	0	1	2	0	0	0	0	1	1
OMFS	0	0	1	1	0	0	0	0	0	0
ED	0	0	0	0	0	0	1	1	0	1
Transplant	0	0	0	0	0	0	1	1	0	0
PMR	0	0	0	0	0	0	0	0	1	1

**Table 17:** The value of $T^{{\tiny min}}_{}$ and $T^{{\tiny max}}_{}$ for PGY2–PGY5 residents

	PGY2		PGY2-Cardiac		PGY3		PGY3-Cardiac		PGY4		PGY5	
Departments	Min	Max	Min	Max	Min	Max	Min	Max	Min	Max	Min	Max
Consults	3	3	1	2	0	0	0	0	0	0	0	0
ACS	1	1	1	1	0	0	0	0	1	1	0	0
Allen	1	1	0	1	0	0	0	0	1	1	0	0
CTICU	1	1	1	1	0	0	0	0	0	0	0	0
Overlook	1	1	1	1	1	1	1	1	1	1	0	0
Peds	1	1	0	0	0	0	0	0	0	0	0	0
Cardiac	0	0	3	3	0	0	4	5	0	0	0	0
Breast	0	0	0	0	1	1	0	1	0	0	0	0
CR	0	0	0	0	1	2	0	0	0	0	1	1
Lap	0	0	0	0	1	2	0	0	0	0	1	1
Transplant	0	0	0	0	1	1	0	0	0	0	0	0
Thoracic	0	0	0	0	1	1	1	2	0	0	0	0
Trauma	0	0	0	0	1	2	0	0	0	0	0	0
Vascular	0	0	0	0	1	1	1	1	1	1	0	0
Elective	0	0	0	0	0	0	0	0	1	1	1	1
HPB	0	0	0	0	0	0	0	0	1	1	1	1
Nights	0	0	0	0	0	0	0	0	1	1	1	1
Chatbot	0	0	0	0	0	0	0	0	0	0	1	1
Floats	0	0	0	0	0	0	0	0	0	0	1	1

**Table 18:** The value of $R^{{\tiny min}}_{}$ and $R^{{\tiny max}}_{}$ for each class of residents

	PGY1		PGY2		PGY3		PGY4		PGY5	
Departments	Min	Max	Min	Max	Min	Max	Min	Max	Min	Max
Allen	0	1	1	2	0	0	1	1	0	0
Vascular	2	3	0	0	1	1	1	1	0	0
Transplant	0	1	0	0	0	1	0	0	0	0
AdultAnes	0	2	0	0	0	0	0	0	0	0
Lap	1	2	0	0	1	1	0	0	1	1
Rainbow	1	1	0	0	0	0	0	0	0	0
ED	0	2	0	0	0	0	0	0	0	0
Breast	1	2	0	0	0	1	0	0	0	0
SICU	1	3	0	0	0	0	0	0	0	0
Plastics	0	1	0	0	0	0	0	0	0	0
Thoracic	1	2	0	0	1	2	0	0	0	0
OMFS	0	1	0	0	0	0	0	0	0	0
PMR	0	1	0	0	0	0	0	0	0	0
HPB	1	2	0	0	0	0	1	1	1	1
ENT	0	1	0	0	0	0	0	0	0	0
Overlook	0	2	1	2	1	1	0	1	0	0
CR	0	1	0	0	1	2	1	1	1	1
ACS	1	1	1	2	0	0	1	1	0	0
VTF	0	1	0	0	0	0	0	0	0	0
Peds	1	3	0	1	0	0	0	0	0	0
PedsAnes	0	1	0	0	0	0	0	0	0	0
Consults	0	0	2	3	0	0	0	0	0	0
Trauma	0	0	0	0	1	1	0	0	0	0
Nights	0	0	0	0	0	0	1	1	1	1
Elective	0	0	0	0	0	0	1	1	1	1
HPB-Chabot	0	0	0	0	0	0	0	0	1	1
CTICU	0	0	1	2	0	0	0	0	0	0
Floats	0	0	0	0	0	0	0	0	1	1
Cardiac	0	2	0	1	1	1	0	0	0	0

## Footnotes

1 Note that there are typically eight types of PGY1 residents: Categorical, Prelim, GU, OMFS, Ortho, Cardiac, ENT, and Plastics. We do not include Cardiac, ENT, and Plastics in this illustrative instance.
